# Development of suicide postvention guidelines for secondary schools: a Delphi study

**DOI:** 10.1186/s12889-016-2822-6

**Published:** 2016-02-24

**Authors:** Georgina R. Cox, Eleanor Bailey, Anthony F. Jorm, Nicola J. Reavley, Kate Templer, Alex Parker, Debra Rickwood, Sunil Bhar, Jo Robinson

**Affiliations:** Orygen, the National Centre of Excellence in Youth Mental Health and the Centre for Youth Mental Health, University of Melbourne, 35 Poplar Rd, Parkville, Victoria 3052 Australia; Centre for Mental Health, The Melbourne School of Population and Global Health, University of Melbourne, 207 Bouverie Street, Parkville, Victoria 3010 Australia; University of Tasmania, Faculty of Health, Hobart, Tasmania 7001 Australia; headspace National Youth Mental Health Foundation, 485 La Trobe St, Victoria, 3000 Australia; Faculty of Health, University of Canberra, Kirinari St, Bruce, Australian Capital Territory, 2601 Australia; Faculty of Health, Arts and Design, School of Health Sciences, Department of Psychological Sciences, Swinburne University of Technology, John Street, Hawthorn, Victoria 3122 Australia

**Keywords:** Suicide, Postvention, Schools, Delphi method, Expert consensus

## Abstract

**Background:**

Suicide of school-aged adolescents is a significant problem, with serious implications for students and staff alike. To date, there is a lack of evidence regarding the most effective way for a secondary school to respond to the suicide of a student, termed postvention [(Crisis 33:208-214, 2012), (Crisis 34:164-182, 2013)]. The aim of this study was to employ the expert consensus (Delphi) methodology to the development of a set of guidelines, to assist English-speaking secondary schools to develop a plan to respond to a student suicide, or to respond to a suicide in the absence of a predetermined plan.

**Methods:**

The Delphi methodology was employed, which involved a two-stage process. Firstly, medical and research databases, existing postvention guidelines developed for schools, and lay literature were searched in order to identify potential actions that school staff could carry out following the suicide of a student. Based on this search, an online questionnaire was produced. Secondly, 40 experts in the area of suicide postvention from English-speaking countries were recruited and asked to rate each action contained within this questionnaire, in terms of how important they felt it was to be included in the postvention guidelines. A set of guidelines was developed based on these responses. In total, panel members considered 965 actions across three consensus rounds.

**Results:**

Five hundred fourty-eight actions were endorsed for inclusion into the postvention guidelines based on an 80 % consensus agreement threshold. These actions were groups according to common themes, which are presented in the following sections: 1. Developing an Emergency Response Plan; 2. Forming an Emergency Response Team; 3. Activating the Emergency Response Team; 4. Managing a suspected suicide that occurs on school grounds; 5. Liaising with the deceased student’s family; 6. Informing staff of the suicide; 7. Informing students of the suicide; 8. Informing parents of the suicide; 9. Informing the wider community of the suicide; 10. Identifying and supporting high-risk students; 11. Ongoing support of students; 12. Ongoing support of staff; 13. Dealing with the media; 14. Internet and social media; 15. The deceased student’s belongings; 16. Funeral and memorial; 17. Continued monitoring of students and staff; 18. Documentation; 19. Critical Incident Review and annual review of the ER Plan; 20. Future prevention. Panel members frequently commented on every suicide being ‘unique’, and the need for flexibility in the guidelines, in order to accommodate the resources available, and the culture of the school community.

**Conclusion:**

In order to respond effectively and safely to the suicide of a student, schools need to undertake a variety of postvention actions. These are the first set of postvention guidelines produced worldwide for secondary schools that are based on expert opinion using the Delphi method.

**Electronic supplementary material:**

The online version of this article (doi:10.1186/s12889-016-2822-6) contains supplementary material, which is available to authorized users.

## Background

Suicide is amongst the leading causes of death worldwide in adolescents aged 10-19 years (World Health Organisation (WHO), [1]). Many young people who die by suicide are likely to be attending school, which has a significant impact on both students and staff. For example, exposure to the suicide of another student can produce or exacerbate feelings of depression, suicidal ideation and, in some cases, symptoms of post-traumatic stress disorder in other students [2–5]. In addition, young people who have lost a peer to suicide describe feeling guilty at not having recognised the signs of suicide, or having missed the opportunity to intervene [6, 7], making the emotional processing of the event extremely challenging. Young people are also particularly susceptible to the phenomenon of suicide contagion and the suicide of a student may start, or contribute to, a suicide cluster within the wider school community [8]. With regard to school staff, school counsellors have reported not feeling adequately prepared for the suicide of a student, and having little or no professional support to help them deal with the emotional impact of the situation [9].

The term suicide *postvention* has been defined as “*activities developed by, with or for suicide survivors, in order to facilitate recovery after suicide and to prevent adverse outcomes including suicidal behaviour*” ([10], p.43). Over the last 20 years, a number of postvention guidelines have been developed to help schools responding to the suicide of a student (American Foundation for Suicide Prevention (AFSP), [11], headspace, [12]). Amongst other information, they contain guidance on how to inform students, parents and the wider community of a suicide, how to support students and staff in both the short and longer term, as well as providing guidance on managing funerals and memorials. These activities are thought to be important in aiding the school community to repair, and reach an equilibrium in the months following a suicide [13]. Resources such as these are often developed in consultation with experts in the field of suicide prevention and postvention, as well as with professionals who work with schools following a student suicide.

Although these resources are an essential support for schools in terms of planning their response to a student suicide, and responding to one should it occur, there is limited research into what types of postvention activities are most effective in this situation. A systematic review of school-based postvention activities found just two trials that had evaluated the effectiveness of such approaches [14]. One trial found that school-based counselling showed limited effectiveness in terms of reducing risk in the participating students in the long term [15]. The other trial observed that a ‘first talk through’ (FTT) and psychological debriefing meeting may be of benefit to students, and recommended screening to be undertaken with students in order to identify those who may be at risk of Post-Traumatic Stress Disorder [2]. Other observational studies have also examined school-based responses following suicide clusters in young people [16]. These have included educational debriefings giving young people information about suicide, suicide prevention and coping strategies, and individual screening and referral of young people identified as being at-risk for suicide to local mental health services [17, 18]. Despite some efforts to implement longitudinal evaluation of such activities, it is unclear which individual approaches are most effective and/or helpful in curbing suicide clusters in school settings [18].

The lack of trials conducted in the area of school suicide postvention highlights the ethical and practical issues inherent in conducting this type of research. For example, the low base rate of suicide and unpredictability of the event in a school setting make it especially problematic to prospectively design studies that adequately test the effectiveness of specific interventions, while allowing for ethical approval to be obtained within a short timeframe. In addition, withholding potentially helpful interventions to a school population that may contain young people experiencing suicidal ideation presents an ethical dilemma [19, 20].

This lack of evidence calls into question the validity of current guidelines, in terms of the degree to which their guidance is ‘evidence-based’. In the absence of such rigorously designed and evaluated trials providing evidence to inform postvention activities in a school setting, the Delphi method is an alternative means by which to gather expert opinion on what approaches are likely to be most helpful and effective in this context, and is often used when scientific knowledge, or evidence, is lacking [21]. This method uses a consensus approach, underpinned by the notion of the ‘wisdom of crowds’, whereby under certain conditions, groups are able to make good judgements.

Given the detrimental effects on the school community and the lack of evidence in this area, this study uses the Delphi method to produce a set of ‘best practice’ postvention guidelines for secondary schools to use to help them develop a plan to respond to the suicide of a student, or to respond to a suicide in the absence of a predetermined plan.

## Method

### The Delphi method

The Delphi method involves a group of experts (hereafter termed panel members) in a specific area making a series of ratings regarding various statements or actions. This is done independently in the first instance, so that they draw on their own knowledge and expertise. After data from the initial rating round is obtained, panel members receive feedback on statements or actions that have been endorsed by the whole group, and also those that did not reach a predetermined level of consensus. They are then asked to engage in a second round where they have the opportunity to change or maintain their original rating. These rounds continue until consensus has been reached. Overall, the process involves two steps: literature search and questionnaire development, and the Delphi process. Ethical approval for the study was granted from the Swinburne University Research Ethics Committee (SUREC), project number 2013/174, and the University of Melbourne Behavioural and Social Sciences Human Ethics Sub-Committee, project number 1238588. All panel members gave informed consent, in accordance within the ethics procedure, in order to participate.

### Literature search and questionnaire development

The aim of the literature search was to locate documents containing any action that staff members in a secondary school could carry out, or had carried out, following the suicide of a student. By *secondary school*, we refer to schools containing adolescents aged between 13 and 18 years. Documents containing actions carried out by primary or elementary schools were excluded, as were postvention actions targeted predominantly at young people under the age of 13 years, or those in university settings where young people are generally over 18 years. Documents which described the prevention of suicide in schools, referred to actions following a suicide attempt only, or specific gatekeeper training programs were also excluded. The review was carried out across the medical and research literature, and the lay literature, which encompassed information in the public domain available on the internet, such as existing postvention guidelines, websites, or presentations.

Medical and research databases Medline, PsycINFO and Embase were searched in March 2013, with the following words forming the basis of the search: suicid* AND (School OR academic OR curriculum OR education OR after OR post* OR follow*). Searches were performed with no language or time limits. Titles and abstracts of the 5169 articles initially retrieved were screened for relevance, from which 58 were deemed eligible for further inspection and the full text was retrieved. Of these 58 articles, 40 were included and actions extracted from.

To locate lay literature relating to postvention in schools, websites were retrieved through the search engine google, and included information from google.com (USA), google.com.au (Australia), google.co.uk (UK), google.co.nz (New Zealand) and google.ca (Canada). The following words formed the basis of the search: Suicid* AND school AND (postvention OR post OR follow OR after OR guidelines OR recommendations). The first 50 websites from each search engine were retrieved, as after this point, the quality of websites is believed to decline and duplicates become much more frequent [22]. The 250 websites retrieved were screened for relevance and duplicates were removed. The remaining 53 websites were then screened for specific actions that a school could take following the suicide of a student. Any relevant links on websites were also followed, and the same procedure was employed. From the websites considered, all 53 contained actions that could be extracted for further discussion. A further six documents were also obtained through personal communication with experts in the field, or from mailing lists that the authors were subscribed to, and three books suitable for inclusion were located through Amazon.com.

In order to construct the questionnaire, three members of the research team (GC, KT and EB) grouped actions into the following categories: Development of the Emergency Response Plan and Emergency Response Team; Managing a suspected suicide that occurs on school grounds; Confirming facts; Activating the Emergency Response Team; Liaising with the deceased’s family; Informing staff; Informing students; Informing parents; Informing the wider community; Dealing with the media; High-risk students; Supporting students; Supporting staff; The deceased’s belongings; Funeral and memorial; Social media; Continued monitoring; Documenting actions; Critical incident review and Future prevention. These categories were based upon those used in previous postvention documents. In the second consensus round, an additional category was added, ‘Language to use when talking about suicide in a school setting’, based on the feedback from panel members. Actions that were similar in nature or that appeared multiple times across documents were only included in the questionnaire once.

A working group was formed, comprising members of the research team who were either experts in undertaking research using the Delphi method, or in the field of suicide prevention (GC, JR, EB, AFJ, and NR). The working group met regularly to discuss each possible action that had been extracted from the literature search and could be included in the questionnaire. They sought to ensure that each action could be carried out by a member of a school community, that only one idea was conveyed within an action, and that each action was both clear and unambiguous. For example, the action ‘*If someone is hurting, try not to leave them alone*’ is stated more clearly as ‘*If a student is highly distressed, a member of staff should remain with them*’. All possible actions from the literature search were therefore rewritten to be clear instructions. Actions which could not be carried out by a member of the school community (e.g. those that a relevant education department were responsible for), or could not be easily interpreted by the working party, were not included in the questionnaire.

### Panel formation

Expert panel members were identified through the literature search and through the authors’ professional networks. Snowball sampling was also used, by asking panel members to identify other people who they felt met the inclusion criteria for participation in the study. Panel members had to be English-speaking researchers or professionals from a developed country and fulfil one of the following criteria: 1. Authored papers on suicide postvention from the year 2000 onwards; 2. Been named as a contributor in a set of suicide postvention guidelines for schools; 3. Consulted with more than one school to support them following the suicide of a student, or to assist them in developing a plan in case of a student suicide or; 4. Worked in a leadership capacity and/or been part of an Emergency Response Team (a group of individuals who generally coordinate and lead a schools response to a suicide) at a school which has experienced a student suicide in the past five years (e.g. principal or assistant principal, school wellbeing co-ordinator).

### Statement selection

#### The Delphi process

The final questionnaire was designed and distributed to panel members using the online survey software ‘SurveyMonkey’. The questionnaire was designed in such a way that panel members had to answer each question before proceeding to the next, to ensure there was no missing data from skipped questions. Panel members were able to save their answers and come back to finish the questionnaire at a later date.

In the first round questionnaire, panel members were asked to rate each action according to whether they believed it should be included in the postvention guidelines, with the options being presented on a 5-point scale as- *essential, important*, *unimportant*, *should not be included or don't know*/*depends*. They were also given the opportunity to suggest new actions and comment on the wording of any existing actions. Responses from the first round were then reviewed by the working group and any new actions that the group felt were appropriate for rating were included in the second round questionnaire. Statements rated as *essential* or *important* by 80 % of panel members in round one were included in the guidelines. This cut off was chosen in line with previous studies using the Delphi methodology [23]. In the second round questionnaire, panel members were asked to rate any new actions suggested by other panel members, and to re-rate actions which were endorsed by 70-79 % of panel members as being *essential* or *important* to include in the guidelines. At each stage, participants were sent a list of the actions along with their initial rating, and the percentage of panel members that had rated the action as being either *essential* or *important* to include, and advised that they were able to change their original response based on this information if they would like. They were also sent a list of the actions that were endorsed and rejected by panel members.

## Results

Table 1 shows the demographic characteristics and experience of panel members. In the first round, 40 panel members participated, in the second round there were 26 and in the third round 25. Panel members came from a variety of academic and professional backgrounds: 37.5 % identified as having consulted with more than one secondary school following the suicide of a student or assisted in developing a plan in case of a future student suicide, 27.5 % identified as having worked in a leadership capacity and/or been part of an Emergency Response Team at a secondary school that had experienced a student suicide in the past five years, 22.5 % identified as having authored a peer-reviewed paper in the area of suicide postvention from the year 2000 onwards and 12.5 % as having been named as a contributor in a set of suicide postvention guidelines for secondary schools.

**Table 1:** Panel members’ demographics and experience

Variables	Total
	*N* = 40 (%)
Gender	
Female	27 (67.5 %)
Male	13 (32.5 %)
Age	Mean = 48.1 years, SD = 10.3
Current country of residence	
Australia	34 (85 %)
New Zealand	1 (2.5 %)
USA	5 (12.5 %)
Number of times been involved in a postvention response of a student	
0	4 (10 %)
1	2 (5 %)
2	7 (17.5 %)
3	5 (12.5 %)
4	4 (10 %)
5	1 (2.5 %)
5+	17 (42.5 %)

**Fig. 1:**
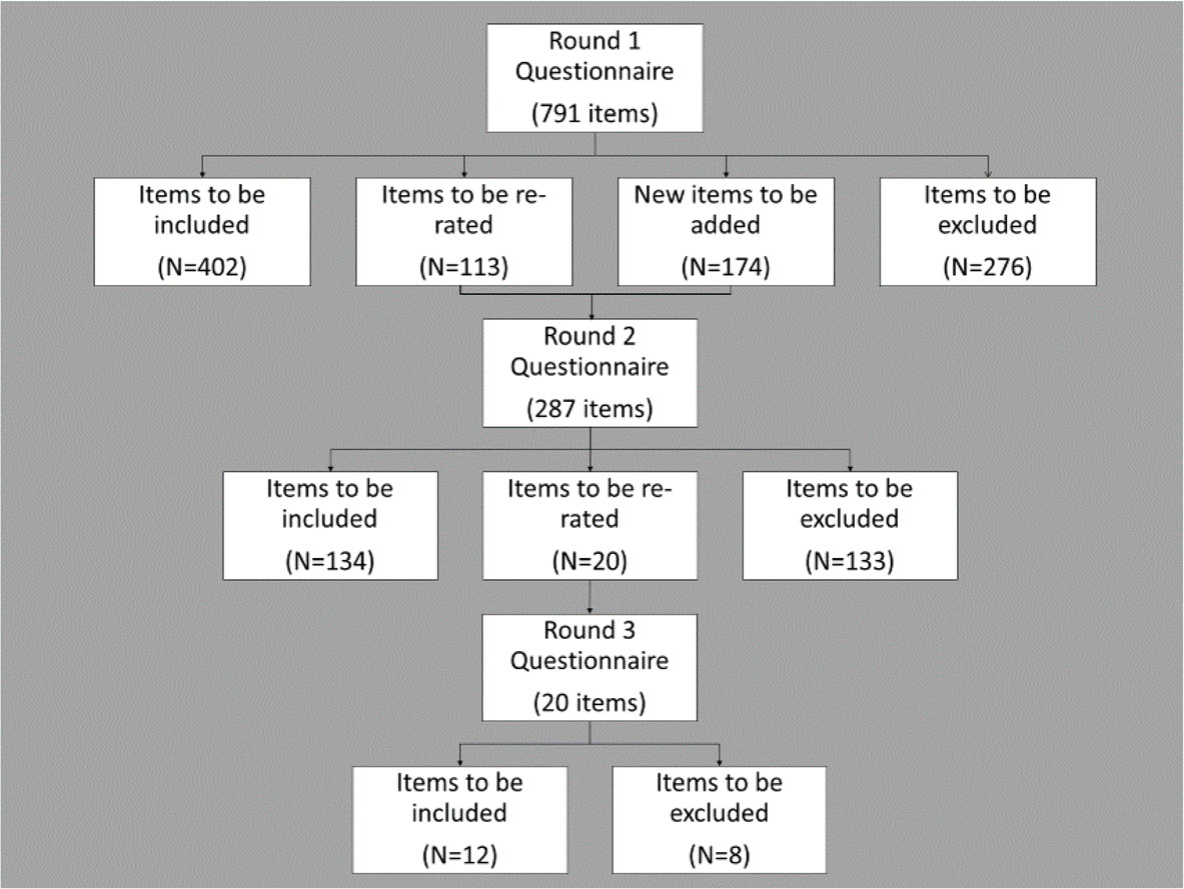
Flow of actions through the delphi consensus process

In total, 3949 actions were extracted from documents retrieved from the literature search. Figure 1 shows the number of actions included in the guidelines, those rejected, and those that were to be re-rated, at each stage of the Delphi process. The full list of all endorsed and rejected actions can be found in Additional file 1.

### Writing the guidelines

The 548 endorsed actions from all three consensus rounds were rewritten into a ‘postvention guidelines’ document by the authors, in a way that would be accessible and clear for school staff to follow (see http://headspace.org.au/assets/School-Support/hSS-Delphi-Study-web.pdf for a copy of the guidelinesfor a copy of the guidelines). Actions were grouped according to common themes, and presented within the following sections: 1. Developing an ER Plan; 2. Forming an ER Team; 3. Activating the ER Team; 4. Managing a suspected suicide that occurs on school grounds; 5. Liaising with the deceased student’s family; 6. Informing staff of the suicide; 7. Informing students of the suicide; 8. Informing parents of the suicide; 9. Informing the wider community of the suicide; 10. Identifying and supporting high-risk students; 11. Ongoing support of students; 12. Ongoing support of staff; 13. Dealing with the media; 14. Internet and social media; 15. The deceased student’s belongings; 16. Funeral and memorial; 17. Continued monitoring of students and staff; 18. Documentation; 19. Critical Incident Review and annual review of the ER Plan; 20. Future prevention

Rather than list all of the actions, items were combined to form a coherent document. Actions could be reworded, as long as the main message contained within them did not change. Consider the following actions as an example:

*‘The Emergency Response Team should ensure that any memorial sites or activities do not glorify, vilify or stigmatise the deceased student or their death’.*

*‘The Emergency Response Team should ensure that any memorial sites or activities are the same as they would be for a non-suicide death’.*

*‘The Emergency Response Team should ensure that any memorial site or activity is culturally appropriate’.*

These were rewritten into the following text:

*‘The Emergency Response Team should ensure that any memorial sites or activities do not glorify, vilify or stigmatise the deceased student or their death, and are the same as they would be for a non-suicide death. They should also ensure that any memorial site or activity is culturally appropriate’.*

We sought to ensure that relevant concepts were grouped together, and were presented in a clear, and concise manner. For example consider the following actions:

*‘The Emergency Response Plan should be implemented in response to a suspected or confirmed suicide of a current student’.*

*‘The Emergency Response Plan should be implemented in response to a suspected or confirmed suicide of a student who is enrolled at the school but is currently not attending (e.g. on exchange, on extended sick leave)’.*

*‘The Emergency Response Plan should make provisions for: Providing support for students in the holidays when a death by suicide occurs outside the school year and handling the death of a recently graduated student’.*

As the concepts contained within all of these actions related to *when* to implement the Emergency Response Plan, these were grouped together and presented in the following way:

*When should the school implement the Emergency Response Plan?*

- In response to a suspected or confirmed suicide of a student. This could be a current student or a student who is enrolled at the school but is currently not attending (e.g. on exchange, on extended sick leave)
- In response to the suicide of a recently graduated student
- If a suicide occurs during the school holidays.

There were also various actions on when a school should organise a parent meeting, which included:

*‘A nominated Emergency Response Team member should organise a meeting open to all parents’*

*‘A nominated Emergency Response Team member should organise a separate meeting for parents of the deceased student’s classmates and close friends’.*

*‘A nominated Emergency Response Team member should organise a separate meeting for parents of students in each year’.*

However, none of these actions were endorsed in the first round (endorsement rates were 45 %, 37.5 % and 20 % respectively). Despite this, we received feedback from panel members that a parent meeting may be appropriate in some circumstances, but were not clear on what those circumstances were. In the second round we added the action ‘*The Emergency Response Team should organise a meeting for parents, which may involve all parents or some parents, based on perceived need’*. This action did receive a higher endorsement than other actions about a parent meeting (69.2 %), however it did not reach a level high enough to be included. In order to reflect the opinion of panel members that a parent meeting may be something a school chooses to organise in some circumstances, we included all endorsed actions about a parent meeting in an appendix to the guidelines. However, the guidelines themselves do not include any action requiring schools to organise a parent meeting.

## Discussion

The current study used the Delphi methodology to create a set of guidelines for schools to use following the suicide of a student. To the authors’ knowledge, this is the first time that a set of postvention guidelines has been produced based on the expert consensus opinion of both researchers and professionals working in the area of suicide postvention, and provides a best practice approach in the context of the current evidence base.

### Comparison with previous literature on postvention actions

The previous literature on postvention responses in schools largely focused upon screening students for suicide risk [2] and on the provision of school-based counselling [15]. For example the study by Poijula et al [2] recommended screening students for symptoms of distress, however this did not reach consensus amongst panel members, being endorsed by just 30 % of people *(‘The Emergency Response Team should implement a systematic screening of in order to identify those at elevated risk of suicide and/or distress. This should cover Psychological distress, Suicidal thoughts and behaviours*’). Panel members commented that the rate of false positives from screening can be high, and schools often do not have the staff resources to screen, issues that have previously been highlighted in the research literature [24]. Furthermore, panel members commented that screening, if it were to be undertaken, would likely be hampered by the need to obtain parental consent, and that resources would be best directed in other areas. However, it should be noted that this action was included within the section on identifying high-risk students, and there was no distinction made between screening which could be implemented immediately following a student suicide, versus that which could be implemented in the months following. Some community response teams (e.g. Riverside Trauma Centre, http://riversidetraumacenter.org), that work with schools have, on a number of occasions, implemented a systematic screening of students around three months following the suicide of a student (personal communication with Jim McCauley, June 19^th^ 2015). Further research may be warranted, in order to determine whether experts in this area consider screening to be of value at different time points, and whether it is something that may be undertaken in schools with an external agency primarily providing the resources and coordination.

With regard to school-based group counselling, this was examined by Hazell and Lewin [15], and was led by an external mental health professional. In the current study, the action ‘*The school wellbeing team should provide group counselling to students who want ongoing support*’ was endorsed by 40 % of panel members, and the action ‘*The Emergency Response Team should arrange for an external service to provide suicide bereavement support groups within the school*’ was endorsed by just 27.5 % of panel members. Panel members commented that support groups being held on school grounds have the potential to detract from the school primarily being a place for learning. However the action ‘*If the demand for counselling exceeds the capacity of the school wellbeing team, the Emergency Response Team should arrange for external counsellors to come to the school for as long as needed*’ was endorsed by 92.3 % of the panel. There appears to be a fine line between communicating to students that support is readily available, whilst maintaining the learning environment. Feedback from panel members also echoed the sentiment that the school staff should deal only with situations that are within their competency and in many cases enlisting the help of external mental health services is likely to be vital in enabling them to provide sufficient support to students.

There were also some interesting differences between previous postvention toolkits, and the recommendations born out of the research. For example, the AFSP contains a section on social media which gives guidance surrounding how long online memorial pages, if set up by the school, should remain active for (stating up to 30 to 60 days). However, panel members within this research did not endorse either of these practices, as shown within the following actions; ‘*If the ER Team establishes an online memorial it should be removed after 1 month*’ (endorsed by 5 %) and; ‘If the ER Team establishes an online memorial it should be removed after 2 months’ (endorsed by 2.5 %). Furthermore, the action ‘The ER Team should set up an online memorial page on a social media site if this is requested by students or staff’, was not endorsed by any panel member, with 45 % choosing the ‘Don’t know/depends’ option, and 55 % choosing the ‘Should not be included in the guidelines’ response. As a result, these guidelines do not include any guidance on setting up online memorials, as this is not a practice endorsed through the Delphi process. Current toolkits can be updated, by looking at the information contained within their own documents, and that produced by this research, and ensuring that their recommendations are consistent.

### Every emergency response team and suicide is unique

One of the main themes that became evident through the Delphi process was the need for schools to be flexible in their response to each student suicide. This was most apparent in the *Funeral and Memorial* section, where panel members were initially asked to rate which types of memorial activities schools should and should not allow (see Additional file 1). None of these actions reached the 80 % consensus level, and feedback from panel members led to the development of the action ‘*The Emergency Response Team should ensure that any memorial sites or activities are the same as they would be for a non-suicide death’.* This was subsequently endorsed by 82.5 % of panel member*s*. Although this guidance may be helpful for schools who have experienced previous deaths, it may be the first time that staff have handled a death and as such, they may require more direct instruction.

We also initially sought to gain consensus with regard to which members of the school community should be responsible for managing high-risk students (see Additional file 1, under *High-risk students* section). However, little consensus was reached around this, and ultimately, feedback from panel members led to the following statement being endorsed ‘*The ER Team should decide who conducts individual suicide risk assessments and develops safety and support plans according to resources and staffing at the school*’. These are just two examples of many that illustrate how a school’s response will likely be influenced by the existing culture, and resources available at the school. Factors such as previous experience, the professional skills of staff members in mental health and suicide risk assessment, and pre-existing relationships with external agencies or services, means that a postvention response will likely vary across schools.

### How to talk about suicide and mental health

In recent years, there has been an increased emphasis on describing suicide using appropriate language that minimises stigma [25]. In the current study, some consensus was reached around the way in which the suicide of a student should be referred to by school staff. In the second consensus round, 92.3 % and 88.5 % of panel members agreed that the guidelines should specifically state that staff should not refer to the person as having ‘*committed suicide*’ or to the death as a ‘*successful suicide*’. This adds to literature which emphasises the need to use language which does not imply that suicide is a crime, or that it is in some way a positive outcome to a situation [26], and provides further guidance for schools on how to talk about suicide in a safe and acceptable manner.

In the section entitled *Informing students* about a suicide, the actions ‘*When teachers are informing students about the death they should tell the students there are treatments available to help with a) mental health problems’ and b) suicidal thoughts*’ both received low rates of consensus (70 and 65 % respectively). Feedback from panel members suggested that the message should be that there is ‘professional help’ available for such difficulties, rather than treatments. In subsequent rounds, the actions ‘*When teachers are informing students about the death they should tell the students there is professional help available for a) mental health problems and b) suicidal thoughts*’ received a much higher level of endorsement, by 92.3 and 84.6 % of panel members respectively. This may reflect the notion that young people tend to seek help from a number of sources, both formal and informal [27], and by communicating that ‘help’ rather than ‘treatments’ are available, this may lessen the stigma associated with seeking help.

### Strengths and limitations of this study

#### Strengths

##### Information used to synthesise these guidelines

The literature search conducted in the first stage of the Delphi process took into account lay literature in addition to the academic literature. Furthermore, inclusion criteria were broad such that study design or quality of specific outcome measures did not preclude documents from being included. As such, the authors were able to obtain a larger volume of information regarding school postvention responses than identified in previous reviews, which have focused solely on the academic literature and have often involved strict inclusion criteria [14, 16, 28]. Many documents retrieved from the academic literature were case studies or descriptions of postvention responses from the perspective of school staff ([29, 30], e.g. [31]). These tended to date back over 20 years, but did contain rich information regarding school responses, as well as activities which in some cases appeared detrimental to the healing of the school community (e.g [32]). The search conducted through google sites and Amazon.com in addition to the academic literature identified a number of existing school postvention resources from a variety of different countries, including Australia, New Zealand, USA and England (AFSP, [11, 12, 33], New Zealand Ministry of Education Professional Practice Unit, [34]). Information regarding how these resources were developed and the evidence upon which they were based was variable. In most cases, it appeared that resources were developed based on a ‘review’ of the literature, however specific details regarding the nature of that literature were absent, making it difficult to determine the basis on which guidance was being formulated. Some resources included a list of academic experts and community members, who were involved in the development of the resource (e.g. AFSP, 2011), and in these cases, it appeared that expert opinion contributed strongly to their formation. However, the method of eliciting expert opinion and obtaining consensus was not made explicit. The current study overcomes these limitations by systematically gathering expert opinion in the area of suicide postvention in schools.

Overall, the literature search conducted in the current study highlighted the lack of evidence regarding the effectiveness of various school postvention approaches. This echoes other research demonstrating the lack of published research into suicide postvention [35] and signals the importance of an agenda to increase research in this area. The need to generate more evidence in this area is especially important given that most national suicide prevention strategies include recommendations for suicide postvention and crisis intervention services (WHO, [1]). It is therefore imperative that the recommendations within such documents are developed from the best available evidence, so that subsequent service delivery is based on best practice in the field. As the literature search indicated that postvention responses within school communities are widespread, there are ample opportunities for research to be conducted, in order to evaluate the effectiveness of postvention responses and increase the evidence base in school suicide postvention specifically.

### Diversity of experience

The panel members who participated in this research have been involved in various aspects of suicide postvention in schools. There was a mixture of school counsellors, psychologists and wellbeing staff, as well as principals, departmental psychologists, and independent mental health professionals who have assisted schools following the suicide of a student. In addition, academic researchers who had contributed to other school postvention guidelines and/or published in the broader area of suicide postvention were also panel members. As such, their collective opinion is a reflection of what is likely to be feasible in schools, given the likely constraints on resources, as well as what is likely to be ‘best practice’ in this area given the currently available evidence. Diversity of expertise is known to produce better quality judgments [36].

### Limitations

Whilst the search method employed in the first stage of this research was broad and inclusive of a variety of literature, there is the possibility that some postvention actions taken by schools may have been missed due to this information not being freely available in the public domain. However, it should also be noted that 3949 potential actions were extracted from the documents located and panel members were also able to suggest additional actions for consideration. It would be expected that if actions had been missed, they were not critical to a postvention response, and therefore were unlikely to have been endorsed.

As fourteen panel members dropped out after the first consensus round, in the second round, consensus rates in the second round were based on fewer expert opinions. However, previous research by Akins et al [37] found that a panel of 23 experts is large enough to produce stable results over time. Given that all rounds contained more than 23 expert panel members, it is likely that the results produced are a robust and fair representation of opinion.

This study was conducted largely within an Australian setting, with the aim of producing guidelines applicable to English-speaking western schools. As such, one of the main limitations of these guidelines is their limited applicability to schools in other settings, and in particular to those that have a large indigenous population. Very few panel members worked in Australian schools in a rural location, within a largely Aboriginal community. One panel member commented ‘*I’m not sure how to address all the issues from an indigenous perspective, but in the community (2000 people) and school (800 enrolled T-12) that I work/live in then this [what] is the most significant issue and these guidelines need to sit behind them and we proceed based on what our cultural adviser directs us to do’*. Given this feedback, it is interesting to note that the statement ‘*The ERT should contain a representative from any major cultural groups*’ did not reach consensus, being endorsed by just 57.7 % of all participants. The importance of cultural representation appears to be very much determined by the demographics of the school community being served and signals that aspects of these guidelines will need to be adapted for schools which have a predominantly indigenous population. It is also interesting to note that throughout the literature search, there were few documents retrieved which specifically focused on responding to suicide within a school with a large indigenous population. This echoes the broader suicide prevention literature, where there is a lack of research evaluating the effectiveness of interventions for this group, despite high suicide rates [38, 39].

### Future research

The development of these guidelines signals a number of avenues for future research. Firstly, although these guidelines contain the opinions of experts in the area of suicide postvention, the voices of young people who have experienced the suicide of a peer are absent. We did consider recruiting young people with this experience as panel members, however there were many organisational actions which students would likely not be involved in, and hence would not have expert knowledge on (e.g. having a staff meeting, liaising with outside agencies). Qualitative research with young people who have experienced the death of a peer thus far has focused on their overall experience of that loss [7], and has shown that asking young people about their experience of suicide can be done in a safe way within the context of a research study. One research study has focused on how the school can be most helpful following a suicide from the perspective of young people [40], however only a small minority of young people who participated had lost either a close relative or a friend to suicide (11 %). Future research, potentially using focus groups or in-depth qualitative interviews, could explore how young people feel about the way their school handled the suicide of a peer and what actions were most helpful in helping the student community come to terms with such a distressing event.

Given the feedback from panel members regarding the applicability of these guidelines in school communities with a predominantly Aboriginal or indigenous population, there is the opportunity to compare and contrast exactly which aspects of these guidelines will need to be adapted for these schools, in both Australia, and in other countries such as New Zealand.

Any guidelines produced should be implemented within the context of an evaluation framework. In the future, qualitative research should be pursued with schools that use these guidelines as a template to respond to a student suicide in order to yield information on both the feasibility and utility of such guidelines.

## Conclusion

These guidelines are the first internationally that have used the Delphi methodology to reach expert consensus with regard to the actions schools should take following the suicide of a student. It is hoped that they will be used by schools in order to either aid them in developing a plan to respond to a student suicide or to respond to a suicide in the absence of a predetermined plan. They can also be used by the wider postvention community to inform already existing postvention resources (e.g. AFSP, [11, 12]), as well as being used to develop new postvention resources for schools. They also further the evidence base in an extremely under researched area, by using a consensus methodology based upon the ‘wisdom of crowds’ approach [21].

### Availability of data and materials

The datasets supporting the conclusions of this article are included within the article and its additional files.

## Additional file

Additional file 1:
**List of actions rated by panel members.** (PDF 1 mb)
