# Supplementary material for: Development of suicide postvention guidelines for secondary schools: a Delphi study
Source: BMC Public Health. 2016 Feb 24;16:180. doi: 10.1186/s12889-016-2822-6 (PMC4765027; doi:10.1186/s12889-016-2822-6)
Supplement: Additional file 1: — List of actions rated by panel members. (PDF 1 mb) [file 12889_2016_2822_MOESM1_ESM.pdf]

Additional file 1: List of actions rated by panel members

	Included in guidelines	Round	% of panel members rated as 'Essential' or 'Important'	% of panel members rated as 'Unimportant'	% of panel members rated as 'Don't know/ depends'	% of panel members rated as 'Should not be included'	TOTAL
Emergency Response (ER) Plan and Emergency Response (ER Team)							
The school should have a written ER Plan in place prior to a suicide event.	Yes	1	100.0	0.0	0.0	0.0	100.0
The ER Plan should include a resource kit for the ER Team to use following a death, which includes emergency telephone numbers of all ER Team members.	Yes	1	100.0	0.0	0.0	0.0	100.0
The ER Plan should include a resource kit for the ER Team to use following a death, which includes contact details of external mental health professionals/services.	Yes	1	100.0	0.0	0.0	0.0	100.0
The ER Plan should include a resource kit for the ER Team to use following a death, which includes contact details of key people in the local community (e.g. parent groups, clergy).	Yes	1	100.0	0.0	0.0	0.0	100.0
The ER Team should have a designated leader.	Yes	1	100.0	0.0	0.0	0.0	100.0
The leader of the ER Team should be someone who is able to provide a calm, controlled and reassuring presence.	Yes	1	100.0	0.0	0.0	0.0	100.0

The leader of the ER Team should be someone who is able to communicate effectively with many different groups (e.g. students, staff, parents, community members).	Yes	1	100.0	0.0	0.0	0.0	100.0
Members of the ER Team should be chosen so that the team has a combination of knowledge of how the school and community functions.	Yes	1	100.0	0.0	0.0	0.0	100.0
The leader of the ER Team should receive training in dealing with suicide and its aftermath.	Yes	1	100.0	0.0	0.0	0.0	100.0
The principal should be aware of the roles and responsibilities of each member of the ER Team and the impact this may have on their workload.	Yes	2	100.0	0.0	0.0	0.0	100.0
The leader of the ER Team should have an in-depth knowledge of the ER Plan and all of the responsibilities contained within it.	Yes	2	100.0	0.0	0.0	0.0	100.0
A nominated ER Team member should ensure that any pre-prepared templates, letters, and handouts are suitable given the context and nature of the suicide.	Yes	2	100.0	0.0	0.0	0.0	100.0
The school should form an ER Team prior to a suicide event, whose responsibility it is to implement the ER Plan.	Yes	1	97.5	0.0	0.0	2.5	100.0
The ER Plan should include instructions for contacting all school staff as soon as possible following the suicide. These may vary if the suicide occurs inside or outside of school hours, at the weekend, or in the school holidays.	Yes	1	97.5	0.0	2.5	0.0	100.0
The ER Team should review the ER Plan annually to update roles and contact details.	Yes	1	97.5	0.0	2.5	0.0	100.0
The leader of the ER Team should be someone who is able to encourage and support staff.	Yes	1	97.5	2.5	0.0	0.0	100.0

A member of the school wellbeing team should be a member of the school wellbeing team.	Yes	1	97.5	0.0	2.5	0.0	100.0
All members of the ER Team should have a checklist detailing the tasks that they should complete following a suspected or confirmed suicide.	Yes	1	97.5	0.0	2.5	0.0	100.0
An overview of the ER Plan should be disseminated to all school staff.	Yes	2	96.2	0.0	0.0	3.8	100.0
The leader of the ER Team should be someone who is a recognised and authoritative leader, who has shown the ability to lead in a crisis.	Yes	2	96.2	0.0	3.8	0.0	100.0
The ER Team members should channel all information through the nominated leader.	Yes	2	96.2	0.0	3.8	0.0	100.0
The ER Plan should specify how space will be allocated for any support rooms, individual counselling, or external mental health professionals.	Yes	1	95.0	5.0	0.0	0.0	100.0
The leader of the ER Team should give direction to the school staff and any outside agencies.	Yes	1	95.0	0.0	5.0	0.0	100.0
Members of the ER Team should be chosen so that the team has a combination of experience with crisis intervention and management procedures.	Yes	1	95.0	2.5	2.5	0.0	100.0
All members of the ER Team should receive training in dealing with suicide and its aftermath.	Yes	1	95.0	5.0	0.0	0.0	100.0
The ER Team should develop relationships with relevant local services (e.g. youth mental health services, police, media).	Yes	1	95.0	2.5	2.5	0.0	100.0
A member of the school wellbeing team should be involved in the development of the ER Plan.	Yes	1	92.5	2.5	5.0	0.0	100.0
The ER Plan should include a chart that sequentially outlines the initial crisis response.	Yes	1	92.5	5.0	2.5	0.0	100.0

The ER Plan should include a resource kit for the ER Team to use following a death, which includes emergency telephone numbers of all staff.	Yes	1	92.5	5.0	0.0	2.5	100.0
The leader of the ER Team should coordinate all other members of the team and ensure that they stick to their delegated roles.	Yes	1	92.5	2.5	5.0	0.0	100.0
The ER Team should designate someone to take over the role of leader if the appointed person is absent.	Yes	1	92.5	0.0	5.0	2.5	100.0
The school principal should be a member of the school wellbeing team.	Yes	1	92.5	0.0	7.5	0.0	100.0
A nominated ER Team member should prepare scripts that may be used to inform the school community about the death and the school's response.	Yes	1	92.5	2.5	2.5	2.5	100.0
The principal should decide who will take over if the designated leader is absent, with advice from the ER Team if necessary.	Yes	2	92.3	0.0	7.7	0.0	100.0
The ER Team should remind all staff of the ER Plan at the beginning of each school year.	Yes	1	90.0	2.5	5.0	2.5	100.0
The leader of the ER Team should be someone who is knowledgeable about youth suicide.	Yes	1	90.0	5.0	5.0	0.0	100.0
A nominated ER Team member should prepare any written information (e.g. fact sheets, handouts) to be given to the school community.	Yes	1	90.0	0.0	7.5	2.5	100.0
The ER Plan should include provisions for adequate staffing to cover for team members who are unable to fulfil their roles (e.g. sick, away, or otherwise unable to participate).	Yes	2	88.5	0.0	11.5	0.0	100.0
Each task specified in the ER Plan should have a named individual who is responsible for its implementation.	Yes	1	87.5	2.5	7.5	2.5	100.0

The ER Plan should include a resource kit for the ER Team to use following a death, which includes an up-to-date list of students enrolled in school.	Yes	1	87.5	7.5	5.0	0.0	100.0
The ER Plan should make provisions for providing support for students in the holidays when a death by suicide occurs outside the school year.	Yes	1	87.5	2.5	7.5	2.5	100.0
Members of the ER Team should be chosen so that the team has a combination of strong individual and group facilitation skills.	Yes	1	87.5	10.0	2.5	0.0	100.0
The leader of the ER Team should be someone who is able to provide much positive reinforcement.	Yes	1	85.0	5.0	7.5	2.5	100.0
At least one member of the ER Team should be trained in performing suicide risk assessments, using a validated tool that can be made available during following a suicide.	Yes	1	85.0	2.5	7.5	5.0	100.0
There should be an assistant leader who carries out the day-to-day tasks of the ER Plan that the nominated leader does not have time to undertake.	Yes	2	84.6	7.7	3.8	3.8	100.0
The principal should ensure there is a budget to implement the ER Plan (e.g. training, external support services).	Yes	1	82.5	5.0	10.0	2.5	100.0
The ER Plan should make provisions for handling the death of a recently graduated student.	Yes	1	82.5	0.0	17.5	0.0	100.0
The ER Plan should include a resource kit for the ER Team to use following a death, which includes a school map with the location of school telephones, designated meeting rooms, and any student support room.	Yes	2	80.8	11.5	7.7	0.0	100.0
The ER Plan should include a resource kit for the ER Team to use following a death, which includes an up-to-date schedule of classes.	Yes	1	80.0	12.5	7.5	0.0	100.0

The assistant principal should be a member of the school wellbeing team.	Yes	1	80.0	2.5	17.5	0.0	100.0
The ER Team should be trained and have practice runs in implementing the ER Plan.	Yes	1	80.0	2.5	15.0	2.5	100.0
The ER Plan should be disseminated to all school staff.	Re-rated in round 2	1	77.5	5.0	7.5	10.0	100.0
A representative from the relevant education department should be a member of the ER Team.	Re-rated in round 3	2	76.9	3.8	15.4	3.8	100.0
The leader of the ER Team should be the principal.	Re-rated in round 2	1	75.0	2.5	22.5	0.0	100.0
The ER Plan should include a resource kit for the ER Team to use following a death, which includes a school map with the location of school telephones, designated meeting rooms, and any student support room.	Re-rated in round 2	1	70.0	22.5	7.5	0.0	100.0
The leader of the ER Team should introduce themselves to the local police and coroner as part of their planning.	Re-rated in round 2	1	70.0	12.5	12.5	5.0	100.0
The leader of the ER Team should be the principal.	No	2	65.4	11.5	23.1	0.0	100.0
The local suicide prevention coordinator (where one exists) should be a member of the ER Team.	No	2	65.4	7.7	19.2	7.7	100.0
The leader of the ER Team should introduce themselves to the local police as part of their planning.	No	2	65.4	7.7	19.2	7.7	100.0
The school nurse should be a member of the school wellbeing team.	No	1	65.0	5.0	30.0	0.0	100.0
The school board/council should approve the ER Plan.	No	1	62.5	12.5	15.0	10.0	100.0

An administrator should be a member of the school wellbeing team.	No	1	62.5	10.0	20.0	7.5	100.0
IT or computer staff (e.g. to deal with social media) should be a member of the school wellbeing team.	No	1	62.5	7.5	25.0	5.0	100.0
A mental health professional from an external service should be a member of the school wellbeing team.	No	1	62.5	12.5	22.5	2.5	100.0
The ER Plan should be disseminated to all school staff.	No	2	61.5	19.2	3.8	15.4	100.0
The ER Team should remind all staff of the ER Plan at the beginning of each school term.	No	2	61.5	15.4	19.2	3.8	100.0
The leader of the ER Team should be a member of the school wellbeing team.	No	1	60.0	2.5	22.5	15.0	100.0
A representative from any major cultural groups should be a member of the ER Team.	No	2	57.7	11.5	26.9	3.8	100.0
The school should get legal advice in developing the ER Plan.	No	1	52.5	22.5	15.0	10.0	100.0
The ER Plan should include a resource kit for the ER Team to use following a death, which includes name tags for ER Team members.	No	1	50.0	35.0	12.5	2.5	100.0
A representative from the relevant education department should be a member of the ER Team	No	3	48.0	12.0	32.0	8.0	100.0
An overview of the ER Plan should be disseminated to parents of all students at the school.	No	2	46.2	15.4	11.5	26.9	100.0
The ER Plan should include a resource kit for the ER Team to use following a death, which includes keys to all the doors of the school.	No	1	45.0	30.0	20.0	5.0	100.0
The leader of the ER Team should be a mental health professional.	No	1	42.5	12.5	25.0	20.0	100.0
The leader of the ER Team should introduce themselves to the local police and coroner as part of their planning.	No	2	42.3	15.4	26.9	15.4	100.0

The chaplain/minister of religion should be a member of the school wellbeing team.	No	1	40.0	15.0	42.5	2.5	100.0
A member of the local police should be a member of the school wellbeing team.	No	1	40.0	7.5	35.0	17.5	100.0
A parent representative should be a member of the school wellbeing team.	No	1	30.0	7.5	20.0	42.5	100.0
A representative from the local child welfare services should be a member of the ER Team.	No	2	26.9	23.1	34.6	15.4	100.0
The leader of the ER Team should be a school nurse.	No	1	22.5	15.0	42.5	20.0	100.0
A student representative should be a member of the school wellbeing team.	No	1	17.5	10.0	30.0	42.5	100.0
A school board/council representative should be a member of the school wellbeing team.	No	1	17.5	20.0	45.0	17.5	100.0
The ER Plan should be disseminated to parents of all students at the school.	No	2	7.7	7.7	15.4	69.2	100.0
A cleaner/caretaker should be a member of the school wellbeing team.	No	1	5.0	32.5	27.5	35.0	100.0
Managing a suspected suicide that occurs on school grounds							
If the death of a student has occurred on school grounds, staff should alert a member of the ER Team.	Yes	1	100.0	0.0	0.0	0.0	100.0
If the death of a student has occurred on school grounds, staff should isolate the site of the death from students and unauthorised staff access by using screens, blocking corridors and evacuating the area.	Yes	1	100.0	0.0	0.0	0.0	100.0
If any student witnessed the death, a nominated ER Team member should arrange for supervision and support by a member of staff.	Yes	1	100.0	0.0	0.0	0.0	100.0

If the death of a student has occurred on school grounds, staff should ensure that no-one removes or disturbs items from the site until police have concluded their work.	Yes	1	97.5	0.0	2.5	0.0	100.0
If any student witnessed the death, a nominated ER Team member should contact their parent/guardian and inform them of the situation.	Yes	1	97.5	0.0	2.5	0.0	100.0
If any student witnessed the death, a nominated ER Team member should offer them support from a mental health professional.	Yes	1	97.5	0.0	2.5	0.0	100.0
If any staff member witnessed the death, a nominated ER Team member should offer them support from a mental health professional.	Yes	1	97.5	0.0	2.5	0.0	100.0
If the death of a student has occurred on school grounds, staff should follow existing school protocols for managing any death.	Yes	1	95.0	0.0	2.5	2.5	100.0
If any staff member witnessed the death, a nominated ER Team member should arrange for support by another member of staff.	Yes	1	92.5	0.0	7.5	0.0	100.0
If any staff member witnessed the death, a nominated ER Team member should offer them relief from their duties for the day.	Yes	1	90.0	2.5	7.5	0.0	100.0
If any student witnessed the death, a nominated ER Team member should give the student the option of being collected by a parent/guardian.	Yes	1	85.0	0.0	7.5	7.5	100.0
As far as possible, the school should keep running as usual.	No	1	65.0	0.0	30.0	5.0	100.0
The ER Team should consider closing the school if the death of a student has occurred on school grounds during the day and there is risk of significant distress or disruption.	No	1	37.5	2.5	42.5	17.5	100.0

Confirming facts							
If the death of a student has occurred on school grounds, staff should alert the principal.	Yes	2	100.0	0.0	0.0	0.0	100.0
A nominated ER Team member should verify reports of suicide that do not come from the immediate family by contacting the police, coroner, hospital staff or the family.	Yes	1	95.0	0.0	2.5	2.5	100.0
If a staff member witnessed the death and they are relieved from their duties for the day, a member of the ER Team should check whether support is available to them outside of the school.	Yes	2	92.3	0.0	7.7	0.0	100.0
When talking about the death, staff should not use the term “Committed suicide”.	Yes	2	92.3	0.0	0.0	7.7	100.0
A nominated ER Team member should obtain information about whether any students or staff from the school witnessed the suspected suicide, and who they were, as soon as possible.	Yes	2	92.3	0.0	7.7	0.0	100.0
When talking about the death, staff should not use the term "Successful suicide".	Yes	2	88.5	0.0	0.0	11.5	100.0
A nominated ER Team member should check with the police whether any students will need to be interviewed about the death.	Yes	2	84.6	3.8	11.5	0.0	100.0
A nominated ER Team member should obtain information about whether there were any witnesses and who they were to a suspected suicide as soon as possible.	Yes	1	82.5	0.0	15.0	2.5	100.0
If the death is known to be a suicide, and the family gives permission, the death should be referred to as a “suicide”, unless advised otherwise by mental health professionals because of the risk of contagion.	Re-rated in round 2	1	77.5	0.0	5.0	17.5	100.0

When talking about the death, staff should not use the term "Successful suicide".	Re-rated in round 2	1	77.5	0.0	5.0	17.5	100.0
A nominated ER Team member should obtain information about the location of a suspected suicide as soon as possible.	No	2	76.9	3.8	19.2	0.0	100.0
A nominated ER Team member should obtain information about the location of a suspected suicide as soon as possible.	Re-rated in round 2	1	75.0	10.0	5.0	10.0	100.0
A nominated ER Team member should check with the police whether any students will need to be interviewed about the death.	Re-rated in round 2	1	75.0	7.5	12.5	5.0	100.0
A nominated ER Team member should verify reports of suicide that do come from the immediate family by contacting the police, coroner or hospital staff.	Re-rated in round 3	2	73.1	11.5	11.5	3.8	100.0
A nominated ER Team member should obtain information about the time of a suspected suicide as soon as possible.	No	2	73.1	7.7	19.2	0.0	100.0
When talking about the death, staff should not use the term “Committed suicide”.	Re-rated in round 2	1	72.5	2.5	12.5	12.5	100.0
A nominated ER Team member should obtain information about the time of a suspected suicide as soon as possible.	Re-rated in round 2	1	70.0	12.5	10.0	7.5	100.0
When talking about the death, staff should use the term “Died by suicide”.	Re-rated in round 2	1	70.0	0.0	12.5	17.5	100.0
If the death is known to be a suicide, and the family gives permission, school staff should refer to the death as a “suicide”, unless advised otherwise by mental health professionals because of the risk of contagion.	No	2	69.2	0.0	11.5	19.2	100.0

If the death is not being referred to as a suicide by the students and the family objects to the death being labelled as a "suicide", school staff should refer to the death as a "tragic death".	No	2	69.2	0.0	7.7	23.1	100.0
A nominated ER Team member should verify reports of suicide that do not come from the immediate family by contacting the relevant education department.	No	2	69.2	3.8	7.7	19.2	100.0
If the death is being referred to as a suicide by the students and the family objects to the death being labelled as a "suicide", school staff should refer to the death as a "tragic death".	No	2	65.4	0.0	7.7	26.9	100.0
When talking about the death, staff should not use the term "Completed suicide".	No	1	65.0	2.5	7.5	25.0	100.0
When talking about the death, staff should use the term "Took their own life".	No	1	62.5	5.0	15.0	17.5	100.0
If a coroner has ruled that the death is a suicide and the family objects to the label, the death should be referred to as a sudden death.	No	1	55.0	2.5	27.5	15.0	100.0
Throughout the guidelines the death should be referred to as a "death".	No	2	53.8	7.7	26.9	11.5	100.0
A nominated ER Team member should obtain information about the method of a suspected suicide as soon as possible.	No	1	50.0	22.5	10.0	17.5	100.0
If the death is known to be a suicide, and the family does not want it to be labelled as such, school staff should state that "the family do not want any reference made to the cause of death".	No	2	50.0	0.0	26.9	23.1	100.0

Before the death is officially classified as a suicide by the coroner's office, the death should be referred to as a “Suicide” if this is consistent with the family's wishes.	No	1	47.5	0.0	30.0	22.5	100.0
Throughout the guidelines the death should be referred to as a "suicide".	No	2	46.2	0.0	23.1	30.8	100.0
Before the death is officially classified as a suicide by the coroner's office, the death should be referred to as a tragic loss.	No	1	42.5	0.0	17.5	40.0	100.0
When talking about the death, staff should use the term “Died by suicide”.	No	2	42.3	3.8	26.9	26.9	100.0
Before the death is officially classified as a suicide by the coroner's office, the death should be referred to as a sudden death with the cause unknown.	No	1	40.0	2.5	17.5	40.0	100.0
If a coroner has ruled that the death is a suicide and the family objects to the label, the death should be referred to as a tragic loss.	No	1	40.0	2.5	30.0	27.5	100.0
If the death is not being referred to as a suicide by the students and the family objects to the death being labelled as a "suicide", school staff should refer to the death as a "traumatic death".	No	2	38.5	3.8	11.5	46.2	100.0
A nominated ER Team member should obtain information about whether there was a suicide note and its contents related to a suspected suicide as soon as possible.	No	1	37.5	17.5	17.5	27.5	100.0
Before the death is officially classified as a suicide by the coroner's office, the death should be referred to as a suspected suicide.	No	1	37.5	2.5	20.0	40.0	100.0

If the death is being referred to as a suicide by the students and the family objects to the death being labelled as a "suicide", school staff should refer to the death as a "traumatic death".	No	2	34.6	3.8	15.4	46.2	100.0
If the death is known to be a suicide, and the family gives permission, school staff should refer to the death as a "possible suicide".	No	2	30.8	3.8	15.4	50.0	100.0
Throughout the guidelines the death should be referred to as a "suspected suicide".	No	2	30.8	3.8	19.2	46.2	100.0
If a coroner has ruled that the death is a suicide and the family objects to the label, the death should be referred to as a "Suicide ruled by the coroner", if asked directly by students.	No	1	30.0	2.5	42.5	25.0	100.0
When talking about the death, staff should not use the term “Died by suicide”.	No	1	27.5	10.0	22.5	40.0	100.0
If the death is known to be a suicide, and the family gives permission, school staff should refer to the death as a "tragic death".	No	2	26.9	3.8	30.8	38.5	100.0
If the death is being referred to as a suicide by the students and the family objects to the death being labelled as a "suicide", school staff should refer to the death as a "possible suicide".	No	2	26.9	0.0	19.2	53.8	100.0
Throughout the guidelines the death should be referred to as a "possible suicide".	No	2	26.9	3.8	23.1	46.2	100.0
If the death is known to be a suicide, and the family gives permission, school staff should refer to the death as a "suspected suicide".	No	2	23.1	3.8	15.4	57.7	100.0
Throughout the guidelines the death should be referred to as a "tragic death".	No	2	19.2	7.7	23.1	50.0	100.0

When talking about the death, staff should not use the term "Took their own life".	No	1	17.5	15.0	27.5	40.0	100.0
If the death is known to be a suicide, and the family gives permission, school staff should refer to the death as a "traumatic death".	No	2	15.4	3.8	26.9	53.8	100.0
If the death is being referred to as a suicide by the students and the family objects to the death being labelled as a "suicide", school staff should refer to the death as a "suspected suicide".	No	2	15.4	0.0	23.1	61.5	100.0
When talking about the death, staff should use the term "Completed suicide".	No	1	12.5	0.0	10.0	77.5	100.0
Throughout the guidelines the death should be referred to as a "student who passed away suddenly".	No	2	11.5	3.8	15.4	69.2	100.0
Do not contact the family to verify reports of a suspected suicide.	No	2	11.5	0.0	42.3	46.2	100.0
When talking about the death, staff should use the term “Committed suicide”.	No	1	10.0	2.5	7.5	80.0	100.0
If the death is being referred to as a suicide by the students and the family objects to the death being labelled as a "suicide", school staff should refer to the death as a “suicide”, unless advised otherwise by mental health professionals because of the risk of contagion.	No	2	7.7	0.0	23.1	69.2	100.0
Throughout the guidelines the death should be referred to as a "traumatic death".	No	2	7.7	7.7	19.2	65.4	100.0
When talking about the death, staff should use the term "Successful suicide".	No	1	5.0	0.0	5.0	90.0	100.0

If the death is not being referred to as a suicide by the students and the family objects to the death being labelled as a "suicide", school staff should refer to the death as a "possible suicide".	No	2	3.8	0.0	15.4	80.8	100.0
If the death is not being referred to as a suicide by the students and the family objects to the death being labelled as a "suicide", school staff should refer to the death as a “suicide”, unless advised otherwise by mental health professionals because of the risk of contagion.	No	2	0.0	0.0	15.4	84.6	100.0
If the death is not being referred to as a suicide by the students and the family objects to the death being labelled as a "suicide", school staff should refer to the death as a "suspected suicide".	No	2	0.0	0.0	11.5	88.5	100.0
Activating the ER Team							
After a death is verified, the ER Team should meet and implement the ER Plan.	Yes	1	97.5	0.0	0.0	2.5	100.0
The ER Team should meet even if not all members are present.	Yes	1	97.5	0.0	2.5	0.0	100.0
The ER Team meetings should include discussions about progress of the implementation of the ER Plan.	Yes	1	97.5	0.0	0.0	2.5	100.0
If the principal is not a member of the ER Team, a nominated team member should keep them informed of the school's response to the death.	Yes	1	97.5	0.0	0.0	2.5	100.0
If the death occurred during the school holidays, the school should acknowledge the death as soon as possible and follow the ER Plan as appropriate.	Yes	1	97.5	0.0	2.5	0.0	100.0
The first ER Team member to become aware of a suspected suicide should alert the leader of the ER Team that a death has occurred.	Yes	2	96.2	0.0	3.8	0.0	100.0

The ER Team should nominate a member to coordinate all ER Team and staff meetings.	Yes	1	92.5	0.0	5.0	2.5	100.0
The ER Team should assess the likely level of impact on the school community and tailor the level of response accordingly.	Yes	1	92.5	0.0	2.5	5.0	100.0
The ER Plan should be implemented in response to a suspected or confirmed suicide of a student who is enrolled at the school but is currently not attending (e.g. on exchange, on extended sick leave).	Yes	3	92.0	0.0	8.0	0.0	100.0
The ER Plan should be implemented in response to a suspected or confirmed suicide of a current student.	Yes	1	90.0	2.5	2.5	5.0	100.0
The ER Team should meet within 24 hours.	Yes	1	90.0	2.5	5.0	2.5	100.0
The designated leader should keep all ER Team members informed of the ER Team's work in the daily meetings.	Yes	1	90.0	2.5	5.0	2.5	100.0
The ER Team meetings should include discussions about high-risk students.	Yes	1	90.0	0.0	7.5	2.5	100.0
In the initial ER Team meeting, the leader of the ER Team should delegate all of the relevant tasks that cannot be delegated in advance.	Yes	2	88.5	0.0	11.5	0.0	100.0
In the initial ER Team meeting, the leader of the ER Team should delegate all of the relevant tasks.	Yes	1	85.0	2.5	10.0	2.5	100.0
The ER Team should meet daily for as long as deemed necessary.	Yes	2	84.6	0.0	7.7	7.7	100.0
The ER Team meetings should include discussions about any new resources (e.g. Fact sheets, handouts) not already contained in the ER Plan.	Yes	3	84.0	4.0	4.0	8.0	100.0

The leader of the ER Team should contact the ER Team members and inform them that a death has occurred, only after confirming this with the school principal (if not the leader).	Yes	2	80.8	0.0	19.2	0.0	100.0
If the death occurred during the school holidays, the ER Team should be guided by any local external response that is occurring.	Yes	2	80.8	0.0	15.4	3.8	100.0
A nominated ER Team member should verify reports of suicide that do come from the immediate family by contacting the police, coroner or hospital staff.	Yes	3	80.0	0.0	4.0	16.0	100.0
The first ER Team member to become aware of a suspected suicide should alert all other members of the ER Team that a death has occurred.	Re-rated in round 2	1	77.5	2.5	10.0	10.0	100.0
The ER Plan should be implemented in response to a suspected or confirmed suicide of a student who is enrolled at the school but is currently not attending (e.g. on exchange, on extended sick leave).	Re-rated in round 3	2	76.9	3.8	19.2	0.0	100.0
The ER Team meetings should include discussions about any new resources (e.g. Factsheets, handouts) not already contained in the ER Plan.	Re-rated in round 3	2	76.9	7.7	11.5	3.8	100.0
If the death occurred off school grounds, the ER Team should not close the school.	Re-rated in round 2	1	72.5	0.0	22.5	5.0	100.0
If the death occurred off school grounds, the ER Team should not close the school.	No	2	69.2	0.0	26.9	3.8	100.0
The ER Plan should be implemented in response to a suspected or confirmed suicide of a student who has recently left the school.	No	2	69.2	0.0	30.8	0.0	100.0

The ER Team should meet both at the start of each day and at the end of each day for as long as necessary.	No	2	69.2	0.0	19.2	11.5	100.0
The ER Team should meet every day for the first week.	No	1	67.5	5.0	27.5	0.0	100.0
The leader of the ER Team should contact the ER Team members and inform them that a death has occurred.	No	2	61.5	0.0	11.5	26.9	100.0
The designated leader of the ER Team should ensure that the workload is evenly shared amongst members of the team.	No	1	52.5	20.0	22.5	5.0	100.0
The ER Plan should be implemented in response to a suspected or confirmed suicide of a student's sibling, where the sibling does not attend the school.	No	2	42.3	0.0	53.8	3.8	100.0
The first ER Team member to become aware of a suspected suicide should alert all other members of the ER Team that a death has occurred.	No	2	26.9	3.8	26.9	42.3	100.0
The school should not implement the ER Plan if it is unlikely that students will become aware of the suicide (e.g. the death is of an ex-student).	No	1	17.5	0.0	45.0	37.5	100.0
Liaising with the deceased student's family							
A nominated ER Team member should liaise with the family to offer the condolences of the school.	Yes	1	100.0	0.0	0.0	0.0	100.0
When communicating with the family, a nominated ER Team member should consider the family's cultural and/or religious practices regarding death and suicide.	Yes	1	97.5	0.0	2.5	0.0	100.0
A nominated ER Team member should advise family members about the availability of grief counselling, external mental health services and survivor support groups that can offer help.	Yes	1	97.5	0.0	2.5	0.0	100.0
The ER Team should respect the wishes of the family in regard to privacy and confidentiality as far as	Yes	2	96.2	0.0	3.8	0.0	100.0

possible whilst maintaining the wellbeing of the school community.							
A nominated ER Team member should liaise with the family to discuss and arrange support of siblings if they also attend the school.	Yes	1	95.0	0.0	5.0	0.0	100.0
A nominated ER Team member should liaise with the family to determine the family's wishes on the school's representation at the funeral/service.	Yes	1	92.5	0.0	7.5	0.0	100.0
A nominated ER Team member should liaise with the family to seek permission to name the deceased student in communication with the school community.	Yes	1	92.5	0.0	5.0	2.5	100.0
A nominated ER Team member should liaise with the family to give information about the school's response plan.	Yes	1	85.0	2.5	10.0	2.5	100.0
A nominated ER Team member should liaise with the family to alert the family of potential or advised media contact.	Yes	1	85.0	0.0	12.5	2.5	100.0
A nominated ER Team member should liaise with the family to identify close friends and extended family members who attend the school and may need additional support.	Yes	1	85.0	0.0	10.0	5.0	100.0
In discussing with the family their wishes about what others should be told, A nominated ER Team member should advise them to consider the damaging impact of misinformation.	Yes	1	85.0	0.0	7.5	7.5	100.0
In discussing with the family their wishes about what others should be told, A nominated ER Team member should advise them to consider that other parents need to know, so they can support their own child's grief.	Yes	1	85.0	2.5	5.0	7.5	100.0

A nominated ER Team member should liaise with the family to discuss possible plans for a memorial service or event.	Yes	2	84.6	0.0	15.4	0.0	100.0
In discussing with the family their wishes about what others should be told, a nominated ER Team member should advise them to consider the recommendations of these guidelines.	Yes	2	84.6	0.0	15.4	0.0	100.0
The ER Team should respect the wishes of the family in regard to privacy and confidentiality.	Yes	1	82.5	0.0	15.0	2.5	100.0
If it is difficult to speak directly with the immediate family, a nominated ER Team member should liaise with an extended family member or close friend.	Yes	1	82.5	0.0	7.5	10.0	100.0
Before contacting the family, the nominated ER Team member should check for any recent history/incidents involving the deceased student that may impact on the communication with the family (e.g. bullying).	Yes	2	80.8	11.5	7.7	0.0	100.0
In discussing with the family their wishes about what others should be told, a nominated ER Team member should advise them to consider the benefits of being able to discuss suicide openly where appropriate.	Yes	2	80.8	3.8	7.7	7.7	100.0
A nominated ER Team member should liaise with the family to seek permission to label the death as a suicide.	Yes	1	80.0	0.0	10.0	10.0	100.0
The principal should contact the family by phone within 24 hours.	No	2	76.9	3.8	15.4	3.8	100.0
A nominated ER Team member should liaise with the family to discuss possible plans for a memorial service or event.	Re-rated in round 2	1	75.0	0.0	12.5	12.5	100.0
In discussing with the family their wishes about what others should be told, A nominated ER Team member	Re-rated in round 2	1	75.0	10.0	12.5	2.5	100.0

should advise them to consider the recommendations of these guidelines.							
When visiting the family, the nominated ER Team member should seek assistance from a staff member who knew the deceased student.	No	2	73.1	3.8	19.2	3.8	100.0
When visiting the family, the nominated ER Team member should seek assistance from a staff member who knew the deceased student.	Re-rated in round 2	1	70.0	2.5	15.0	12.5	100.0
A nominated ER Team member should make a home visit to the family, if deemed appropriate and if the family consents.	No	2	69.2	3.8	26.9	0.0	100.0
The principal should contact the family of the deceased student by phone within 24 hours.	No	3	68.0	0.0	28.0	4.0	100.0
If the family do not wish the death to be referred to as a suicide, a nominated ER Team member should keep them informed of any discussion between students that the death was a suicide, as this may change their mind.	No	1	50.0	10.0	15.0	25.0	100.0
A nominated ER Team member should contact the family by phone within 24 hours.	No	2	42.3	0.0	30.8	26.9	100.0
The ER Team should respect the wishes of the family in regard to privacy and confidentiality when the level of risk to the school community outweighs respecting the family's wishes.	No	2	38.5	0.0	30.8	30.8	100.0
A nominated ER Team member should make a home visit to the family of the deceased student within 24 hours of the death.	No	1	37.5	0.0	42.5	20.0	100.0

In discussing with the family their wishes about what others should be told, A nominated ER Team member should advise them to consider the option of their child only being named to their friends and classmates and referring anonymously to the death with the rest of the school population.	No	1	35.0	2.5	27.5	35.0	100.0
Informing staff							
Where possible, the meeting should be held before students are formally informed.	Yes	1	100.0	0.0	0.0	0.0	100.0
The initial staff meeting should introduce the ER Team.	Yes	1	100.0	0.0	0.0	0.0	100.0
The initial staff meeting should inform staff of any outside support staff (e.g. social workers or mental health staff) who will be assisting and explain their role.	Yes	1	100.0	0.0	0.0	0.0	100.0
The initial staff meeting should identify the ER Team member who staff should come to if they receive any new or relevant information.	Yes	1	100.0	0.0	0.0	0.0	100.0
The initial staff meeting should clarify how phone inquiries are to be managed.	Yes	1	100.0	0.0	0.0	0.0	100.0
The initial staff meeting should tell staff how the school community will be informed.	Yes	1	100.0	0.0	0.0	0.0	100.0
The initial staff meeting should give staff the opportunity to ask questions.	Yes	1	100.0	0.0	0.0	0.0	100.0
The initial staff meeting should advise staff of appropriate language to use when talking about the suicide.	Yes	1	100.0	0.0	0.0	0.0	100.0
The initial staff meeting should brief staff about how to identify students who are distressed and how to support or refer as necessary.	Yes	1	100.0	0.0	0.0	0.0	100.0

The initial staff meeting should provide staff with relevant factsheets (e.g. Grief: How young people might respond to a suicide; How to talk about suicide with young people).	Yes	1	100.0	0.0	0.0	0.0	100.0
The initial staff meeting should look out for staff who may need additional support and, following the meeting, refer them to appropriate services.	Yes	1	100.0	0.0	0.0	0.0	100.0
A nominated ER Team member should ensure that administrative staff receive guidance on how to respond to enquiries concerning the death (e.g. from police, parents, media).	Yes	1	100.0	0.0	0.0	0.0	100.0
The person/people conducting the subsequent staff meetings should, as appropriate, provide up-to-date information.	Yes	1	100.0	0.0	0.0	0.0	100.0
The initial staff meeting should share accurate information about the death.	Yes	1	97.5	2.5	0.0	0.0	100.0
w. Brief staff about how to refer students who may be at increased risk of suicide.	Yes	1	97.5	0.0	2.5	0.0	100.0
The initial staff meeting should provide staff with information about relevant Employee Assistance Programs (EAPs).	Yes	1	97.5	0.0	2.5	0.0	100.0
The initial staff meeting should explain plans for the day, including locations of any support rooms, changes to responsibilities or routines, such as more staff on yard duty and additional measures to monitor student whereabouts.	Yes	1	97.5	0.0	2.5	0.0	100.0
The initial staff meeting should provide staff with the date and time of the next meeting.	Yes	1	97.5	0.0	2.5	0.0	100.0

The person/people conducting the subsequent staff meetings should, as appropriate, remind staff about support services available to them.	Yes	1	97.5	0.0	2.5	0.0	100.0
The initial staff meeting should provide staff with an up-to-date list of services to refer to.	Yes	2	96.2	0.0	3.8	0.0	100.0
The initial staff meeting should instruct staff to refer students to the school wellbeing team.	Yes	2	96.2	0.0	3.8	0.0	100.0
The ER Team should determine the length and number of staff meetings as appropriate.	Yes	2	96.2	0.0	3.8	0.0	100.0
A nominated ER Team member should brief school board members, substitute teachers, sessional or contract staff about the death, and any key points from the meeting relevant to those individuals.	Yes	3	96.0	0.0	4.0	0.0	100.0
A meeting with all school staff should be held as soon as possible after being informed about the suicide.	Yes	1	95.0	0.0	5.0	0.0	100.0
A nominated ER Team member should inform individual staff members about the death ahead of the meeting if they are considered to be particularly vulnerable for any reason.	Yes	1	95.0	2.5	2.5	0.0	100.0
The initial staff meeting should identify which ER Team member has been designated as the media spokesperson and instruct staff to refer all media inquiries to him or her.	Yes	1	95.0	0.0	2.5	2.5	100.0
The initial staff meeting should instruct staff not to talk to the media.	Yes	1	95.0	0.0	5.0	0.0	100.0
The initial staff meeting should advise staff not to spread rumours.	Yes	1	95.0	0.0	0.0	5.0	100.0
The initial staff meeting should provide appropriate staff with a script to read to students informing them of the death.	Yes	1	95.0	0.0	2.5	2.5	100.0

The initial staff meeting should prepare staff for student reactions and questions by discussing the issue with staff.	Yes	1	95.0	0.0	2.5	2.5	100.0
The initial staff meeting should encourage staff to come forward if they feel that they cannot handle some of their usual responsibilities, so that additional support can be provided.	Yes	1	95.0	0.0	5.0	0.0	100.0
The person/people conducting the subsequent staff meetings should, as appropriate, offer verbal appreciation of the staff.	Yes	1	95.0	0.0	2.5	2.5	100.0
The person/people conducting the subsequent staff meetings should, as appropriate, remind staff to continue to monitor high-risk students.	Yes	1	95.0	0.0	5.0	0.0	100.0
The person/people conducting the subsequent staff meetings should, as appropriate, provide a protocol for what will happen the next day.	Yes	1	95.0	0.0	5.0	0.0	100.0
The meeting should be conducted by the principal.	Yes	1	92.5	0.0	7.5	0.0	100.0
The initial staff meeting should allow staff an opportunity to express their own reactions and grief.	Yes	1	92.5	0.0	2.5	5.0	100.0
The initial staff meeting should encourage discussion of how group members can support one another.	Yes	1	92.5	0.0	5.0	2.5	100.0
The initial staff meeting should stress the importance of keeping school routine as normal as possible.	Yes	1	92.5	0.0	7.5	0.0	100.0
If staff feel that they cannot face or handle the class, the ER Team should provide additional support.	Yes	1	92.5	0.0	5.0	2.5	100.0
If it is necessary to inform staff during class time, the ER Team should identify staff who may need additional support, and ensure that this is available.	Yes	1	92.5	2.5	5.0	0.0	100.0

The person/people conducting the subsequent staff meetings should, as appropriate, discuss with staff the implementation of the ER Plan and make amendments as required.	Yes	1	92.5	2.5	2.5	2.5	100.0
The nominated ER Team member should organise a meeting with appropriate staff to discuss upcoming events or activities which might need to be altered or cancelled in view of the suicide (e.g. excursions, graduation).	Yes	1	92.5	0.0	5.0	2.5	100.0
A nominated ER Team member should document actions arising from the meeting, and provide copies to other members of the ER Team	Yes	2	92.3	0.0	0.0	7.7	100.0
The leader of the ER Team should send written communications to staff to share any relevant information as it becomes available.	Yes	2	92.3	0.0	0.0	7.7	100.0
The staff meeting should only be held after the ER Team have met to implement the ER Plan.	Yes	3	92.0	0.0	8.0	0.0	100.0
The initial staff meeting should discuss the principles of postvention, including the school's role.	Yes	1	90.0	2.5	5.0	2.5	100.0
The initial staff meeting should arrange for an alternative person to read the script for any staff who are unable to manage.	Yes	1	90.0	0.0	5.0	5.0	100.0
The person/people conducting the subsequent staff meetings should, as appropriate, discuss the day, including what went well and what did not.	Yes	1	90.0	0.0	5.0	5.0	100.0
The person/people conducting the subsequent staff meetings should, as appropriate, remind staff of the risk factors and warning signs of suicide.	Yes	1	90.0	0.0	7.5	2.5	100.0

The person/people conducting the subsequent staff meetings should, as appropriate, identify staff who may need additional support and, following the meeting, refer them to appropriate services.	Yes	1	90.0	2.5	7.5	0.0	100.0
The person/people conducting the subsequent staff meetings should, as appropriate, remind staff that the deceased student and their family have a right to confidentiality.	Yes	1	90.0	2.5	7.5	0.0	100.0
The person/people conducting the subsequent staff meetings should, as appropriate, discuss any media requests and how these are to be handled.	Yes	1	90.0	0.0	7.5	2.5	100.0
The person/people conducting the subsequent staff meetings should, as appropriate, provide staff with the date and time of the next meeting.	Yes	1	90.0	0.0	10.0	0.0	100.0
The initial staff meeting should provide staff with information and resources on managing social media following a suicide.	Yes	2	88.5	3.8	3.8	3.8	100.0
If possible, a nominated ER Team member should arrange for a mental health professional to attend the meeting, to support and advise staff.	Yes	1	87.5	2.5	10.0	0.0	100.0
The initial staff meeting should tell staff about any family wishes regarding information being shared or withheld.	Yes	1	87.5	0.0	12.5	0.0	100.0
The initial staff meeting should brief staff about suicide contagion and how to identify students who may be at increased risk of suicide.	Yes	1	87.5	0.0	2.5	10.0	100.0
The ER Team should hold a follow-up meeting with staff, preferably at the end of the first day.	Yes	1	87.5	0.0	7.5	5.0	100.0

The person/people conducting the subsequent staff meetings should, as appropriate, allow staff to express their feelings and ask for what they need.	Yes	1	87.5	0.0	10.0	2.5	100.0
The person/people conducting the subsequent staff meetings should, as appropriate, remind staff of the importance of self (e.g. getting good sleep, exercising regularly) and provide of a list of stress management strategies.	Yes	1	87.5	0.0	7.5	5.0	100.0
The person/people conducting the subsequent staff meetings should, as appropriate, talk about how group members can support one another	Yes	1	87.5	2.5	7.5	2.5	100.0
The initial staff meeting should inform staff of protocols regarding memorials.	Yes	1	85.0	7.5	7.5	0.0	100.0
The person/people conducting the subsequent staff meetings should, as appropriate, ask staff to share observations of student reactions and how these were handled.	Yes	1	85.0	0.0	10.0	5.0	100.0
If the death occurs outside of school hours, nominated ER Team members should use a phone tree to contact all staff and inform them that a meeting will be held.	Yes	2	84.6	3.8	7.7	3.8	100.0
A nominated ER Team member should ensure that administrative staff keep a list of all individuals who call for information.	Yes	2	84.6	3.8	11.5	0.0	100.0
The nominated ER Team member should remind staff that upcoming activities that may be difficult for students can provide further opportunities for appropriately acknowledging the loss.	Yes	2	84.6	0.0	11.5	3.8	100.0
The meeting should be conducted by the ER Team leader.	Yes	1	82.5	2.5	10.0	5.0	100.0

If the death occurs outside of school hours, A nominated ER Team member should contact all staff members and inform them that a staff meeting will be held.	Yes	1	82.5	2.5	12.5	2.5	100.0
The initial staff meeting should outline plans for following up all unauthorised/unexplained student absences.	Yes	1	82.5	2.5	12.5	2.5	100.0
The initial staff meeting should inform staff of any arrangements for the funeral or other services, including provision for students and staff to attend.	Yes	1	82.5	10.0	7.5	0.0	100.0
A nominated ER Team member should display information in the staff room about ER Team roles and changes to procedures.	Yes	1	82.5	5.0	12.5	0.0	100.0
The ER Team should arrange ongoing meetings with staff for as long as necessary.	Yes	1	82.5	2.5	12.5	2.5	100.0
Regardless of who conducts the staff meeting, it should be introduced by the principal.	Yes	2	80.8	3.8	15.4	0.0	100.0
The initial staff meeting should advise staff not to ask students for information regarding the suicide.	Yes	2	80.8	0.0	19.2	0.0	100.0
The initial staff meeting should ask staff members to provide a nominated ER Team member with the deceased student's schoolwork including art, assignments, and journals.	Yes	2	80.8	7.7	11.5	0.0	100.0
The initial staff meeting should instruct staff not to discuss the death where students are likely to overhear them.	Yes	2	80.8	0.0	11.5	7.7	100.0
A nominated ER Team member should brief school board members, substitute teachers, sessional or contract staff (e.g. bus drivers, canteen staff, sports coaches) about the death.	Yes	2	80.8	0.0	11.5	7.7	100.0

The initial staff meeting should brief staff about keeping records on all students who are identified as distressed or at increased risk of suicide and any actions taken.	Yes	1	80.0	2.5	12.5	5.0	100.0
The initial staff meeting should review procedures for students leaving the school grounds and tracking student attendance.	Yes	1	80.0	5.0	12.5	2.5	100.0
The person/people conducting the subsequent staff meetings should, as appropriate, disseminate information regarding the death and/or funeral arrangements.	Yes	1	80.0	2.5	15.0	2.5	100.0
The initial staff meeting should inform staff of the warning signs of suicide.	Re-rated in round 2	1	77.5	0.0	12.5	10.0	100.0
A nominated ER Team member should document actions arising from the meeting, and provide copies to all school staff.	Re-rated in round 2	1	77.5	5.0	17.5	0.0	100.0
A nominated ER Team member should ensure that administrative staff keep a list of all individuals who call for information.	Re-rated in round 2	1	77.5	10.0	12.5	0.0	100.0
The person/people conducting the subsequent staff meetings should, as appropriate, discuss high-risk students and any actions taken/to be taken.	Re-rated in round 2	1	77.5	0.0	20.0	2.5	100.0
The staff meeting should only be held after the ER Team have met to implement the ER Plan.	Re-rated in round 3	2	76.9	3.8	11.5	7.7	100.0
The initial staff meeting should inform staff of the warning signs of suicide.	No	2	76.9	7.7	11.5	3.8	100.0

A nominated ER Team member should brief school board members, substitute teachers, sessional or contract staff) about the death, and any key points from the meeting relevant to those individuals.	Re-rated in round 3	2	76.9	3.8	7.7	11.5	100.0
The person/people conducting the subsequent staff meetings should include, as appropriate, a discussion of high-risk students and any actions taken/to be taken.	No	2	76.9	0.0	11.5	11.5	100.0
The initial staff meeting should provide staff with an up-to-date list of services to refer to.	Re-rated in round 2	1	75.0	5.0	15.0	5.0	100.0
A nominated ER Team member should brief school board members, substitute teachers, sessional or contract staff (e.g. bus drivers, canteen staff, sports coaches) about the death, and content of the meeting, including next steps.	Re-rated in round 2	1	72.5	0.0	20.0	7.5	100.0
The initial staff meeting should advise staff not to ask students for information regarding the suicide.	Re-rated in round 2	1	70.0	0.0	22.5	7.5	100.0
The initial staff meeting should ask staff members to provide a nominated ER Team member with the deceased student's schoolwork including art, assignments, and journals.	Re-rated in round 2	1	70.0	2.5	20.0	7.5	100.0
The nominated ER Team member should remind staff that upcoming activities that may be difficult for students can provide further opportunities for appropriately acknowledging the loss.	Re-rated in round 2	1	70.0	5.0	22.5	2.5	100.0
The leader of the ER Team should send written communications to staff to share any additional information as it becomes available.	Re-rated in round 2	1	70.0	2.5	20.0	7.5	100.0

The initial staff meeting should let staff know when they can expect that a deceased student's name will be removed from the roll.	No	1	65.0	15.0	15.0	5.0	100.0
At the end of the first day classroom teachers should make an announcement to their students that includes additional verified information and re-emphasises the continuing availability of in-school resources.	No	3	64.0	8.0	28.0	0.0	100.0
A meeting with all school staff should be held within 24 hours of being informed about the suicide.	No	1	62.5	2.5	25.0	10.0	100.0
If it is not possible to inform staff of the death in a meeting, and there is an urgent need for them to know, the ER Team should call each teacher out of the classroom, telling them about the suicide, what they should do and what will happen next.	No	1	62.5	0.0	22.5	15.0	100.0
These meetings should be held before, after or instead of normal staff meeting business.	No	1	62.5	7.5	27.5	2.5	100.0
The person/people conducting the subsequent staff meetings should, as appropriate, remind staff of the importance of documenting crisis response efforts for future planning and understanding.	No	1	62.5	12.5	12.5	12.5	100.0
A mental health professional should attend the ongoing meetings.	No	1	62.5	7.5	27.5	2.5	100.0
A nominated ER Team member should brief school board members, substitute teachers, sessional or contract staff (e.g. bus drivers, canteen staff, sports coaches) about the death, and content of the meeting, including next steps.	No	2	61.5	7.7	15.4	15.4	100.0
If the death occurs outside of school hours, a nominated ER Team member should contact all staff	No	1	60.0	5.0	17.5	17.5	100.0

members and inform them that a student suicide has occurred.							
If the death occurs outside of school hours, nominated ER Team members should use a phone tree to contact all staff and inform them that a suspected student suicide has occurred.	No	2	57.7	3.8	26.9	11.5	100.0
The ER Team should arrange ongoing meetings with staff every day for approximately five days following the death.	No	1	57.5	5.0	27.5	10.0	100.0
The person/people conducting the subsequent staff meetings should, as appropriate, tell staff that it is reasonable to reduce their expected academic goals following the death.	No	1	57.5	12.5	15.0	15.0	100.0
The initial staff meeting should put the suicide in perspective by discussing facts about the incidence of suicide nationally and relaying information about the experiences of other schools.	No	1	42.5	10.0	20.0	27.5	100.0
The initial staff meeting should advise staff to allow students to clarify or correct facts regarding the suicide.	No	1	42.5	2.5	35.0	20.0	100.0
The leader of the ER Team should send written communications to staff to share any additional information as it becomes available.	No	2	42.3	7.7	19.2	30.8	100.0
A nominated ER Team member should document actions arising from the meeting, and provide copies to all school staff.	No	2	38.5	11.5	26.9	23.1	100.0
The meeting should be held outside of student contact hours.	No	1	35.0	27.5	25.0	12.5	100.0
The person/people conducting the subsequent staff meetings should, as appropriate, ask staff how they	No	1	35.0	22.5	17.5	25.0	100.0

have managed in a similar crisis and brainstorm a list of strategies.							
Subsequent meetings with staff should be held before normal staff meeting business.	No	2	34.6	3.8	50.0	11.5	100.0
The person/people conducting the subsequent staff meetings should, as appropriate, ask staff to write down whether the meeting has been helpful and, if it was, what helped.	No	1	32.5	30.0	22.5	15.0	100.0
Subsequent meetings with staff should be held instead of normal staff meeting business.	No	2	26.9	3.8	50.0	19.2	100.0
The initial staff meeting should provide staff with news articles about the death, if available.	No	1	17.5	22.5	25.0	35.0	100.0
If it is not possible to inform staff of the death in a meeting, and there is an urgent need for them to know, the ER Team should deliver a note to all teachers during class time, telling them about the suicide, what they should do and what will happen next.	No	1	15.0	0.0	15.0	70.0	100.0
If staff feel that they cannot face or handle the class, the ER Team should encourage them to be strong and to take charge for the sake of the students.	No	1	10.0	0.0	10.0	80.0	100.0
The meeting should last at least one hour.	No	1	2.5	32.5	30.0	35.0	100.0
Subsequent meetings with staff should be held after normal staff meeting business.	No	2	0.0	15.4	57.7	26.9	100.0
Informing students							
Teachers should tell the students about the death in small, naturally occurring groups such as homeroom or first period classes.	Yes	1	100.0	0.0	0.0	0.0	100.0

When teachers are informing students about the death they should provide clear and accurate information.	Yes	1	100.0	0.0	0.0	0.0	100.0
When teachers are informing students about the death they should note students who appear to be overly distressed.	Yes	1	100.0	0.0	0.0	0.0	100.0
When teachers are informing students about the death they should inform the students of support resources available at the school and in the community (e.g. external mental health services/school wellbeing staff/support room).	Yes	1	100.0	0.0	0.0	0.0	100.0
When teachers are informing students about the death they should not imply that the deceased student's actions are understandable given their life circumstances.	Yes	2	100.0	0.0	0.0	0.0	100.0
When teachers are informing students about the death they should emphasise the normality of different grief and stress reactions.	Yes	1	97.5	0.0	0.0	2.5	100.0
When teachers are informing students about the death they should not allow other students to glorify or romanticise the deceased student.	Yes	2	96.2	0.0	3.8	0.0	100.0
If a student asks about the method of or reasons for the suicide, the teacher should explain that suicide is complicated and there is usually no single reason.	Yes	2	96.2	0.0	3.8	0.0	100.0
A nominated staff member should provide the closest friends of the deceased student with immediate support and information about where they can receive continuing assistance at school.	Yes	1	95.0	0.0	5.0	0.0	100.0
When teachers are informing students about the death they should note students who are not present so that they can be informed separately.	Yes	1	95.0	0.0	0.0	5.0	100.0

When teachers are informing students about the death they should ask students to tell a member of staff if they have concerns about any fellow students.	Yes	1	95.0	0.0	5.0	0.0	100.0
If individual students are spreading rumours, a member of staff should take them aside and explain that it would be better and safer to share only what is known to be true.	Yes	1	95.0	0.0	5.0	0.0	100.0
When teachers are informing students about the death they should acknowledge that the situation will be distressing to family, friends, and the school community.	Yes	1	92.5	0.0	2.5	5.0	100.0
When teachers are informing students about the death they should dispel false rumours.	Yes	1	92.5	0.0	0.0	7.5	100.0
When teachers are informing students about the death they should talk to students about responsible use of social media.	Yes	1	92.5	0.0	5.0	2.5	100.0
If a student becomes very distressed and would like to leave the room to see a counsellor, they should be accompanied.	Yes	1	92.5	0.0	7.5	0.0	100.0
A nominated ER Team member should inform students and parents/guardians of any significant events, or changes to the school's routine because of a suicide.	Yes	1	92.5	0.0	7.5	0.0	100.0
Informing students about the death in a whole school assembly should be avoided wherever possible.	Yes	2	92.3	0.0	7.7	0.0	100.0
When informing close friends about the death, a nominated ER Team member should ask them about any students who were not obvious close friends of the deceased but may benefit from being informed separately.	Yes	2	92.3	0.0	7.7	0.0	100.0
A nominated staff member should seek the cooperation of those close to the deceased student in	Yes	2	92.3	0.0	3.8	3.8	100.0

not spreading sensitive information about the student or the death, and explain why this is important.							
When teachers are informing students about the death they should tell the students there is professional help available for mental health problems.	Yes	2	92.3	0.0	7.7	0.0	100.0
When teachers are informing students about the death they should not describe anything about the deceased student's life circumstances that might have contributed to the suicide.	Yes	2	92.3	0.0	3.8	3.8	100.0
Students should be informed about the death as soon as possible.	Yes	1	90.0	0.0	7.5	2.5	100.0
A nominated staff member should make arrangements for students to be collected by their parents/guardian if necessary.	Yes	1	90.0	0.0	10.0	0.0	100.0
When teachers are informing students about the death they should follow a prepared script that is tailored according to the age of the students and how close they were to the deceased student.	Yes	1	90.0	0.0	7.5	2.5	100.0
When teachers are informing students about the death they should remind students of the importance of self-care (e.g. getting good sleep, exercising regularly) and provide of a list of stress management strategies.	Yes	2	88.5	3.8	7.7	0.0	100.0
A nominated staff member should offer those closest to the deceased student or those with tragic life circumstances to visit the school wellbeing team.	Yes	2	88.5	0.0	11.5	0.0	100.0
Teachers should conduct the first classes after students are informed in a flexible way (e.g. set work for those who wish to resume lessons, allow other	Yes	2	88.5	0.0	3.8	7.7	100.0

students to write in a journal, speak quietly with each other, or sit and think).							
Informing students about the death by a public announcement system should be avoided wherever possible.	Yes	1	87.5	0.0	2.5	10.0	100.0
The ER Team should make provision to inform students who are not present when other students are informed about the death.	Yes	1	87.5	2.5	7.5	2.5	100.0
When teachers are informing students about the death they should remind students about the importance of supporting each other.	Yes	1	87.5	0.0	10.0	2.5	100.0
When teachers are informing students about the death they should remind students that rumours can be hurtful and unfair to the deceased student, their family, and their friends.	Yes	1	87.5	0.0	5.0	7.5	100.0
When teachers are informing students about the death they should identify an ER Team member that has been designated as the media spokesperson and instruct students to refer any media inquiries to him or her.	Yes	1	87.5	10.0	2.5	0.0	100.0
When teachers are informing students about the death they should instruct students not to talk to the media.	Yes	1	87.5	0.0	12.5	0.0	100.0
When teachers are informing students about the death they should not express any negative judgements about the deceased student's actions or motivations.	Yes	1	87.5	0.0	2.5	10.0	100.0
Informing students about the death in a school bulletin or newsletter should be avoided wherever possible.	Yes	1	85.0	0.0	5.0	10.0	100.0

A nominated staff member should inform the closest friends of the deceased student and any other vulnerable students about the death individually or in appropriate friendship groups.	Yes	1	85.0	0.0	12.5	2.5	100.0
When teachers are informing students about the death they should emphasise that students should not keep secrets about suicide but should tell a member of staff or other responsible adult.	Yes	1	85.0	0.0	12.5	2.5	100.0
Wherever possible, the homeroom or form teacher should inform students about the death.	Yes	2	84.6	0.0	15.4	0.0	100.0
When teachers are informing students about the death they should tell the students there is professional help available for suicidal thoughts.	Yes	2	84.6	0.0	11.5	3.8	100.0
Do not notify students of the death using the internet or social media.	Yes	1	82.5	2.5	10.0	5.0	100.0
Wherever possible, the homeroom or form teacher should inform students about the death.	Yes	1	82.5	2.5	12.5	2.5	100.0
When teachers are informing students about the death they should provide sufficient time to allow students to express their feelings, ask questions and discuss issues.	Yes	1	82.5	0.0	15.0	2.5	100.0
When teachers are informing students about the death they should not divulge the contents of any suicide note.	Yes	1	82.5	0.0	7.5	10.0	100.0
When teachers are informing students about the death they should not glorify or romanticise the deceased student by speaking too positively about them or giving them too much attention.	Yes	1	82.5	2.5	5.0	10.0	100.0

If a student asks about the method of or reasons for the suicide, the teacher should not give details about the specific method used.	Yes	1	82.5	0.0	12.5	5.0	100.0
If a student becomes very distressed and would like to leave the room to see a counsellor, they should be allowed.	Yes	1	82.5	0.0	7.5	10.0	100.0
A nominated ER Team member should assist students to obtain special consideration for external exams if necessary.	Yes	1	82.5	0.0	15.0	2.5	100.0
Informing students about the death in an assembly should be avoided wherever possible.	Yes	1	80.0	0.0	12.5	7.5	100.0
A nominated staff member should seek the cooperation of those close to the deceased student in not spreading sensitive information about the student or the death.	Yes	1	80.0	0.0	12.5	7.5	100.0
When teachers are informing students about the death they should not describe the method of suicide.	Yes	1	80.0	0.0	10.0	10.0	100.0
If a student asks about the method of or reasons for the suicide, the teacher should explain that suicide is complicated and there is no single reason.	Yes	1	80.0	0.0	10.0	10.0	100.0
A nominated ER Team member should change the arrangements for any tests occurring on the day or the day after students have been informed of the death (e.g. in a separate room, more time or cancelled if necessary).	Yes	1	80.0	0.0	20.0	0.0	100.0
When teachers are informing students about the death they should feel able to express their emotions but still manage the situation/class.	Re-rated in round 2	1	77.5	0.0	12.5	10.0	100.0
When teachers are informing students about the death they should not imply that the deceased	Re-rated in round 2	1	77.5	0.0	5.0	17.5	100.0

student's actions are understandable given their life circumstances.							
Teachers should conduct the first classes after students are informed in a flexible way (e.g. set work for those who wish to resume lessons, allow other students to write in a journal, speak quietly with each other, or sit and think).	Re-rated in round 2	1	77.5	0.0	15.0	7.5	100.0
When teachers are informing students about the death they should tell the students there are treatments available to help with mental health problems.	No	2	76.9	0.0	15.4	7.7	100.0
Wherever possible, all students should be told about the death simultaneously.	Re-rated in round 2	1	75.0	5.0	12.5	7.5	100.0
When teachers are informing students about the death they should not describe anything about the deceased student’s life circumstances that might have contributed to the suicide.	Re-rated in round 2	1	75.0	0.0	15.0	10.0	100.0
When teachers are informing students about the death they should not allow other students to glorify or romanticise the deceased student.	Re-rated in round 2	1	75.0	0.0	7.5	17.5	100.0
When teachers are informing students about the death they should allow time for any observances that are part of the schools religious tradition e.g. prayer for the deceased student.	No	2	73.1	0.0	23.1	3.8	100.0
When teachers are informing students about the death they should inform students about the warning signs of suicide to look out for in other students (e.g. helping someone you know).	No	2	73.1	3.8	19.2	3.8	100.0

At the end of the first day, classroom teachers should make an announcement to their students that includes additional verified information and re-emphasises the continuing availability of in-school resources.	Re-rated in round 3	2	73.1	0.0	11.5	15.4	100.0
When teachers are informing students about the death they should allow time for any observances that are part of the schools religious tradition e.g. prayer for the deceased student.	Re-rated in round 2	1	72.5	0.0	20.0	7.5	100.0
When teachers are informing students about the death they should remind students of the importance of self-care (e.g. getting good sleep, exercising regularly) and provide of a list of stress management strategies.	Re-rated in round 2	1	72.5	2.5	12.5	12.5	100.0
When teachers are informing students about the death they should inform students about the warning signs of suicide to look out for in other students (e.g. Helping someone you know).	Re-rated in round 2	1	72.5	0.0	15.0	12.5	100.0
When teachers are informing students about the death they should emphasise that no one is to blame for the suicide.	Re-rated in round 2	1	70.0	0.0	5.0	25.0	100.0
When teachers are informing students about the death they should tell the students there are treatments available to help with mental health problems.	Re-rated in round 2	1	70.0	0.0	12.5	17.5	100.0
When teachers are informing students about the death they should acknowledge that there are alternative solutions to problems.	Re-rated in round 2	1	70.0	0.0	17.5	12.5	100.0

When teachers are informing students about the death they should acknowledge that there are alternative solutions to problems.	No	2	69.2	3.8	15.4	11.5	100.0
When teachers are informing students about the death they should follow a checklist of items to cover.	No	1	67.5	7.5	15.0	10.0	100.0
If a student asks about the method of or reasons for the suicide, the teacher should not give details about the exact time and location of the death.	No	1	67.5	0.0	25.0	7.5	100.0
If a student asks about the method of or reasons for the suicide, the teacher should tell the students that the important thing is to help each other, especially friends and family of the deceased student, rather than think about how the person died.	No	1	67.5	2.5	12.5	17.5	100.0
When teachers are informing students about the death they should feel able to express their emotions but still manage the situation/class.	No	2	65.4	3.8	11.5	19.2	100.0
When teachers are informing students about the death they should emphasise that no one is to blame for the suicide.	No	2	65.4	0.0	26.9	7.7	100.0
When teachers are informing students about the death they should tell the students there are treatments available to help with suicidal thoughts.	No	1	65.0	0.0	10.0	25.0	100.0
When teachers are informing students about the death they should pass funeral details onto students if these are known.	No	1	62.5	2.5	22.5	12.5	100.0
On the second day, the homeroom or form teacher should make an announcement that includes additional verified information and re-emphasises the continuing availability of in-school resources.	No	1	62.5	2.5	27.5	7.5	100.0

If a student asks about the method of or reasons for the suicide, the teacher should keep the focus on suicide in general and not the specific suicide.	No	1	57.5	5.0	25.0	12.5	100.0
If the death occurs while school is in session, students should return to their homeroom classrooms to be informed of the death.	No	1	55.0	0.0	40.0	5.0	100.0
When teachers are informing students about the death they should talk about the association between mental health problems and suicide.	No	1	50.0	0.0	27.5	22.5	100.0
Wherever possible, all students should be told about the death simultaneously.	No	2	50.0	7.7	23.1	19.2	100.0
If a death occurs while school is in session the ER Team should call each teacher out of their classroom, to tell them about the suicide and what they should do, including instructions on how to immediately inform the students about the suicide.	No	1	45.0	0.0	45.0	10.0	100.0
When teachers are informing students about the death they should tell the students that mental health problems can get in the way of a person thinking clearly.	No	1	45.0	2.5	25.0	27.5	100.0
Once students have been informed, normal classroom activities should be resumed.	No	1	45.0	0.0	42.5	12.5	100.0
A nominated staff member should send those closest to the deceased student or those with tragic life circumstances to the school wellbeing team.	No	1	42.5	0.0	42.5	15.0	100.0
When teachers are informing students about the death they should convey the idea that although thoughts of death and taking one's life are normal, suicide is not a normal response to stress.	No	1	37.5	2.5	15.0	45.0	100.0

At the end of the first day, a nominated ER Team member should make an announcement to all students that includes additional verified information and re-emphasises the continuing availability of in-school resources.	No	1	37.5	2.5	30.0	30.0	100.0
If a student asks about the method of or reasons for the suicide, the teacher should give basic facts about the method but don't give graphic details or talk at length about it.	No	1	32.5	0.0	15.0	52.5	100.0
When teachers are informing students about the death they should state that the only one ultimately responsible for the suicide is the deceased student.	No	1	30.0	0.0	17.5	52.5	100.0
The deceased student's name should be included in any communications about the death, unless the family object.	No	1	27.5	2.5	50.0	20.0	100.0
When teachers are informing students about the death they should emphasise that the deceased student was likely suffering from mental health problems, that can cause substantial psychological pain but may not have been apparent to others (or that may have shown as behaviour problems or substance abuse).	No	1	25.0	2.5	27.5	45.0	100.0
Wherever possible, the principal should inform students about the death.	No	1	22.5	32.5	27.5	17.5	100.0
When teachers are informing students about the death they should portray the act as a permanent solution to temporary problems.	No	1	20.0	2.5	35.0	42.5	100.0

If a death occurs while school is in session the ER Team should deliver a note to all teachers during class time, telling them about the suicide and what they should do, including instructions on how to immediately inform the students about the suicide.	No	1	17.5	2.5	30.0	50.0	100.0
The deceased student's name should be included in any communications about the death, only to those close to the student or those in the class of a sibling/cousin.	No	1	17.5	2.5	47.5	32.5	100.0
When teachers are informing students about the death they should help students not to identify themselves with the deceased student, e.g. portray them as a very upset, disturbed, and as someone who had not found an effective way to work out problems.	No	1	15.0	0.0	30.0	55.0	100.0
The deceased student's name should be included in any communications about the death.	No	1	15.0	0.0	55.0	30.0	100.0
When teachers are informing students about the death they should talk about how suicide transfers pain from the person who killed him or herself to a whole community who is now in pain.	No	1	10.0	2.5	25.0	62.5	100.0
When teachers are informing students about the death they should talk about any mental health problems that the deceased student was known to have.	No	1	5.0	0.0	25.0	70.0	100.0
When teachers are informing students about the death, they should not conceal any details.	No	1	2.5	2.5	22.5	72.5	100.0
If a death occurs while school is in session a nominated ER Team member should announce it using the public announcement system, but this announcement should be carefully planned and rehearsed.	No	1	0.0	0.0	5.0	95.0	100.0

Informing parents							
The parent meeting/s should include an outline of the purpose and structure of meeting.	Yes	1	100.0	0.0	0.0	0.0	100.0
The parent meeting/s should include contact information (names, telephone numbers, and addresses) for mental health resources at school and in the community.	Yes	1	100.0	0.0	0.0	0.0	100.0
The parent meeting/s should include an acknowledgement that parents may be affected by the death and encouragement for parents to seek professional help if appropriate.	Yes	2	100.0	0.0	0.0	0.0	100.0
The communication with the parents (e.g. SMS with hyperlink, email, letter) should include support resources available at the school and in the community.	Yes	1	97.5	0.0	0.0	2.5	100.0
The communication with the parents (e.g. SMS with hyperlink, email, letter) should encourage parents to monitor the reactions of their child to the suicide.	Yes	2	96.2	0.0	3.8	0.0	100.0
The parent meeting/s should include the same clear and accurate information about the death that was shared with students.	Yes	2	96.2	0.0	3.8	0.0	100.0
The communication with the parents (e.g. SMS with hyperlink, email, letter) should include a list of resources available to families.	Yes	1	95.0	0.0	2.5	2.5	100.0
The communication with the parents (e.g. SMS with hyperlink, email, letter) should include what to do if they are worried about someone else.	Yes	1	95.0	0.0	2.5	2.5	100.0

The communication with the parents (e.g. SMS with hyperlink, email, letter) should include the name and contact details of a nominated ER Team member who they should direct any questions or concerns to.	Yes	1	95.0	0.0	2.5	2.5	100.0
The parent meeting/s should include an explanation of how the school will identify and support high-risk students.	Yes	1	95.0	0.0	5.0	0.0	100.0
The parent meeting/s should include a discussion of the range of emotional reactions that people may have to the death.	Yes	1	95.0	0.0	2.5	2.5	100.0
The parent meeting/s should include provision of resources about how to talk about suicide with young people (e.g. how to respond to questions about suicide and the link between suicide and mental disorders in young people).	Yes	1	95.0	2.5	2.5	0.0	100.0
The parent meeting/s should include plans for the next week, including any changes to routine or to upcoming events or activities (e.g. projects, plays, research, novels or other items in the curriculum that could invite a focus on suicide).	Yes	1	95.0	0.0	5.0	0.0	100.0
The parent meeting/s should include a discussion with parents about additional needs they may have (e.g. information sessions on recognising signs of suicide risk or understanding grief and loss, support groups for parents led by external mental health or bereavement services).	Yes	1	95.0	0.0	2.5	2.5	100.0
The parent meeting/s should include time for discussion and questions.	Yes	1	95.0	0.0	2.5	2.5	100.0
The communication with the parents (e.g. SMS with hyperlink, email, letter) should include common student reactions to suicide, how to answer difficult	Yes	1	92.5	0.0	5.0	2.5	100.0

questions about suicide and how to talk about suicide with young people.							
The communication with the parents (e.g. SMS with hyperlink, email, letter) should include changes to the school's routine or upcoming events.	Yes	1	92.5	0.0	5.0	2.5	100.0
The parent meeting/s should include an expression of sympathy.	Yes	1	92.5	0.0	5.0	2.5	100.0
The parent meeting/s should include encouragement to parents to contact the school if they have concerns about their child.	Yes	1	92.5	0.0	7.5	0.0	100.0
A nominated ER Team member should ensure information regarding ongoing support available at the school is included in any communications with parents (e.g. using their usual method of communication) following the parent meeting.	Yes	2	92.3	0.0	7.7	0.0	100.0
The communication with the parents (e.g. SMS with hyperlink, email, letter) should encourage parents to talk with their children or have another trusted adult speak with them.	Yes	1	90.0	0.0	7.5	2.5	100.0
Before the meeting begins, a nominated ER Team member should ascertain whether there are any media representatives in the audience and determine if they are to remain for any or all of the meeting.	Yes	1	90.0	0.0	7.5	2.5	100.0
A nominated ER Team member should arrange for the principal to attend the parent meeting.	Yes	1	90.0	0.0	10.0	0.0	100.0
A nominated ER Team member should arrange for wellbeing staff to attend the parent meeting.	Yes	1	90.0	0.0	10.0	0.0	100.0

The parent meeting/s should include identification of which ER Team member has been designated as the media spokesperson and a request that parents refer any media inquiries to him or her.	Yes	1	90.0	5.0	5.0	0.0	100.0
The parent meeting/s should include advice not to spread rumours.	Yes	1	90.0	2.5	5.0	2.5	100.0
The communication with the parents (e.g. SMS with hyperlink, email, letter) should include the same clear and accurate information about the death that was shared with students.	Yes	2	88.5	0.0	11.5	0.0	100.0
The parent meeting/s should include information about the death, as agreed by the ER Team in consultation with mental health and/or suicide prevention/postvention experts.	Yes	2	88.5	0.0	7.7	3.8	100.0
The parent meeting/s should include funeral arrangements and "student release policy".	Yes	2	88.5	0.0	7.7	3.8	100.0
A nominated ER Team member should provide parents with updated information about the school's response as needed.	Yes	2	88.5	0.0	7.7	3.8	100.0
The communication with the parents (e.g. SMS with hyperlink, email, letter) should include changes to attendance/sign in/sign out procedures.	Yes	1	87.5	2.5	7.5	2.5	100.0
The communication with the parents (e.g. SMS with hyperlink, email, letter) should include the date and time of a parent meeting, if one is to be held.	Yes	1	87.5	2.5	7.5	2.5	100.0
The parent meeting/s should include an introduction of the ER Team members.	Yes	1	87.5	2.5	10.0	0.0	100.0
The parent meeting/s should include a discussion of the impact of suicide, the principles of postvention, and the school's ongoing role.	Yes	1	87.5	2.5	7.5	2.5	100.0

School wellbeing staff should be available to talk to parents following the meeting.	Yes	1	87.5	0.0	12.5	0.0	100.0
The parent meeting/s should include information about warning signs of suicide in young people and how to identify students who may be at increased risk.	Yes	1	85.0	0.0	12.5	2.5	100.0
The communication with the parents (e.g. SMS with hyperlink, email, letter) should include resources and information on youth suicide prevention.	Yes	1	82.5	5.0	7.5	5.0	100.0
The communication with the parents (e.g. SMS with hyperlink, email, letter) should encourage parents to focus on the needs of their child and not on specific information about the deceased student.	Yes	1	82.5	0.0	12.5	5.0	100.0
A nominated ER Team member should arrange for the relevant year level coordinator to attend the parent meeting.	Yes	1	82.5	0.0	17.5	0.0	100.0
A nominated ER Team member should arrange for representatives from an external mental health services to attend the parent meeting.	Yes	1	82.5	0.0	17.5	0.0	100.0
The person running the parent meeting should ensure that those attending the meeting avoid scapegoating and blaming.	Yes	1	82.5	0.0	7.5	10.0	100.0
Representatives from any external mental health service should be available to talk to parents following the meeting.	Yes	1	82.5	0.0	15.0	2.5	100.0
The communication with the parents (e.g. SMS with hyperlink, email, letter) should include advice to talk to their child about attendance at the funeral or memorial services.	Yes	2	80.8	0.0	15.4	3.8	100.0

A nominated ER Team member should arrange for mental health professionals from the relevant education department, where available, to attend the parent meeting.	Yes	2	80.8	0.0	19.2	0.0	100.0
The parent meeting/s should include information about the risk of suicide contagion.	Yes	1	80.0	2.5	15.0	2.5	100.0
Do not notify parents of the death using social media.	Yes	3	80.0	0.0	20.0	0.0	100.0
The parent meeting/s should include funeral arrangements and "student release policy".	Re-rated in round 2	1	77.5	5.0	17.5	0.0	100.0
A nominated ER Team member should inform all parents that a death has occurred as soon as possible.	Re-rated in round 3	2	76.9	0.0	11.5	11.5	100.0
The communication with the parents (e.g. SMS with hyperlink, email, letter) should include the warning signs of suicide.	Re-rated in round 2	1	75.0	0.0	17.5	7.5	100.0
The communication with the parents (e.g. SMS with hyperlink, email, letter) should encourage parents to talk to their child about attendance at the funeral or memorial services.	Re-rated in round 2	1	75.0	0.0	20.0	5.0	100.0
The parent meeting/s should include clear and accurate information about the death.	Re-rated in round 2	1	75.0	0.0	25.0	0.0	100.0
The parent meeting/s should include information about any family wishes regarding information being shared or withheld.	Re-rated in round 2	1	75.0	0.0	20.0	5.0	100.0
Do not notify parents of the death using social media.	Re-rated in round 3	2	73.1	0.0	19.2	7.7	100.0
The communication with the parents (e.g. SMS with hyperlink, email, letter) should include advice to	No	2	73.1	0.0	23.1	3.8	100.0

accompany their child to the funeral, if they are attending.							
The communication with the parents (e.g. SMS with hyperlink, email, letter) should include clear and accurate information about the death.	Re-rated in round 2	1	72.5	0.0	22.5	5.0	100.0
The meeting should be conducted by the principal.	Re-rated in round 2	1	72.5	0.0	27.5	0.0	100.0
A nominated ER Team member should inform all parents that a death has occurred as soon as possible.	No	3	72.0	0.0	20.0	8.0	100.0
A nominated ER Team member should inform all parents that the suicide has occurred as soon as possible.	Re-rated in round 2	1	70.0	0.0	25.0	5.0	100.0
The communication with the parents (e.g. SMS with hyperlink, email, letter) should include funeral arrangements (if known) and consent requirements for attendance.	Re-rated in round 2	1	70.0	0.0	20.0	10.0	100.0
The communication with the parents (e.g. SMS with hyperlink, email, letter) should encourage parents to accompany their child to the funeral, if they are attending.	Re-rated in round 2	1	70.0	0.0	27.5	2.5	100.0
The meeting should be conducted by a nominated member of the ER Team.	Re-rated in round 2	1	70.0	5.0	25.0	0.0	100.0
The ER Team should organise a meeting for parents, which may involve all parents or some parents, based on perceived need.	No	2	69.2	3.8	23.1	3.8	100.0

The communication with the parents (e.g. SMS with hyperlink, email, letter) should encourage parents to monitor their child's use of the internet to communicate about the death, or to express suicidal feelings.	No	1	67.5	0.0	25.0	7.5	100.0
The communication with the parents (e.g. SMS with hyperlink, email, letter) should include clear and accurate information about the death.	No	2	65.4	0.0	15.4	19.2	100.0
The communication with the parents (e.g. SMS with hyperlink, email, letter) should include the warning signs of suicide.	No	2	65.4	3.8	15.4	15.4	100.0
The communication with the parents (e.g. SMS with hyperlink, email, letter) should include funeral arrangements (if known) and consent requirements for attendance.	No	2	65.4	0.0	23.1	11.5	100.0
The parent meeting/s should include information about any family wishes regarding information being shared or withheld.	No	2	65.4	3.8	19.2	11.5	100.0
At the time of the parent meeting, a nominated ER Team member should provide supervision for students or other children who may attend with their parents.	No	1	62.5	10.0	20.0	7.5	100.0
The parent meeting should be conducted by a nominated member of the ER Team with support from the principal.	No	2	61.5	3.8	30.8	3.8	100.0
The parent meeting should be conducted by the principal.	No	2	57.7	3.8	34.6	3.8	100.0
The parent meeting/s should include clear and accurate information about the death.	No	2	57.7	7.7	23.1	11.5	100.0
Do not notify parents of the death using the internet or social media.	No	1	57.5	2.5	30.0	10.0	100.0

A nominated ER Team member should arrange for all ER Team members to attend the parent meeting.	No	1	55.0	15.0	22.5	7.5	100.0
A nominated ER Team member should arrange for the class teacher to attend the parent meeting.	No	1	55.0	12.5	27.5	5.0	100.0
If necessary, a nominated ER Team member should arrange for security to assist with the flow of traffic and crowd control.	No	1	52.5	17.5	20.0	10.0	100.0
The parent meeting should be conducted by a nominated member of the ER Team.	No	2	50.0	0.0	38.5	11.5	100.0
If school-based ER Team members/the principal are unable to conduct the parent meeting, they should enlist an external mental health professional/agency to take on this role.	No	2	46.2	0.0	38.5	15.4	100.0
A nominated ER Team member should organise a meeting open to all parents.	No	1	45.0	5.0	40.0	10.0	100.0
The parent meeting/s should include recommendations to restrict access to means of suicide or harm (e.g. a sharp object) and/or alcohol or drugs.	No	1	45.0	2.5	30.0	22.5	100.0
The meeting should be conducted by representatives from an external mental health service.	No	1	40.0	5.0	37.5	17.5	100.0
A nominated ER Team member should provide parents with updated information about the school’s response on a daily basis.	No	1	40.0	12.5	32.5	15.0	100.0
A nominated ER Team member should organise a separate meeting for parents of the deceased student's classmates and close friends.	No	1	37.5	5.0	50.0	7.5	100.0
Representatives from any external mental health service should take part only when mental health issues arise in the meeting.	No	1	35.0	10.0	35.0	20.0	100.0

A nominated ER Team member should post any communications regarding the death on the parents' section of the school website, where available.	No	1	27.5	0.0	22.5	50.0	100.0
A nominated ER Team member should inform all parents that a suicide has occurred as soon as possible.	No	2	26.9	0.0	34.6	38.5	100.0
A nominated ER Team member should inform all parents that a "suspected suicide" has occurred as soon as possible.	No	2	26.9	0.0	46.2	26.9	100.0
A nominated ER Team member should organise a separate meeting for parents of students in each year.	No	1	20.0	7.5	55.0	17.5	100.0
Informing the wider community							
A nominated ER Team member should inform any service responsible for providing suicide postvention support to schools of the suicide and the school's response as soon as possible.	Yes	1	100.0	0.0	0.0	0.0	100.0
If a death occurs at the end of a school term, a nominated ER Team member should inform local external mental health services, in case of additional demand.	Yes	1	97.5	0.0	2.5	0.0	100.0
A nominated ER Team member should liaise with any community response team that has been established in response to previous suicides.	Yes	1	97.5	0.0	2.5	0.0	100.0
A nominated ER Team member should inform the relevant education department of the suicide and the school's response as soon as possible.	Yes	1	95.0	0.0	2.5	2.5	100.0
A nominated ER Team member should inform Principals of schools attended by the deceased student's siblings or known close friends of the suicide and the school's response as soon as possible.	Yes	1	95.0	0.0	5.0	0.0	100.0

A nominated ER Team member should inform any service that is responsible for providing mental health support to the school staff of the suicide and the school's response as soon as possible.	Yes	1	95.0	0.0	5.0	0.0	100.0
A nominated ER Team member should inform relevant external mental health services of the suicide and the school's response as soon as possible.	Yes	1	92.5	2.5	5.0	0.0	100.0
A nominated ER Team member should inform the school board/council of the suicide and the school's response as soon as possible.	Yes	2	92.3	0.0	7.7	0.0	100.0
A nominated ER Team member should inform Principals of schools in neighbouring school districts, where appropriate of the suicide and the school's response as soon as possible.	Yes	1	85.0	2.5	10.0	2.5	100.0
A nominated ER Team member should inform the police of the suicide and the school's response as soon as possible.	Yes	2	84.6	0.0	11.5	3.8	100.0
A nominated ER Team member should inform school welfare staff members at schools attended by the deceased student's siblings of the suicide and the school's response as soon as possible.	Yes	1	80.0	2.5	12.5	5.0	100.0
A nominated ER Team member should inform local sports groups and other agencies that the deceased student was involved with of the suicide and the school's response as soon as possible.	Yes	1	80.0	5.0	12.5	2.5	100.0
A nominated ER Team member should inform the police of the suicide and the school's response as soon as possible.	Re-rated in round 2	1	75.0	7.5	12.5	5.0	100.0

A nominated ER Team member should inform the school board/council of the suicide and the school's response as soon as possible.	Re-rated in round 2	1	72.5	2.5	20.0	5.0	100.0
A nominated ER Team member should inform faith organisations of the suicide and the school's response as soon as possible.	No	1	47.5	7.5	37.5	7.5	100.0
If a suicide occurs in the school holidays, the school should arrange for a member of the school wellbeing team to be onsite in the week following the death, with posters put up in school windows to announce this to the community.	No	2	23.1	3.8	46.2	26.9	100.0
Dealing with the media							
There should be designated media spokesperson (who may be based in the relevant education department, or in the school, depending on local policies).	Yes	2	96.2	0.0	0.0	3.8	100.0
There should be a back-up media spokesperson should the designated person be absent.	Yes	2	92.3	3.8	0.0	3.8	100.0
When liaising with the media, the media spokesperson should follow national guidelines for the reporting of suicide (e.g. Mindframe guidelines)	Yes	1	90.0	0.0	7.5	2.5	100.0
All information that is included in any media statements should be checked and cleared by the principal.	Yes	2	88.5	0.0	7.7	3.8	100.0
When discussing with the family about the information to be released to the media, the media spokesperson should make the family aware of the importance of following national guidelines for the reporting of suicide (e.g. Mindframe guidelines).	Yes	2	88.5	3.8	3.8	3.8	100.0

The ER Team should forbid media representatives to conduct interviews on the school grounds.	Yes	2	88.5	0.0	11.5	0.0	100.0
The ER Team should designate a media spokesperson to liaise with the media.	Yes	1	85.0	2.5	10.0	2.5	100.0
Any media statement should express the school's sympathy to the survivors of the deceased student.	Yes	1	85.0	0.0	7.5	7.5	100.0
The media spokesperson should refer media representatives to national guidelines for the reporting of suicide (e.g. Mindframe guidelines)	Yes	1	82.5	2.5	10.0	5.0	100.0
The designated media spokesperson should have media training.	Yes	2	80.8	3.8	11.5	3.8	100.0
If the media spokesperson is based at the relevant education department a nominated ER Team member should provide them with a copy of the ER Plan.	Yes	2	80.8	0.0	11.5	7.7	100.0
The media spokesperson should liaise with the relevant spokesperson at the education department.	Yes	1	80.0	2.5	15.0	2.5	100.0
Any media statement should include information about the school's response.	Yes	1	80.0	0.0	17.5	2.5	100.0
All information that is included in any media statements should be checked and cleared by the principal.	Re-rated in round 2	1	77.5	2.5	15.0	5.0	100.0
The media spokesperson should prepare a statement to read to the media.	No	2	76.9	0.0	19.2	3.8	100.0
The designated media spokesperson should have media training.	Re-rated in round 2	1	75.0	5.0	15.0	5.0	100.0
The media spokesperson should prepare a statement to read to the media.	Re-rated in round 2	1	70.0	2.5	17.5	10.0	100.0

The media spokesperson should respond to inquiries from the media with clear and accurate information.	Re-rated in round 2	1	70.0	2.5	17.5	10.0	100.0
When discussing with the family about the information to be released to the media, the media spokesperson should make the family aware of the importance of following national guidelines for the reporting of suicide (e.g. Mindframe guidelines)	Re-rated in round 2	1	70.0	2.5	22.5	5.0	100.0
The ER Team should forbid media representatives to conduct interviews on the school grounds.	Re-rated in round 2	1	70.0	0.0	20.0	10.0	100.0
The media spokesperson should respond to inquiries from the media with clear and accurate information.	No	2	69.2	0.0	23.1	7.7	100.0
The ER Team should exclude media representatives from parent and student group meetings.	No	1	60.0	0.0	35.0	5.0	100.0
The ER Team should keep the media spokesperson updated about facts as they are received.	No	1	60.0	2.5	20.0	17.5	100.0
If the designated media spokesperson is based within the school, they should be the principal.	No	2	57.7	3.8	30.8	7.7	100.0
Any media statement should be made available to staff members who may be approached by the media, in case the media spokesperson is absent.	No	1	50.0	0.0	22.5	27.5	100.0
The designated media spokesperson should be the principal.	No	1	47.5	7.5	35.0	10.0	100.0
All information that is included in any media statements should be checked and cleared by the family of the deceased student.	No	1	47.5	2.5	35.0	15.0	100.0
Any media statement should state that no one thing or person is to blame.	No	1	47.5	0.0	25.0	27.5	100.0

If the designated media spokesperson is based within the school, they should be a nominated ER Team member.	No	2	46.2	0.0	34.6	19.2	100.0
The designated media spokesperson should be the nominated ER Team member.	No	1	45.0	2.5	40.0	12.5	100.0
The school should not talk to the media about the death.	No	1	32.5	2.5	42.5	22.5	100.0
If the school receive numerous requests for information from the media, the media spokesperson should plan and coordinate a press conference.	No	1	30.0	2.5	50.0	17.5	100.0
The media spokesperson should work with the media to downplay the incident.	No	1	20.0	2.5	25.0	52.5	100.0
Any media statement should convey the idea that a life was cut short that could have been saved.	No	1	17.5	0.0	22.5	60.0	100.0
Any media statement should report on the student's school activities (but the student should not be made into a hero or outstanding individual if this was not the case).	No	1	17.5	2.5	35.0	45.0	100.0
High-risk students							
Staff should immediately tell an ER Team member if there are suicide-related rumours, notes, or messages amongst the students.	Yes	1	100.0	0.0	0.0	0.0	100.0
Staff should support and respond to high-risk students by referring the student to the school wellbeing team and/or a nominated ER Team member.	Yes	1	100.0	0.0	0.0	0.0	100.0
Staff should not guarantee confidentiality to a student if they are concerned about the student's safety.	Yes	1	100.0	0.0	0.0	0.0	100.0
A nominated ER Team member should keep a record of safety and support plans.	Yes	1	100.0	0.0	0.0	0.0	100.0

If a nominated ER Team member contacts the parents/guardians of a high risk student, they should, as appropriate discuss warning signs that could suggest further difficulties for their child.	Yes	2	100.0	0.0	0.0	0.0	100.0
If a student expresses that they are not able to keep themselves safe, school wellbeing staff should (as appropriate not leave the student alone.	Yes	2	100.0	0.0	0.0	0.0	100.0
If a student expresses that they are able to keep themselves safe but remains a cause for concern (e.g. showing symptoms or signs of depression, experiencing thoughts of suicide) school wellbeing staff should (as appropriate) contact their parent or guardian as soon as possible.	Yes	2	100.0	0.0	0.0	0.0	100.0
If a student's behaviour indicates that they are an immediate danger to themselves or others school wellbeing staff should (as appropriate) not leave the student alone.	Yes	2	100.0	0.0	0.0	0.0	100.0
If a student's behaviour indicates that they are an immediate danger to themselves or others school wellbeing staff should (as appropriate) contact their parent or guardian as soon as possible.	Yes	2	100.0	0.0	0.0	0.0	100.0
If a member of the school wellbeing team contacts the parents/guardians, they should, as appropriate discuss warning signs that could suggest further difficulties for their child.	Yes	2	100.0	0.0	0.0	0.0	100.0
If a member of the school wellbeing team contacts the parents/guardians, they should, as appropriate suggest sources of help (e.g. external mental health services, websites).	Yes	2	100.0	0.0	0.0	0.0	100.0

A nominated ER Team member should oversee and coordinate the identification, support and referral of high-risk students and staff.	Yes	1	97.5	0.0	0.0	2.5	100.0
Staff should pass on names and other relevant information about high-risk students to a nominated ER Team member and/or the school wellbeing team.	Yes	1	97.5	0.0	2.5	0.0	100.0
If a student is distressed, staff members should encourage them to talk to a trusted adult.	Yes	1	97.5	0.0	2.5	0.0	100.0
Staff should support and respond to high-risk students listening in a non-judgemental and empathic way to students in distress.	Yes	1	97.5	0.0	0.0	2.5	100.0
If a student expresses that they are not able to keep themselves safe, a nominated ER Team member should (as appropriate) not leave the student alone.	Yes	1	97.5	0.0	2.5	0.0	100.0
If a student's behaviour indicates that they are an immediate danger to themselves or others a nominated ER Team member should (as appropriate) not leave the student alone.	Yes	1	97.5	0.0	2.5	0.0	100.0
A nominated ER Team member should keep a record of follow-up support provided to students.	Yes	1	97.5	0.0	2.5	0.0	100.0
The ER Team should post a list on the school noticeboard, in the school newsletter and/or on the school website of mental health services families could contact.	Yes	1	97.5	0.0	2.5	0.0	100.0
If the ER Team is concerned about an emerging suicide cluster, they should meet to discuss what additional actions they could take.	Yes	1	97.5	0.0	2.5	0.0	100.0

If a student is highly distressed and would like to go home, a nominated ER Team member should contact their parents and remain with them until their parents are able to pick them up from school.	Yes	2	96.2	3.8	0.0	0.0	100.0
If a nominated ER Team member contacts the parents/guardians of a high risk student, they should, as appropriate encourage the parent to discuss this with their child.	Yes	2	96.2	0.0	3.8	0.0	100.0
A member of the school wellbeing team should provide initial support to any high-risk student, and then refer to an external mental health service as appropriate.	Yes	2	96.2	0.0	3.8	0.0	100.0
If a member of the school wellbeing team contacts the parents/guardians, they should, as appropriate encourage the parent/guardian to contact the student's therapist or counsellor if they are already receiving services from an external mental health service.	Yes	2	96.2	0.0	3.8	0.0	100.0
The ER Team should inform staff about how to support and respond to high-risk students.	Yes	1	95.0	0.0	5.0	0.0	100.0
If a student expresses that they are not able to keep themselves safe, a nominated ER Team member should (as appropriate) contact their parent or guardian as soon as possible.	Yes	1	95.0	0.0	5.0	0.0	100.0
If a student's behaviour indicates that they are an immediate danger to themselves or others a nominated ER Team member should (as appropriate) contact their parent or guardian as soon as possible.	Yes	1	95.0	0.0	5.0	0.0	100.0
A nominated ER Team member should keep a record of students who have undergone an individual risk assessment.	Yes	1	95.0	0.0	5.0	0.0	100.0

A nominated ER Team member should keep a record of outcomes of individual risk assessments and/or screening.	Yes	1	95.0	0.0	5.0	0.0	100.0
A nominated ER Team member should keep a record of students who have been referred to external mental health services.	Yes	1	95.0	0.0	2.5	2.5	100.0
If a student is absent from school without permission, a nominated ER Team member should contact the student's parents.	Yes	1	95.0	0.0	5.0	0.0	100.0
The ER Team should provide all staff with a list of risk factors for suicide in young people (e.g. f/s risk factors)	Yes	1	92.5	0.0	7.5	0.0	100.0
School wellbeing staff and external mental health professionals should identify high-risk students as soon as possible.	Yes	1	92.5	0.0	7.5	0.0	100.0
If a student is highly distressed, a member of staff should remain with them.	Yes	1	92.5	0.0	7.5	0.0	100.0
If a student expresses that they are able to keep themselves safe but remains a cause for concern (e.g. showing symptoms or signs of depression, experiencing thoughts of suicide) a nominated ER Team member should (as appropriate contact their parent or guardian as soon as possible.	Yes	1	92.5	0.0	7.5	0.0	100.0
A nominated ER Team member should keep a record of students who have been identified as high risk.	Yes	1	92.5	0.0	5.0	2.5	100.0
All staff should identify high-risk students as soon as possible.	Yes	2	92.3	0.0	7.7	0.0	100.0
School wellbeing staff should contact known high risk students and offer them support.	Yes	2	92.3	0.0	7.7	0.0	100.0

If a student expresses they are not able to keep themselves safe, or if their behaviour indicates that they are an immediate danger to themselves or others, a staff member should not leave them alone, & should take them to the wellbeing staff.	Yes	2	92.3	0.0	3.8	3.8	100.0
If a student expresses that they are not able to keep themselves safe, a nominated ER Team member should (as appropriate), contact an appropriate mental health service.	Yes	2	92.3	0.0	7.7	0.0	100.0
If a student expresses that they are not able to keep themselves safe, school wellbeing staff should (as appropriate contact their parent or guardian as soon as possible.	Yes	2	92.3	0.0	7.7	0.0	100.0
If a student expresses that they are not able to keep themselves safe, school wellbeing staff should (as appropriate contact an appropriate mental health service.	Yes	2	92.3	0.0	7.7	0.0	100.0
If a student's behaviour indicates that they are an immediate danger to themselves or others school wellbeing staff should (as appropriate) remove access to means of suicide or harm (e.g. a sharp object) and/or alcohol or drugs.	Yes	2	92.3	0.0	7.7	0.0	100.0
If a member of the school wellbeing team contacts the parents/guardians, they should, as appropriate encourage the parent/guardian to discuss this with their child.	Yes	2	92.3	0.0	7.7	0.0	100.0
For any student who undergoes a risk assessment, school wellbeing staff should, in collaboration with the student, contact the parents.	Yes	3	92.0	0.0	4.0	4.0	100.0
The ER Team should provide all staff with a list of warning signs for suicide in young people (e.g. f/s warning signs).	Yes	1	90.0	0.0	7.5	2.5	100.0

A nominated ER Team member should keep a record of students who have undergone screening.	Yes	1	90.0	0.0	5.0	5.0	100.0
Any students who are identified as potentially at high risk via screening should be referred to the school wellbeing team for an individual risk assessment and support.	Yes	2	88.5	0.0	7.7	3.8	100.0
Individual suicide risk assessments, safety plans, and support plans should be developed in collaboration with a mental health professional.	Yes	2	88.5	0.0	11.5	0.0	100.0
If a student expresses they are able to keep themselves safe but remains a cause for concern a nominated ER Team member should, where appropriate, not leave the student alone.	Yes	2	88.5	0.0	11.5	0.0	100.0
If a student's behaviour indicates that they are an immediate danger to themselves or others, school wellbeing staff should (as appropriate) contact an appropriate mental health service.	Yes	3	88.0	0.0	8.0	4.0	100.0
If a student's behaviour indicates that they are an immediate danger to themselves or others a nominated ER Team member should (as appropriate) remove access to means of suicide or harm (e.g. a sharp object) and/or alcohol or drugs.	Yes	1	87.5	0.0	10.0	2.5	100.0
If a nominated ER Team member contacts the parents, they should, as appropriate encourage the parent to contact the student's therapist or counsellor if they are already receiving services from an external mental health service.	Yes	1	87.5	0.0	12.5	0.0	100.0
The ER Team should inform staff about how to identify high-risk students.	Yes	1	85.0	0.0	10.0	5.0	100.0
Any students who are identified as potentially at high risk via other sources should be referred to the school	Yes	1	85.0	0.0	15.0	0.0	100.0

wellbeing team for an individual risk assessment and support.							
If a nominated ER Team member contacts the parents, they should, as appropriate suggest sources of help (e.g. external mental health services, websites).	Yes	1	85.0	5.0	10.0	0.0	100.0
If a student is absent from school without permission, a nominated ER Team member should contact the police if there is concern for the student's safety.	Yes	1	85.0	2.5	12.5	0.0	100.0
The ER Team should provide all staff with a list of the types of students who may be at high risk of suicide following the suicide of a fellow student.	Yes	2	84.6	0.0	7.7	7.7	100.0
If a member of the school wellbeing team contacts the parents/guardians, they should, as appropriate inform them that their child has undergone a risk assessment and the outcome of this.	Yes	2	84.6	0.0	15.4	0.0	100.0
If a member of the school wellbeing team contacts the parents/guardians, they should, as appropriate advise them to remove access to means of suicide or harm (e.g. a sharp object) and/or alcohol or drugs.	Yes	2	84.6	0.0	15.4	0.0	100.0
The ER Team should decide who conducts individual suicide risk assessments and develops safety and support plans according to resources and staffing at the school.	Yes	3	84.0	0.0	12.0	4.0	100.0
Nominated staff members should circulate among students when they are using common areas of the school (e.g. hallways, cafeteria) in order to identify any students who need support.	Yes	1	82.5	0.0	10.0	7.5	100.0

The ER Team should prepare a list of high-risk students.	Yes	1	82.5	2.5	15.0	0.0	100.0
Staff should inform the ER Team of any student absences.	Yes	1	82.5	0.0	17.5	0.0	100.0
A nominated ER Team member should check whether students arrived at the services to which they were referred.	Yes	2	80.8	0.0	19.2	0.0	100.0
If a student expresses that they are not able to keep themselves safe, school wellbeing staff should (as appropriate remove access to means of suicide or harm (e.g. a sharp object) and/or alcohol or drugs.	Yes	2	80.8	0.0	19.2	0.0	100.0
If a student expresses that they are able to keep themselves safe but remains a cause for concern (e.g. showing symptoms or signs of depression, experiencing thoughts of suicide) school wellbeing staff should (as appropriate) contact an appropriate mental health service.	Yes	2	80.8	0.0	19.2	0.0	100.0
If a member of the school wellbeing team contacts the parents/guardians, they should, as appropriate inform them that there has been a suicide of a student at the school.	Yes	2	80.8	0.0	15.4	3.8	100.0
Nominated staff members should ask students to tell them if they are worried about anyone who may be at high risk of suicide.	Yes	1	80.0	2.5	7.5	10.0	100.0
If a student expresses that they are not able to keep themselves safe, a nominated ER Team member should (as appropriate) remove access to means of suicide or harm (e.g. a sharp object) and/or alcohol or drugs.	Yes	1	80.0	2.5	12.5	5.0	100.0

If a student's behaviour indicates that they are an immediate danger to themselves or others a nominated ER Team member should (as appropriate) call 000 or escort them to the emergency department.	Yes	1	80.0	2.5	15.0	2.5	100.0
If a nominated ER Team member contacts the parents, they should, as appropriate inform them that there has been a suicide of a student at the school.	Yes	1	80.0	0.0	20.0	0.0	100.0
If a nominated ER Team member contacts the parents, they should, as appropriate inform them that their child has undergone a risk assessment and the outcome of this.	Yes	1	80.0	0.0	20.0	0.0	100.0
The ER Team should provide all staff with a list of the types of students who may be at high risk of suicide following the suicide of a fellow student.	Re-rated in round 2	1	77.5	5.0	15.0	2.5	100.0
If a student expresses that they are not able to keep themselves safe, a nominated ER Team member should (as appropriate) contact an appropriate mental health service.	Re-rated in round 2	1	77.5	0.0	22.5	0.0	100.0
If a student is highly distressed, a nominated ER Team member should offer them support and make a safety plan with them.	Re-rated in round 3	2	76.9	0.0	15.4	7.7	100.0
The ER Team should decide who conducts individual suicide risk assessments and develops safety and support plans according to resources and staffing at the school.	Re-rated in round 3	2	76.9	0.0	11.5	11.5	100.0
If a student is highly distressed, a nominated ER Team member should offer them support and make a safety plan with them.	No	3	76.0	0.0	8.0	16.0	100.0
School staff who have been trained in suicide risk assessment should conduct individual suicide risk assessments with students identified as being at high risk.	No	3	76.0	0.0	16.0	8.0	100.0

All staff should identify high-risk students as soon as possible.	Re-rated in round 2	1	75.0	0.0	17.5	7.5	100.0
Nominated staff members should contact known high-risk students and offer them support.	Re-rated in round 2	1	75.0	0.0	20.0	5.0	100.0
Individual suicide risk assessments, safety plans, and support plans* should be developed in collaboration with a mental health professional.	Re-rated in round 2	1	75.0	2.5	22.5	0.0	100.0
If a nominated ER Team member contacts the parents, they should, as appropriate encourage the parent to discuss this with their child.	Re-rated in round 2	1	75.0	0.0	25.0	0.0	100.0
A nominated ER Team member should check whether students arrived at the services to which they were referred.	Re-rated in round 2	1	75.0	5.0	17.5	2.5	100.0
A member of the school wellbeing team should provide individual counselling or support to any high-risk student.	Re-rated in round 2	1	75.0	0.0	22.5	2.5	100.0
School staff who have been trained in suicide risk assessment should conduct individual suicide risk assessments with students identified as being at high risk.	Re-rated in round 3	2	73.1	0.0	15.4	11.5	100.0
If a student's behaviour indicates that they are an immediate danger to themselves or others school wellbeing staff should (as appropriate) contact an appropriate mental health service.	Re-rated in round 3	2	73.1	0.0	23.1	3.8	100.0
For any student who undergoes a risk assessment, school wellbeing staff should, in collaboration with the student, contact the parents.	Re-rated in round 3	2	73.1	0.0	26.9	0.0	100.0

If a student expresses that they are able to keep themselves safe but remains a cause for concern (e.g. showing symptoms or signs of depression, experiencing thoughts of suicide) a nominated ER Team member should (as appropriate not leave the student alone.	Re-rated in round 2	1	72.5	2.5	17.5	7.5	100.0
If a nominated ER Team member contacts the parents, they should, as appropriate discuss warning signs that could suggest further difficulties for their child.	Re-rated in round 2	1	72.5	2.5	25.0	0.0	100.0
Any students who are identified as potentially at high risk via screening should be referred to the school wellbeing team for an individual risk assessment and support.	Re-rated in round 2	1	70.0	0.0	12.5	17.5	100.0
If a student's behaviour indicates that they are an immediate danger to themselves or others school wellbeing staff should (as appropriate) call 000 or escort them to the emergency department.	No	2	69.2	0.0	26.9	3.8	100.0
If a member of the school wellbeing team contacts the parents/guardians, they should, as appropriate invite them to a special meeting to help them understand and help their child cope with his or her feelings.	No	2	69.2	3.8	15.4	11.5	100.0
Nominated staff members should contact known high-risk students and offer them support.	No	2	65.4	0.0	15.4	19.2	100.0
School staff who have been trained in suicide risk assessment should develop a safety plan (e.g. safety plan) with each student identified as being at high risk.	No	2	65.4	0.0	26.9	7.7	100.0
School staff who have been trained in suicide risk assessment should develop a support plan for each student identified as being at high risk.	No	2	65.4	0.0	23.1	11.5	100.0

If a student expresses that they are able to keep themselves safe but remains a cause for concern (e.g. showing symptoms or signs of depression, experiencing thoughts of suicide) school wellbeing staff should (as appropriate) not leave the student alone.	No	2	65.4	3.8	26.9	3.8	100.0
If a student expresses that they are able to keep themselves safe but remains a cause for concern (e.g. showing symptoms or signs of depression, experiencing thoughts of suicide) a nominated ER Team member should (as appropriate remove access to means of suicide or harm (e.g. a sharp object) and/or alcohol or drugs.	No	1	65.0	5.0	22.5	7.5	100.0
If a student expresses that they are able to keep themselves safe but remains a cause for concern (e.g. showing symptoms or signs of depression, experiencing thoughts of suicide) a nominated ER Team member should (as appropriate contact an appropriate mental health service.	No	1	65.0	5.0	30.0	0.0	100.0
If a student's behaviour indicates that they are an immediate danger to themselves or others a nominated ER Team member should (as appropriate) contact an appropriate mental health service.	No	1	62.5	5.0	22.5	10.0	100.0
For any student who undergoes a risk assessment, a nominated ER Team member should, in collaboration with the student, contact the parents.	No	1	62.5	5.0	30.0	2.5	100.0
If a nominated ER Team member contacts the parents, they should, as appropriate, advise them to remove access to means of suicide or harm (e.g. a sharp object) and/or alcohol or drugs.	No	1	62.5	0.0	35.0	2.5	100.0

A nominated ER Team member should develop a support plan for each student identified as being at high risk.	No	1	55.0	0.0	37.5	7.5	100.0
If a nominated ER Team member contacts the parents, they should, as appropriate invite them to a special meeting to help them understand and help their child cope with his or her feelings.	No	1	55.0	5.0	35.0	5.0	100.0
A nominated ER Team member should follow the schedule of the deceased student throughout the day in order to identify and follow up with high-risk students and staff.	No	1	55.0	2.5	22.5	20.0	100.0
A nominated ER Team member should follow the schedule of the deceased student throughout the day in order to answer questions from students and staff.	No	1	55.0	2.5	22.5	20.0	100.0
A nominated ER Team member should follow the schedule of the deceased student throughout the day in order to address rumours and myths about the suicide.	No	1	55.0	2.5	22.5	20.0	100.0
If a student expresses that they are able to keep themselves safe but remains a cause for concern (e.g. showing symptoms or signs of depression, experiencing thoughts of suicide) school wellbeing staff should (as appropriate) remove access to means of suicide or harm (e.g. a sharp object) and/or alcohol or drugs.	No	2	53.8	7.7	30.8	7.7	100.0
A nominated ER Team member should follow the schedule of the deceased student throughout the day in order to discuss feelings with students and staff.	No	1	52.5	2.5	25.0	20.0	100.0
A nominated ER Team member should develop a safety plan (e.g. safety plan) with each student identified as being at high risk.	No	1	50.0	0.0	37.5	12.5	100.0

The ER Team should organise for at least one telephone line to be kept free for calls to and from parents of high-risk students and external mental health professionals.	No	1	50.0	15.0	25.0	10.0	100.0
School wellbeing staff should conduct individual suicide risk assessments with students identified as being at high risk.	No	2	50.0	0.0	19.2	30.8	100.0
External mental health professionals should conduct individual suicide risk assessments with students identified as being at high risk.	No	1	47.5	5.0	40.0	7.5	100.0
A nominated ER Team member should conduct individual suicide risk assessments with students identified as being at high risk	No	1	47.5	0.0	45.0	7.5	100.0
School wellbeing staff should develop a support plan for each student identified as being at high risk.	No	2	46.2	0.0	23.1	30.8	100.0
If a student is highly distressed, a nominated ER Team member should contact their parents and send them home.	No	1	45.0	2.5	45.0	7.5	100.0
If a student expresses that they are not able to keep themselves safe, a nominated ER Team member should (as appropriate) call 000 or escort them to the emergency department.	No	1	45.0	0.0	50.0	5.0	100.0
A nominated ER Team member should ask staff to actively seek information from parents, other staff members and students regarding students who may be at high risk.	No	1	42.5	2.5	17.5	37.5	100.0
An external mental health professional should develop a safety plan (e.g. safety plan) with each student identified as being at high risk.	No	1	42.5	5.0	47.5	5.0	100.0

If a student expresses that they are not able to keep themselves safe, a nominated ER Team member should (as appropriate) call a suicide prevention helpline.	No	1	42.5	5.0	37.5	15.0	100.0
If a student is highly distressed, a nominated ER Team member should give them the option of going home.	No	2	42.3	0.0	30.8	26.9	100.0
School wellbeing staff should develop a safety plan (e.g. safety plan) with each student identified as being at high risk.	No	2	42.3	0.0	26.9	30.8	100.0
If a student expresses that they are not able to keep themselves safe, school wellbeing staff should (as appropriate call 000 or escort them to the emergency department.	No	2	42.3	0.0	57.7	0.0	100.0
A member of the school wellbeing team should provide individual counselling or support to any high-risk student.	No	2	38.5	0.0	30.8	30.8	100.0
If a student's behaviour indicates that they are an immediate danger to themselves or others a nominated ER Team member should (as appropriate) call a suicide prevention helpline.	No	1	35.0	5.0	30.0	30.0	100.0
If a student is absent from school without permission, a nominated ER Team member should contact their friends.	No	1	35.0	2.5	47.5	15.0	100.0
The ER Team should implement a systematic screening of students in order to identify those at elevated risk of suicide and/or distress. This should cover suicidal thoughts and behaviours.	No	1	32.5	0.0	27.5	40.0	100.0
The ER Team should implement a systematic screening of students in order to identify those at elevated risk of suicide and/or distress. This should cover risk factors.	No	1	32.5	0.0	27.5	40.0	100.0

The ER Team should implement a systematic screening of students in the same year level as the deceased student in order to identify those at elevated risk of suicide and/or distress. This should cover psychological distress.	No	1	32.5	0.0	30.0	37.5	100.0
An external mental health professional should develop a support plan* for each student identified as being at high risk.	No	1	32.5	2.5	60.0	5.0	100.0
If a student expresses that they are able to keep themselves safe but remains a cause for concern (e.g. showing symptoms or signs of depression, experiencing thoughts of suicide) school wellbeing staff should (as appropriate) call a suicide prevention helpline.	No	2	30.8	7.7	46.2	15.4	100.0
The ER Team should implement a systematic screening of students in order to identify those at elevated risk of suicide and/or distress. This should cover psychological distress.	No	1	30.0	0.0	30.0	40.0	100.0
The ER Team should implement a systematic screening of students in the same year level as the deceased student in order to identify those at elevated risk of suicide and/or distress. This should cover suicidal thoughts and behaviours.	No	1	30.0	0.0	32.5	37.5	100.0
The ER Team should implement a systematic screening of students in the same year level as the deceased student in order to identify those at elevated risk of suicide and/or distress. This should cover risk factors.	No	1	30.0	0.0	32.5	37.5	100.0

If a student expresses that they are able to keep themselves safe but remains a cause for concern (e.g. showing symptoms or signs of depression, experiencing thoughts of suicide) a nominated ER Team member should (as appropriate call a suicide prevention helpline.	No	1	30.0	10.0	37.5	22.5	100.0
If a student expresses that they are not able to keep themselves safe, school wellbeing staff should (as appropriate call a suicide prevention helpline.	No	2	23.1	7.7	53.8	15.4	100.0
If a student's behaviour indicates that they are an immediate danger to themselves or others school wellbeing staff should (as appropriate): call a suicide prevention helpline.	No	2	23.1	7.7	50.0	19.2	100.0
If a student expresses that they are able to keep themselves safe but remains a cause for concern (e.g. showing symptoms or signs of depression, experiencing thoughts of suicide) a nominated ER Team member should (as appropriate call 000 or escort them to the emergency department.	No	1	22.5	7.5	37.5	32.5	100.0
If a student expresses that they are able to keep themselves safe but remains a cause for concern (e.g. showing symptoms or signs of depression, experiencing thoughts of suicide) school wellbeing staff should (as appropriate) call 000 or escort them to the emergency department.	No	2	19.2	7.7	46.2	26.9	100.0
Supporting students							
Teachers who feel out of their depth should seek help from a member of the ER Team.	Yes	1	100.0	0.0	0.0	0.0	100.0
The support room should be available to any student at any time for the first few days after being informed of the death.	Yes	2	96.2	0.0	3.8	0.0	100.0

A staff member should accompany students who appear distressed to the support room or student wellbeing office if possible.	Yes	2	96.2	0.0	3.8	0.0	100.0
A nominated ER Team member should set up one or more support rooms in the school. Where possible, this should contain information about appropriate support services.	Yes	1	95.0	0.0	5.0	0.0	100.0
If during the conduct of a normal class, students ask questions, discuss or express feelings about the death, the teacher should, as appropriate acknowledge feelings and give support.	Yes	1	95.0	0.0	5.0	0.0	100.0
Teachers should not take on take on the role of being a counsellor, conducting risk assessments or diagnosing students.	Yes	1	95.0	0.0	5.0	0.0	100.0
Support room staff should provide support to distressed students.	Yes	1	92.5	0.0	7.5	0.0	100.0
If the demand for counselling exceeds the capacity of the school wellbeing team, the ER Team should arrange for external counsellors to come to the school for as long as needed.	Yes	2	92.3	0.0	7.7	0.0	100.0
If school-based counselling is not sufficient to meet a student's needs, the school wellbeing team should refer to an external mental health service.	Yes	1	90.0	0.0	10.0	0.0	100.0
The support room should be staffed at all times by a staff member known to be approachable to students.	Yes	2	88.5	0.0	7.7	3.8	100.0
The school wellbeing team should provide ongoing support to students who want it.	Yes	2	88.5	0.0	11.5	0.0	100.0

Support room staff should monitor distressed students after they leave the room to ensure that they either return to class, are collected by parents, or return to the company of supporting friends if during recess or lunch.	Yes	1	87.5	0.0	12.5	0.0	100.0
If during the conduct of a normal class, students ask questions, discuss or express feelings about the death, the teacher should, as appropriate not be judgemental, criticise, blame, do most of the talking or make promises.	Yes	1	87.5	0.0	2.5	10.0	100.0
A nominated ER Team member should set up one or more support rooms in the school. Where possible, this should provide protection from noise, bright light, and high student traffic.	Yes	1	85.0	5.0	10.0	0.0	100.0
A nominated ER Team member should set up one or more support rooms in the school. Where possible, this should contain an ongoing record (sign-in sheet) of the students who have accessed the room, which could be checked against the attendance role.	Yes	1	85.0	0.0	15.0	0.0	100.0
If any sibling of the deceased student attends the school, a nominated ER Team member should provide advice to their classmates and the teacher on how to best support the student.	Yes	1	85.0	0.0	12.5	2.5	100.0
A nominated ER Team member should set up one or more support rooms in the school. Where possible, this should be quiet and out of the way.	Yes	2	84.6	3.8	11.5	0.0	100.0
A nominated ER Team member should set up one or more support rooms in the school. Where possible, this should contain safe activities such as drawing materials, posters and games.	Yes	3	84.0	4.0	8.0	4.0	100.0

A nominated ER Team member should set up one or more support rooms in the school. Where possible, this should be set up in a small to mid-sized room.	Yes	2	80.8	7.7	11.5	0.0	100.0
The support room should be staffed at all times by a school wellbeing staff member.	Yes	2	80.8	0.0	15.4	3.8	100.0
A nominated ER Team member should set up one or more support rooms in the school. Where possible, this should contain water, tissues, fruit and tea.	Yes	1	80.0	7.5	12.5	0.0	100.0
Classroom teachers should monitor student movement to and from the support room.	Yes	1	80.0	2.5	15.0	2.5	100.0
If a bereaved student has been absent from school, a nominated ER Team member should talk to them before they return to school about how they are feeling about returning to school and what support they might need.	Yes	1	80.0	0.0	15.0	5.0	100.0
A nominated ER Team member should set up one or more support rooms in the school. Where possible, this should be set up in a small to mid-sized room.	Re-rated in round 2	1	77.5	5.0	15.0	2.5	100.0
A nominated ER Team member should set up one or more support rooms in the school. Where possible, this should be quiet and out of the way.	Re-rated in round 2	1	77.5	5.0	15.0	2.5	100.0
The support room should be available to any student at any time for the first few days after being informed of the death.	Re-rated in round 2	1	77.5	2.5	17.5	2.5	100.0
The support room should be staffed at all times by a school wellbeing staff member.	Re-rated in round 2	1	77.5	0.0	22.5	0.0	100.0

If the demand for counselling exceeds the capacity of the school wellbeing team, the ER Team should arrange for external counsellors to come to the school for as long as needed.	Re-rated in round 2	1	77.5	0.0	20.0	2.5	100.0
A nominated ER Team member should set up one or more support rooms in the school. Where possible, this should contain safe activities such as drawing materials, posters and games.	Re-rated in round 3	2	76.9	7.7	11.5	3.8	100.0
If during the conduct of a normal class, students ask questions, discuss or express feelings about the death, the teacher should, as appropriate, direct students to see a member of the school wellbeing team.	Re-rated in round 3	2	76.9	0.0	19.2	3.8	100.0
If during the conduct of a normal class, students ask questions, discuss or express feelings about the death, the teacher should, as appropriate provide accurate information about the death.	Re-rated in round 2	1	70.0	0.0	25.0	5.0	100.0
If during the conduct of a normal class, students ask questions, discuss or express feelings about the death, the teacher should, as appropriate provide opportunities to acknowledge the life of the deceased student and the loss this means for those left (discuss positive memories).	Re-rated in round 2	1	70.0	0.0	22.5	7.5	100.0
The support room should be staffed at all times by a member of staff who has been trained in suicide risk assessment.	No	2	69.2	3.8	19.2	7.7	100.0
The ER Team should not hold an assembly to discuss suicide or provide support because individual students' reactions can't be monitored.	No	1	67.5	5.0	15.0	12.5	100.0

The school wellbeing team should provide individual counselling to students who want ongoing support.	No	1	67.5	0.0	32.5	0.0	100.0
During recess and lunch breaks, nominated ER Team members or other staff members should be present at school entrances to stop students from leaving the school grounds without permission.	No	2	65.4	3.8	30.8	0.0	100.0
If during the conduct of a normal class, students ask questions, discuss or express feelings about the death, the teacher should, as appropriate provide opportunities to acknowledge the life of the deceased student and the loss this means for those left (discuss positive memories).	No	2	65.4	3.8	23.1	7.7	100.0
If during the conduct of a normal class, students ask questions, discuss or express feelings about the death, the teacher should, as appropriate sit quietly with students while they talk, cry, or remain silent.	No	1	65.0	0.0	20.0	15.0	100.0
If during the conduct of a normal class, students ask questions, discuss or express feelings about the death, the teacher should, as appropriate reassure students that no-one is to blame for the death.	No	1	65.0	0.0	22.5	12.5	100.0
214. A staff member should accompany students who appear distressed to the support room or student wellbeing office.	No	1	62.5	2.5	35.0	0.0	100.0
Support room staff should redirect students who are not distressed back to class.	No	1	60.0	5.0	25.0	10.0	100.0
If during the conduct of a normal class, students ask questions, discuss, or express feelings about the death, the teacher should, as appropriate, direct	No	3	60.0	4.0	16.0	20.0	100.0

students to see a member of the school wellbeing team.							
If after a discussion of the above, a class still has difficulty concentrating, the teacher should, as appropriate lead classroom discussions that focus on helping students to cope with the loss.	No	1	55.0	0.0	30.0	15.0	100.0
If after a discussion of the above, a class still has difficulty concentrating, the teacher should, as appropriate set work for those who wish to resume normal lessons, but allow others to speak quietly with each other or write in a journal, or simply sit and think.	No	1	55.0	0.0	37.5	7.5	100.0
If during the conduct of a normal class, students ask questions, discuss or express feelings about the death, the teacher should, as appropriate provide accurate information about the death.	No	2	53.8	0.0	30.8	15.4	100.0
If after a discussion of the above, a class still has difficulty concentrating, the teacher should, as appropriate provide activities to help students cope with their feelings, such as artwork, music, and writing.	No	1	52.5	0.0	37.5	10.0	100.0
Classroom discussions on grief and coping with a sudden loss should be facilitated by a member of the school wellbeing team.	No	1	50.0	0.0	40.0	10.0	100.0
Classroom discussions on grief and coping with a sudden loss should be facilitated by a mental health professional from an external service.	No	1	50.0	0.0	40.0	10.0	100.0
The door of the support room should be left ajar at all times.	No	1	42.5	25.0	32.5	0.0	100.0

Classroom teachers should allow students to leave class on their own initiative to access the support room.	No	1	42.5	0.0	30.0	27.5	100.0
Another student should accompany students who appear distressed to the support room or student wellbeing office.	No	1	42.5	0.0	32.5	25.0	100.0
The school wellbeing team should provide group counselling to students who want ongoing support.	No	1	40.0	0.0	40.0	20.0	100.0
The support room should have clearly stated rules about who can use the room, when and for what length of time.	No	1	37.5	10.0	30.0	22.5	100.0
If a school has a peer counselling program, the peer counsellors should not be used to support other students following the suicide.	No	1	35.0	0.0	50.0	15.0	100.0
Librarians should present literature on stress, coping, and communication skills in a media display in the library.	No	1	35.0	7.5	40.0	17.5	100.0
A nominated ER Team member should organise classroom discussions on grief and coping with a sudden loss.	No	1	35.0	0.0	47.5	17.5	100.0
During school hours, a nominated ER Team member should monitor local places where the deceased student's peer group gather off school grounds (e.g. parks, fast food chains) to make contact with any students needing assistance.	No	1	32.5	7.5	37.5	22.5	100.0
If after a discussion of the above, a class still has difficulty concentrating, the teacher should, as appropriate teach problem-solving skills.	No	1	32.5	5.0	42.5	20.0	100.0
The ER Team should arrange for an external service to provide suicide bereavement support groups within the school.	No	1	27.5	2.5	57.5	12.5	100.0

During school hours, nominated ER Team members or other staff members should be present at school entrances to stop students from leaving the school grounds without permission.	No	1	27.5	10.0	47.5	15.0	100.0
Classroom discussions on grief and coping with a sudden loss should be facilitated by a trained classroom teacher.	No	1	27.5	2.5	47.5	22.5	100.0
The support room should be staffed at all times by a mental health professional from an external service.	No	1	25.0	5.0	62.5	7.5	100.0
A nominated ER Team member should organise for a member of staff to offer stress reduction sessions.	No	1	22.5	7.5	47.5	22.5	100.0
If during the conduct of a normal class, students ask questions, discuss or express feelings about the death, the teacher should, as appropriate talk about how suicide does not end pain but takes the pain from the person who died by suicide and passes it on to the whole community, which now has to live with the grief.	No	1	20.0	0.0	20.0	60.0	100.0
If students ask how they can help the deceased's family, teachers should give suggestions (e.g. offer to mow the lawn or go shopping).	No	1	17.5	2.5	32.5	47.5	100.0
If a school has a peer counselling program, the peer counsellors should be available to support other students following the suicide.	No	1	17.5	0.0	45.0	37.5	100.0
The ER Team should hold an assembly to discuss suicide or provide support.	No	1	15.0	0.0	20.0	65.0	100.0
Supporting staff							
A nominated ER Team member should encourage staff to seek appropriate support where needed (this may be internal or external).	Yes	2	100.0	0.0	0.0	0.0	100.0

The designated leader of the ER Team should encourage other ER Team members to seek appropriate support where needed (this may be internal or external).	Yes	2	96.2	0.0	3.8	0.0	100.0
The designated leader of the ER Team should ensure that the ER Team members have ongoing opportunities to discuss their feelings and receive support.	Yes	1	92.5	0.0	2.5	5.0	100.0
The principal or his/her designee will check in periodically with staff to ensure that everyone is supported as much as is feasible.	Yes	1	90.0	0.0	5.0	5.0	100.0
A nominated ER Team member should organise on-site counselling in an appropriate location for staff who want it.	Yes	2	88.5	0.0	7.7	3.8	100.0
The ER Team should compile a list of all staff members who had contact with the deceased student and check if they need extra support.	Yes	1	87.5	0.0	7.5	5.0	100.0
The ER Team should prepare a list of all staff members at high risk of distress and check if they need extra support.	Yes	1	82.5	0.0	12.5	5.0	100.0
If the principal or other senior staff need additional support with their roles, they should contact the relevant education department to request the appointment of an additional interim school leader.	Yes	1	82.5	2.5	10.0	5.0	100.0
A nominated ER Team member should encourage staff to seek external counselling resources.	Yes	1	80.0	0.0	20.0	0.0	100.0
A nominated ER Team member should arrange for several substitute teachers to be on hand to provide rotating coverage in case teachers need to take time out of their classrooms, or to attend the funeral.	Yes	1	80.0	5.0	10.0	5.0	100.0

A nominated ER Team member should organise on-site counselling in an appropriate location for staff who want it.	Re-rated in round 2	1	77.5	2.5	17.5	2.5	100.0
A nominated ER Team member should organise educational workshops around stress-management and self-care.	Re-rated in round 2	1	77.5	5.0	15.0	2.5	100.0
A nominated ER Team member should organise educational workshops around stress-management and self-care.	No	2	76.9	0.0	23.1	0.0	100.0
The designated leader of the ER Team should encourage other team members to seek external counselling resources.	Re-rated in round 2	1	75.0	0.0	22.5	2.5	100.0
A nominated ER Team member should encourage staff to use on-site counselling resources.	Re-rated in round 2	1	70.0	2.5	20.0	7.5	100.0
A nominated ER Team member should inform all staff that members of the ER Team will be available for ongoing support in the months following the death if the need arises.	Re-rated in round 2	1	70.0	2.5	17.5	10.0	100.0
The designated leader of the ER Team should encourage other team members to use on-site counselling resources.	No	1	67.5	2.5	20.0	10.0	100.0
A nominated ER Team member should organise for the provision of food, tissues and drinks to support staff, including members of the ER Team.	No	1	67.5	12.5	10.0	10.0	100.0
A nominated ER Team member should encourage staff to use on site counselling resources.	No	2	65.4	3.8	15.4	15.4	100.0
The designated leader of the ER Team should encourage other ER Team members to seek external counselling resources.	No	2	61.5	0.0	19.2	19.2	100.0

A nominated ER Team member should organise on-site support groups (e.g. about grief and loss) held after school with a mental health professional if a number of staff need more support.	No	1	60.0	5.0	30.0	5.0	100.0
A nominated ER Team member should inform all staff that the ER Team is not a counselling or bereavement service, and support should be sought elsewhere (i.e. from school wellbeing team or EAP).	No	2	57.7	3.8	26.9	11.5	100.0
A nominated ER Team member should organise social activities for staff where they can connect in a positive way and support each other (e.g. dinner, sports game, movie night, morning tea).	No	1	40.0	15.0	37.5	7.5	100.0
A nominated ER Team member should organise on-site exercise, walking, meditation groups, or other stress management activities.	No	1	40.0	12.5	37.5	10.0	100.0
A nominated ER Team member should inform all staff that members of the ER Team will be available for ongoing support in the months following the death if the need arises.	No	2	38.5	0.0	34.6	26.9	100.0
The deceased student’s belongings							
A nominated ER Team member should secure the student's locker as soon as possible, to preserve the contents for the family or police as appropriate.	Yes	1	97.5	0.0	2.5	0.0	100.0
A nominated ER Team member should ensure that the name of the deceased student (and parents where appropriate) is removed from any school mailing lists, individual class rosters etc.	Yes	1	97.5	0.0	2.5	0.0	100.0
A nominated ER Team member should talk to the deceased student’s family to determine a mutually	Yes	1	95.0	0.0	2.5	2.5	100.0

agreeable date and time in private, to return the belongings.							
If the family collects the belongings from the school, a nominated ER Team member should be present to assist and provide support if necessary.	Yes	1	92.5	0.0	5.0	2.5	100.0
A nominated ER Team member should arrange for the deceased student’s belongings to be collected and stored safely in a respectful container (not a rubbish bag).	Yes	1	87.5	0.0	12.5	0.0	100.0
Before emptying the locker a nominated ER Team member should ensure that they have approval from the family and/or police.	Yes	1	85.0	5.0	7.5	2.5	100.0
A nominated ER Team member or family should empty the deceased student's locker when other students are not present.	Yes	1	85.0	0.0	10.0	5.0	100.0
If there is an empty chair/desk in the classroom which would normally have been occupied by the deceased student, the teacher should discuss with the ER Team what will be done with the seat/desk and inform the class.	Yes	2	80.8	0.0	19.2	0.0	100.0
If there is an empty chair/desk in the classroom which would normally have been occupied by the deceased student, the teacher should discuss with the ER Team what will be done with the seat/desk and inform the class.	Re-rated in round 2	1	75.0	5.0	15.0	5.0	100.0
A nominated ER Team member should make an inventory of the deceased student's belongings.	Re-rated in round 2	1	72.5	7.5	12.5	7.5	100.0

Before emptying the locker a nominated ER Team member should contact the family, and ask if they wish to do it themselves.	No	1	62.5	5.0	17.5	15.0	100.0
Before emptying the locker a nominated ER Team member should inform close friends of the deceased student that it will be emptied.	No	1	62.5	5.0	25.0	7.5	100.0
If there is an empty chair/desk in the classroom which would normally have been occupied by the deceased student, the teacher should acknowledge the deceased student's absence and allow students to express their feelings.	No	1	60.0	2.5	20.0	17.5	100.0
A nominated ER Team member should make an inventory of the deceased student's belongings.	No	2	57.7	7.7	30.8	3.8	100.0
If there is an empty chair/desk in the classroom which would normally have been occupied by the deceased student, the teacher should discuss with the class what to do with the empty seat/desk in the future (e.g. leave it unoccupied for the rest of the year or rearrange the seating).	No	1	52.5	0.0	32.5	15.0	100.0
A nominated ER Team member should make an inventory of the deceased student's belongings, only if requested to do so by the police.	No	2	23.1	0.0	34.6	42.3	100.0
Funeral and memorial							
A nominated ER Team member should ensure the policy for releasing students during school hours to attend is conveyed.	Yes	1	100.0	0.0	0.0	0.0	100.0
If the school does hold a memorial service it should not glorify the death.	Yes	1	97.5	0.0	0.0	2.5	100.0

If the school does hold a memorial service the ER Team should monitor student reactions and follow up with any students who appear distressed.	Yes	1	97.5	0.0	0.0	2.5	100.0
A nominated ER Team member should ensure that the family's wishes regarding staff and student attendance at and participation in the funeral or memorial service is conveyed.	Yes	1	97.5	0.0	2.5	0.0	100.0
The ER Team should ensure that any memorial sites or activities do not glorify, vilify or stigmatise the deceased student or their death.	Yes	1	95.0	0.0	0.0	5.0	100.0
If the school does hold a memorial service the ER Team should inform the family of the deceased student that the memorial service will be occurring.	Yes	1	92.5	0.0	5.0	2.5	100.0
A nominated ER Team member should ensure the location and time of the funeral or memorial service is conveyed.	Yes	1	92.5	0.0	7.5	0.0	100.0
The ER Team should plan ahead for anniversaries and other significant dates or events (e.g. graduation, school formal) by reminding staff, students and parents about support services available to them.	Yes	1	92.5	0.0	5.0	2.5	100.0
The ER Team should plan ahead for anniversaries and other significant dates or events (e.g. graduation, school formal) by increasing efforts to monitor high-risk students and staff.	Yes	1	92.5	0.0	7.5	0.0	100.0
A nominated ER Team member should ensure that any memorial messages or objects given to the family are appropriate.	Yes	2	92.3	0.0	7.7	0.0	100.0
A nominated ER Team member should offer memorial objects to the family at an appropriate time.	Yes	2	92.3	0.0	7.7	0.0	100.0

If there is a yearbook or equivalent, the level of content (if any) dedicated to the deceased student should be consistent with previous deaths from other causes.	Yes	2	92.3	0.0	7.7	0.0	100.0
A nominated ER Team member should monitor any memorial site on school grounds for objects or messages that are inappropriate (hostile or inflammatory) and remove them.	Yes	1	90.0	0.0	5.0	5.0	100.0
If the school does hold a memorial service it should take the form of any other memorial service for a student death.	Yes	1	90.0	2.5	5.0	2.5	100.0
A nominated ER Team member should ensure that information about what to expect at the funeral or memorial (e.g. whether there will be an open casket) is conveyed.	Yes	1	90.0	0.0	7.5	2.5	100.0
If the funeral or memorial service is held during school hours, the ER Team should allow students to attend only with appropriate parental permission.	Yes	2	88.5	0.0	7.7	3.8	100.0
The school should avoid allowing the funeral or memorial service to be held on school grounds, unless this is consistent with what has been done for other deaths or appropriate given the school's faith.	Yes	2	88.5	0.0	11.5	0.0	100.0
If a page of the yearbook is dedicated to the deceased student the ER Team should approve all content.	Yes	2	88.5	0.0	11.5	0.0	100.0
The ER Team should plan if and how the deceased student will be honoured at any graduation.	Yes	2	88.5	0.0	7.7	3.8	100.0
If a memorial site is spontaneously created on school grounds, the ER Team should decide if it is in a suitable location and, if not, enlist key students to help move items in a respectful way to a “designated” memorial site.	Yes	3	88.0	0.0	8.0	4.0	100.0

A nominated ER Team member should monitor any memorial site on school grounds for messages that indicate students who may be at high risk.	Yes	1	87.5	0.0	7.5	5.0	100.0
A nominated ER Team member should ensure that any acknowledgment of the death or coverage of memorial activities in a school newsletter follows national guidelines for the reporting of suicide in the media.	Yes	1	87.5	0.0	10.0	2.5	100.0
A nominated ER Team member should ensure the guidance regarding how to express condolences to the family is conveyed.	Yes	1	87.5	0.0	10.0	2.5	100.0
The ER Team should ensure that support is available to students before and after the funeral or memorial service, especially for those who have played a role in the proceedings (e.g. as a pall bearer or speaker).	Yes	1	87.5	0.0	12.5	0.0	100.0
School staff should be aware that any memorial sites or activities could be distressing to others or seen by vulnerable students as a way to receive recognition for suicide.	Yes	1	85.0	0.0	10.0	5.0	100.0
If the school becomes aware that students have established a memorial off school grounds, a nominated ER Team member should inform the police if they are concerned about the safety of students congregating near any memorial site (e.g. if the memorial is near a railway line).	Yes	1	85.0	0.0	15.0	0.0	100.0
After speaking with the family of the deceased student, a nominated ER Team member should communicate with the person running the funeral or memorial service (e.g. funeral director, religious leader) about any materials that the school could provide to staff and students to help them prepare for the service.	Yes	1	85.0	0.0	10.0	5.0	100.0

If the funeral or memorial service is held during school hours, the ER Team should not require all students to attend the funeral or memorial service.	Yes	1	85.0	0.0	7.5	7.5	100.0
If the funeral or memorial service is held during school hours, the Emergency Response Team should keep to the school's regular routine.	Yes	1	85.0	2.5	10.0	2.5	100.0
If a page of the yearbook is dedicated to the deceased student it should be consistent with what would be dedicated to any deceased student, regardless of their popularity or cause of death.	Yes	1	85.0	2.5	10.0	2.5	100.0
The ER Team should ensure that any memorial sites or activities are the same as they would be for a non-suicide death.	Yes	1	82.5	0.0	10.0	7.5	100.0
If the funeral or memorial service is held during school hours, the Emergency Response Team should ensure that students who do not attend the funeral or memorial service attend classes as usual.	Yes	1	82.5	0.0	15.0	2.5	100.0
A nominated ER Team member should discuss with the family if/how they would like their son or daughter acknowledged in any graduation or award ceremonies.	Yes	1	82.5	0.0	12.5	5.0	100.0
After speaking with the family of the deceased student, a nominated ER Team member should communicate with the person running the funeral or memorial service (e.g. funeral director, religious leader) about the route of any funeral procession (i.e., avoid going by the school, if possible).	Yes	2	80.8	0.0	15.4	3.8	100.0
If a page of the yearbook is dedicated to the deceased student the deceased student's family should approve all content.	Yes	2	80.8	3.8	7.7	7.7	100.0

The ER Team should inform staff about appropriate ways of grieving and honouring the deceased student.	Yes	1	80.0	0.0	10.0	10.0	100.0
A nominated ER Team member should sensitively explain to students the purpose of a memorial site or activity and the rationale for permitting certain kinds of memorials and not others.	Yes	1	80.0	0.0	10.0	10.0	100.0
The ER Team should ensure that any memorial site or activity is culturally appropriate.	Yes	1	80.0	0.0	10.0	10.0	100.0
After speaking with the family of the deceased student, a nominated ER Team member should communicate with the person running the funeral or memorial service (e.g. funeral director, religious leader) about student attendance at the service.	Yes	1	80.0	2.5	12.5	5.0	100.0
After speaking with the family of the deceased student, a nominated ER Team member should communicate with the person running the funeral or memorial service (e.g. funeral director, religious leader)<br>about available resources pertaining to how to talk to young people about suicide (e.g. How to talk about suicide with young people).	Yes	1	80.0	5.0	10.0	5.0	100.0
One or more members or the ER Team should attend the funeral or memorial service to supervise and support those attending.	Yes	1	80.0	0.0	15.0	5.0	100.0
If appropriate, a nominated ER Team member should inform police about any safety concerns regarding the funeral or memorial service.	Yes	1	80.0	2.5	15.0	2.5	100.0
If the school does hold a memorial service, it should be open to the whole school but attendance should be optional.	Yes	3	80.0	0.0	8.0	12.0	100.0

After speaking with the family of the deceased student, a nominated ER Team member should communicate with the person running the funeral or memorial service (e.g. funeral director, religious leader) about mental health services available for students.	Re-rated in round 2	1	77.5	5.0	10.0	7.5	100.0
After speaking with the family of the deceased student, a nominated ER Team member should communicate with the person running the funeral or memorial service (e.g. funeral director, religious leader)<br>about the route of any funeral procession (i.e., avoid going by the school, if possible).	Re-rated in round 2	1	77.5	2.5	12.5	7.5	100.0
If the funeral or memorial service<br>is held during school hours, the Emergency Response Team should allow students to attend only with appropriate parental permission.	Re-rated in round 2	1	77.5	0.0	17.5	5.0	100.0
A nominated ER Team member should make a list of students who attend the funeral or memorial service.	Re-rated in round 2	1	77.5	0.0	17.5	5.0	100.0
If a page of the yearbook is<br>dedicated to the deceased student it should not mention details of the death.	Re-rated in round 2	1	77.5	0.0	17.5	5.0	100.0
The ER Team should plan if and how the deceased student will be honoured at any graduation	Re-rated in round 2	1	77.5	0.0	17.5	5.0	100.0

If a memorial site is spontaneously created on school grounds, the ER Team should decide if it is in a suitable location and, if not enlist key students to help move items in a respectful way to a "designated" memorial site (e.g. a table, banner for students to write messages on) at the end of each day and place a note telling students where to find them.	Re-rated in round 3	2	76.9	0.0	11.5	11.5	100.0
If the school does hold a memorial service, it should be open to the whole school but attendance should be optional.	Re-rated in round 3	2	76.9	3.8	19.2	0.0	100.0
After speaking with the family of the deceased student, a nominated ER Team member should communicate with the person running the funeral or memorial service (e.g. funeral director, religious leader) about arrangements for the service.	No	2	76.9	0.0	19.2	3.8	100.0
If the funeral or memorial service is held during school hours, the ER Team should encourage students to attend with their parents.	No	2	76.9	0.0	19.2	3.8	100.0
A nominated ER Team member should offer memorial objects to the family at an appropriate time.	Re-rated in round 2	1	75.0	0.0	20.0	5.0	100.0
If the funeral or memorial service is held during school hours, the Emergency Response Team should encourage students to attend with their parents.	Re-rated in round 2	1	75.0	2.5	17.5	5.0	100.0
After speaking with the family of the deceased student, a nominated ER Team member should communicate with the person running the funeral or memorial service (e.g. funeral director, religious leader) about mental health services available for students.	No	2	73.1	3.8	19.2	3.8	100.0

A nominated ER Team member should discuss the type and timing of any memorial site or activity with the close friends of the deceased student.	Re-rated in round 2	1	72.5	0.0	17.5	10.0	100.0
A nominated ER Team member should ensure that any memorial messages or objects given to the family are appropriate.	Re-rated in round 2	1	72.5	0.0	22.5	5.0	100.0
If a page of the yearbook is dedicated to the deceased student the ER Team should approve all content.	Re-rated in round 2	1	72.5	2.5	22.5	2.5	100.0
If a page of the yearbook is dedicated to the deceased student the student's family should approve all content.	Re-rated in round 2	1	72.5	5.0	22.5	0.0	100.0
If a memorial site is spontaneously created on school grounds, the ER Team should decide if it is in a suitable location and, if not, move items to a “designated” memorial site (e.g. a table, or a banner for students to write messages on) at the end of each day and place a note telling students where to find them.	Re-rated in round 2	1	70.0	0.0	25.0	5.0	100.0
After speaking with the family of the deceased student, a nominated ER Team member should communicate with the person running the funeral or memorial service (e.g. funeral director, religious leader) about arrangements for the service.	Re-rated in round 2	1	70.0	0.0	17.5	12.5	100.0
The school should hold a memorial service if this is consistent with what has been done for previous deaths due to other causes.	No	2	69.2	0.0	26.9	3.8	100.0
A nominated ER Team member should discuss the type and timing of any memorial site or activity with the close friends of the deceased student.	No	2	69.2	0.0	11.5	19.2	100.0

A nominated ER Team member should make a list of students who attend the funeral or memorial service.	No	2	69.2	3.8	7.7	19.2	100.0
If a page of the yearbook is dedicated to the deceased student it should not mention details of the death.	No	2	69.2	0.0	19.2	11.5	100.0
If a designated memorial site is to be set up, staff should discuss with students the form it should take.	No	1	67.5	0.0	17.5	15.0	100.0
If a designated memorial site is to be set up, staff should discuss with students where it should be placed.	No	1	67.5	0.0	17.5	15.0	100.0
After speaking with the family of the deceased student, a nominated ER Team member should communicate with the person running the funeral or memorial service (e.g. funeral director, religious leader) about any security arrangements.	No	1	67.5	5.0	17.5	10.0	100.0
The ER Team should plan ahead for anniversaries and other significant dates or events (e.g. graduation, school formal) by reminding proactively organising wellbeing activities.	No	1	67.5	2.5	17.5	12.5	100.0
There should be a memorial site or activity if this is consistent with what has been done for previous deaths due to other causes.	No	2	65.4	0.0	26.9	7.7	100.0
If a designated memorial site is to be set up, staff should discuss with students how long it will remain in place.	No	1	65.0	0.0	17.5	17.5	100.0
If a designated memorial site is to be set up, staff should discuss with students that its purpose is to allow students to express their grief, and give comfort to the family.	No	1	65.0	0.0	22.5	12.5	100.0

If the school becomes aware that students have established a memorial off school grounds, a nominated Emergency Response Team member should sensitively remind students about the purpose of a memorial (e.g. to acknowledge the deceased student, allow others to express grief), and the rationale for permitting certain kinds of activities and not others.	No	1	65.0	0.0	30.0	5.0	100.0
If the school does hold a memorial service the ER Team should inform parents of those students who will be attending.	No	1	65.0	2.5	22.5	10.0	100.0
If a page of the yearbook is dedicated to the deceased student it is acceptable to include basic details about the student (e.g. a photograph, their name, birth and death dates, a sentence or two about what the individual did while living, messages from friends, loved ones).	No	1	62.5	0.0	25.0	12.5	100.0
If a memorial site is spontaneously created on school grounds, the ER Team should decide if it is in a suitable location and, if not move items to a "designated" memorial site (e.g. a table, or a banner for students to write messages on) at the end of each day and place a note telling students where to find them.	No	2	57.7	0.0	19.2	23.1	100.0
The ER Team should allow collaboration with an external mental health service to develop and implement a curriculum focused on how to solve personal problems effectively.	No	1	57.5	2.5	35.0	5.0	100.0
If the school does hold a memorial service the ER Team should seek permission from parents of those students who may be attending.	No	1	57.5	2.5	27.5	12.5	100.0

The ER Team should allow books to be purchased on mental health for the school or local library.	No	1	55.0	2.5	37.5	5.0	100.0
Staff should encourage students and other staff members to bring cards and other items they would like to share with the family to the main office or other designated location.	No	1	55.0	10.0	25.0	10.0	100.0
A nominated ER Team member should inform all staff, students and parents about any arrangements for the funeral or memorial service.	No	1	55.0	0.0	27.5	17.5	100.0
With the family's permission, the ER Team should encourage the principal to attend the funeral or memorial service.	No	1	55.0	5.0	25.0	15.0	100.0
The ER Team should not allow large student assemblies about the deceased student or a moment of silence at assemblies.	No	1	52.5	0.0	42.5	5.0	100.0
The ER Team should not allow dedication of songs, sports events or other events.	No	1	50.0	0.0	35.0	15.0	100.0
The ER Team should not allow students to wear t-shirts and badges bearing images of the deceased student.	No	1	50.0	5.0	30.0	15.0	100.0
If the funeral or memorial service is held during school hours, the ER Team should not use school buses to transport students to and from the funeral or memorial service.	No	1	50.0	7.5	40.0	2.5	100.0
With the family's permission, the ER Team should encourage those staff members who knew the student well to attend the funeral or memorial service.	No	1	50.0	10.0	22.5	17.5	100.0

With the family's permission, the ER Team should encourage the school wellbeing staff to attend the funeral or memorial service.	No	1	50.0	10.0	25.0	15.0	100.0
The ER Team should allow the sponsorship of a mental health awareness day.	No	1	47.5	5.0	40.0	7.5	100.0
If the school becomes aware that students have established a memorial off school grounds, a nominated Emergency Response Team member should explain to the students that the memorial should be in place for a limited time.	No	1	47.5	0.0	37.5	15.0	100.0
If the school becomes aware that students have established a memorial off school grounds, a nominated ER Team member should suggest that the students participate in a (supervised) ceremony to disassemble the memorial.	No	1	47.5	0.0	37.5	15.0	100.0
With the family's permission, the ER Team should encourage the school leadership team to attend the funeral or memorial service.	No	1	47.5	5.0	27.5	20.0	100.0
The ER Team should allow a team to participate in an awareness or fundraising event sponsored by one of the national mental health or suicide prevention organisations.	No	1	45.0	0.0	47.5	7.5	100.0
The school should not allow the funeral or memorial service to be held on school grounds.	No	1	45.0	2.5	42.5	10.0	100.0
If there is no dedicated page in the yearbook, schools should include a brief statement acknowledging and naming the deceased student.	No	1	42.5	0.0	52.5	5.0	100.0
If a page of the yearbook is dedicated to the deceased student whenever	No	1	42.5	0.0	22.5	35.0	100.0

possible, the focus should be on mental health and/or suicide prevention.							
The ER Team should allow students to create artwork that express something of students’ own grief experience.	No	1	40.0	5.0	42.5	12.5	100.0
With the family's permission, the ER Team should encourage religious leaders (e.g. chaplain, school priest) to attend the funeral or memorial service.	No	1	40.0	12.5	32.5	15.0	100.0
If there is a yearbook or equivalent there should not be a specific page dedicated to the deceased student.	No	1	40.0	0.0	42.5	17.5	100.0
The ER Team should allow the school to hold a fundraising event to support a local crisis helpline or other suicide prevention program.	No	1	37.5	0.0	50.0	12.5	100.0
The ER Team should allow a book to be made, which is available in the school office for several weeks in which students can write messages to the family, share memories of the deceased student, or offer condolences.	No	1	37.5	0.0	52.5	10.0	100.0
The ER Team should not allow any flag to be flown at half-mast.	No	1	32.5	2.5	50.0	15.0	100.0
The ER Team should not allow permanent memorials of any kind (e.g. at the student’s locker, plaques, trees, benches, retirement of a sports jersey, shrines of any kind).	No	1	30.0	5.0	52.5	12.5	100.0
The ER Team should set up a designated memorial site if this is requested by students or staff.	No	1	30.0	0.0	47.5	22.5	100.0
If the school does hold a memorial service it should be conducted with a small group (e.g. student's year level or close friends).	No	1	30.0	0.0	52.5	17.5	100.0

A nominated ER Team member should inform only those staff, students and parents who ask about any arrangements for the funeral or memorial service.	No	1	30.0	0.0	32.5	37.5	100.0
With the family's permission, the ER Team should encourage any external mental health professionals involved with the school to attend the funeral or memorial service.	No	1	27.5	15.0	40.0	17.5	100.0
The ER Team should plan ahead for anniversaries and other significant dates or events (e.g. graduation, school formal) by reminding proactively organising a commemorative event.	No	1	27.5	10.0	30.0	32.5	100.0
The school should hold a memorial service for the deceased student, if requested by friends and family.	No	1	25.0	0.0	57.5	17.5	100.0
The ER Team should leave the designated memorial site in place until after the funeral.	No	1	22.5	5.0	47.5	25.0	100.0
If a memorial site is spontaneously created on school grounds, the ER Team should remove items as soon as possible and store them in a secure location.	No	1	22.5	2.5	47.5	27.5	100.0
The school should not hold a memorial service for the deceased student.	No	1	20.0	0.0	65.0	15.0	100.0
There should not be any memorial site on school grounds.	No	2	19.2	0.0	46.2	34.6	100.0
The school should not hold a memorial service, either on or off school grounds.	No	2	15.4	0.0	50.0	34.6	100.0
The ER Team should leave the designated memorial site in place until students stop using it.	No	1	15.0	2.5	50.0	32.5	100.0
The ER Team should allow the creation of a school-based community service program in honour of the deceased student.	No	1	12.5	0.0	50.0	37.5	100.0

The ER Team should allow the school to raise funds to help the family with their funeral expenses.	No	1	12.5	7.5	45.0	35.0	100.0
The ER Team should allow the school to hold a day of community service.	No	1	10.0	2.5	60.0	27.5	100.0
A nominated ER Team member should encourage the family of the deceased student to schedule the funeral or memorial service after school hours to facilitate the attendance of students.	No	1	10.0	15.0	27.5	47.5	100.0
If the school does hold a memorial service it should be a whole school service.	No	1	7.5	5.0	52.5	35.0	100.0
The ER Team should leave the designated memorial site in place indefinitely.	No	1	5.0	0.0	45.0	50.0	100.0
If there is a yearbook or equivalent there should be a specific page dedicated to the deceased student.	No	1	5.0	0.0	35.0	60.0	100.0
The ER Team should leave the designated memorial site in place for 1 week.	No	1	0.0	5.0	57.5	37.5	100.0
The ER Team should leave the designated memorial site in place for 2 weeks.	No	1	0.0	5.0	55.0	40.0	100.0
The school should hold a memorial service for the deceased student.	No	1	0.0	0.0	65.0	35.0	100.0
Internet and social media							
The ER Team should inform students that they should talk to a trusted adult about any inappropriate or concerning comments.	Yes	1	100.0	0.0	0.0	0.0	100.0
The ER Team should follow the guidelines for responsible reporting of suicide (e.g. Mindframe Guidelines) when communicating about the death using the internet or social media.	Yes	1	95.0	0.0	5.0	0.0	100.0

A nominated ER Team member should contact administrators of social media groups or pages that honour the deceased student, and ask them to ensure that the page includes safe messaging and accurate information.	Yes	2	92.3	0.0	3.8	3.8	100.0
A nominated ER Team member should contact administrators of social media groups or pages that honour the deceased student, and ask them to avoid material that may glorify, vilify, or stigmatise the deceased student or their death.	Yes	2	92.3	0.0	3.8	3.8	100.0
A nominated ER Team member should contact administrators of social media groups or pages that honour the deceased student, and ask them to report any concerning comments made by the school's students to a nominated ER Team member.	Yes	2	92.3	0.0	7.7	0.0	100.0
If the school has a website and/or social media presence, the ER Team should use these to proactively communicate with students, teachers, and parents about external mental health and crisis services (e.g. Kids Helpline).	Yes	1	87.5	0.0	12.5	0.0	100.0
If the school has a website and/or social media presence, the ER Team should use these to proactively communicate with students, teachers, and parents about sources of help within the school.	Yes	1	85.0	0.0	15.0	0.0	100.0
If the school has a website and/or social media presence, the ER Team should use these to proactively communicate with students, teachers, and parents about organisations that promote positive mental health and wellbeing (e.g. ReachOut.com, eheadspace, Beyondblue).	Yes	1	85.0	0.0	15.0	0.0	100.0
The ER Team should advise school staff not to engage in online discussions about the death.	Yes	1	85.0	0.0	15.0	0.0	100.0

The ER Team should remind students about the possible risks of online memorials (e.g. comments may become public without their permission, online memorials can attract negative & hurtful comment, anything that romanticises suicide can be harmful to students at risk)	Yes	1	85.0	0.0	12.5	2.5	100.0
If required, a nominated ER Team member should contact parents of students indicating that they may be at risk.	Yes	1	85.0	0.0	10.0	5.0	100.0
If required, a nominated ER Team member should contact emergency services and/or parents when information posted online may provoke anti-social behaviour in the community or indicate risk to a person's safety.	Yes	2	84.6	0.0	15.4	0.0	100.0
If required, a nominated ER Team member should dispel rumours (spread through social media).	Yes	1	82.5	0.0	10.0	7.5	100.0
The ER Team should advise parents to monitor their children's use of social media for inappropriate or concerning comments.	Yes	1	82.5	0.0	12.5	5.0	100.0
The ER Team should provide parents with links to suicide prevention or mental health information to post on their own social media pages.	Yes	1	82.5	2.5	10.0	5.0	100.0
A nominated ER Team member should contact administrators of social media groups or pages that honour the deceased student, and ask them to monitor and remove any inappropriate (e.g. hostile or inflammatory) comments.	Yes	2	80.8	0.0	15.4	3.8	100.0
The ER Team should inform students that school staff are only interested in supporting a healthy response to their peer's death, not in thwarting communication.	Yes	2	80.8	0.0	7.7	11.5	100.0

A nominated ER Team member should instruct staff not to post any content on social media about the death.	Yes	2	80.8	0.0	19.2	0.0	100.0
The ER Team should monitor student use of social media (e.g. for rumours, information about upcoming or impromptu gatherings, derogatory messages about the deceased student, messages victimising or bullying current students).	Yes	2	80.8	0.0	15.4	3.8	100.0
A nominated ER Team member should advise staff to consult with them before posting any comments about the death on social media.	Yes	1	80.0	0.0	7.5	12.5	100.0
A nominated ER Team member should provide these students (who have access to the deceased student's sites) with advice and support.	Yes	1	80.0	0.0	20.0	0.0	100.0
If required, a nominated ER Team member should speak directly with students, parents and staff who have posted inappropriate comments.	Yes	1	80.0	0.0	15.0	5.0	100.0
If required, a nominated ER Team member should contact emergency services and/or parents when information posted online may provoke anti-social behaviour in the community or indicate risk to a person’s safety.	Re-rated in round 2	1	77.5	2.5	15.0	5.0	100.0
A nominated ER Team member should provide the family with information and resources for dealing with social media profiles held by the deceased.	Re-rated in round 3	2	76.9	3.8	19.2	0.0	100.0
A nominated ER Team member should provide the family with information and resources for dealing with social media profiles held by the deceased.	No	3	76.0	4.0	16.0	4.0	100.0

The ER Team should monitor student use of social media (e.g. for rumours, information about upcoming or impromptu gatherings, derogatory messages about the deceased student, messages victimising or bullying current students).	Re-rated in round 2	1	75.0	0.0	22.5	2.5	100.0
A nominated ER Team member should contact administrators of social media groups or pages that honour the deceased student, and ask them to ensure that the page includes safe messaging and accurate information.	Re-rated in round 2	1	75.0	0.0	20.0	5.0	100.0
A nominated ER Team member should contact administrators of social media groups or pages that honour the deceased student, and ask them to avoid material that may glorify, vilify, or stigmatise the deceased student or their death.	Re-rated in round 2	1	75.0	0.0	20.0	5.0	100.0
If required, a nominated ER Team member should contact the social media organisation directly to request they remove inappropriate messages or pages.	No	2	73.1	0.0	26.9	0.0	100.0
The ER Team should inform students that school staff are only interested in supporting a healthy response to their peer's death, not in thwarting communication.	Re-rated in round 2	1	72.5	0.0	15.0	12.5	100.0
A nominated ER Team member should contact administrators of social media groups or pages that honour the deceased student, and ask them to monitor and remove any inappropriate (e.g. hostile or inflammatory) comments.	Re-rated in round 2	1	72.5	0.0	22.5	5.0	100.0

A nominated ER Team member should contact administrators of social media groups or pages that honour the deceased student, and ask them to report any concerning comments made by the school's students to a nominated ER Team member.	Re-rated in round 2	1	72.5	0.0	22.5	5.0	100.0
If required, a nominated ER Team member should contact the social media organisation directly to request they remove inappropriate messages or pages.	Re-rated in round 2	1	72.5	0.0	25.0	2.5	100.0
If the school has a website and/or social media presence, the ER Team should use these to proactively communicate with students, teachers, and parents about risk factors and warning signs of suicide in young people (e.g. Risk factors and warning signs).	No	1	65.0	0.0	27.5	7.5	100.0
The ER Team should provide students with links to suicide prevention or mental health information to post on their own social media pages.	No	1	62.5	5.0	27.5	5.0	100.0
A nominated ER Team member should encourage peers who have access to the deceased student's sites to monitor and report any concerning posts.	No	1	62.5	0.0	30.0	7.5	100.0
A nominated ER Team member should contact administrators of social media groups or pages that honour the deceased student, and ask them to block individuals from the site if necessary.	No	1	62.5	0.0	32.5	5.0	100.0
A nominated ER Team member should contact the family of the deceased student to ask them to report any concerning comments made by the school's students to a nominated ER Team member.	No	1	62.5	0.0	22.5	15.0	100.0

A nominated ER Team member should contact administrators of social media groups or pages that honour the deceased student, and ask them to allow a nominated ER Team member access to the page/group.	No	1	60.0	2.5	30.0	7.5	100.0
If required, a nominated ER Team member should contact parents to request the removal of inappropriate posts written by their child.	No	1	60.0	0.0	32.5	7.5	100.0
A nominated ER Team member should contact administrators of social media groups or pages that honour the deceased student, and ask them to distribute suicide prevention or mental health information.	No	1	57.5	5.0	32.5	5.0	100.0
The ER Team should ask selected students to monitor other students' use of social media and report any concerning content to a nominated member of the ER Team.	No	2	50.0	3.8	30.8	15.4	100.0
A nominated ER Team member should contact the family of the deceased student to offer help in monitoring the deceased student’s social media pages (e.g. Facebook accounts after a death).	No	1	47.5	5.0	32.5	15.0	100.0
A nominated ER Team member should seek the permission from the family of the deceased student before setting up an online memorial.	No	1	47.5	0.0	20.0	32.5	100.0
A nominated ER Team member should contact the family of the deceased student to ask them to block individuals from the site if necessary.	No	1	45.0	0.0	40.0	15.0	100.0
If the school has a website and/or social media presence, the ER Team should use these to proactively communicate with students, teachers, and parents	No	1	42.5	5.0	40.0	12.5	100.0

about the relationship between mental illness and suicide.							
If the school has a website and/or social media presence, the ER Team should use these to proactively communicate with students, teachers, and parents about the funeral or memorial service.	No	1	37.5	0.0	32.5	30.0	100.0
A nominated ER Team member should contact the family of the deceased student to ask them to monitor and remove any inappropriate comments.	No	1	37.5	0.0	37.5	25.0	100.0
A nominated ER Team member should discuss the type and timing of any online memorial with the close friends of the deceased student.	No	1	32.5	0.0	32.5	35.0	100.0
A nominated ER Team member should contact the family of the deceased student to ask them to distribute suicide prevention or mental health information.	No	1	20.0	5.0	40.0	35.0	100.0
If the ER Team establishes an online memorial it should be removed after students stop using it.	No	1	20.0	0.0	45.0	35.0	100.0
If the ER Team establishes an online memorial it should be removed after the funeral.	No	1	17.5	2.5	57.5	22.5	100.0
If the ER Team establishes an online memorial it should be removed never.	No	1	7.5	2.5	45.0	45.0	100.0
The ER Team should set up an online memorial page on the school website if this is requested by students or staff.	No	1	5.0	0.0	40.0	55.0	100.0
If the ER Team establishes an online memorial it should be removed after 1 month.	No	1	5.0	2.5	57.5	35.0	100.0

If the ER Team establishes an online memorial it should be removed after 2 months.	No	1	2.5	2.5	55.0	40.0	100.0
The ER Team should set up an online memorial page on a social media site if this is requested by students or staff.	No	1	0.0	0.0	45.0	55.0	100.0
Continued monitoring							
School staff should monitor high-risk students around the time of potentially distressing periods (e.g. coronial inquest, at anniversary dates such as the date of the suicide or the birthday of the deceased student).	Yes	1	95.0	0.0	5.0	0.0	100.0
The ER Team should talk to staff periodically about how to identify and support high-risk students in the weeks and months following the death.	Yes	1	95.0	0.0	2.5	2.5	100.0
The school welfare staff should meet with any students identified as requiring further support.	Yes	1	92.5	0.0	5.0	2.5	100.0
School staff should monitor high-risk students by communicating regularly with parents and any treating mental health professionals.	Yes	1	90.0	0.0	10.0	0.0	100.0
A nominated ER Team member should monitor the attendance, changes in behaviour, moods, choice of friends, and schoolwork/grades of high-risk students and follow up accordingly.	Yes	1	90.0	0.0	7.5	2.5	100.0
The ER Team should make students, families, and staff aware of the continuing availability of any support services.	Yes	1	90.0	0.0	7.5	2.5	100.0
Approximately 1 to 2 months after the death, the ER Team should organise the staff to review the status of high-risk students and determine the need for further intervention.	Yes	1	87.5	0.0	7.5	5.0	100.0

The ER Team should ensure external mental health professionals are kept informed of any significant changes in students who are clients of the mental health service.	Yes	1	87.5	5.0	5.0	2.5	100.0
The ER Team should continue to monitor any staff who were particularly distressed by the death around the time of potentially distressing periods (e.g. coronial inquest, at anniversary dates such as the date of the suicide or the birthday of the deceased student).	Yes	1	87.5	0.0	10.0	2.5	100.0
School staff should monitor high-risk students for at least 6 months and, less intensely, for 1 to 2 years thereafter.	Yes	2	80.8	0.0	19.2	0.0	100.0
A nominated ER Team member should meet with any staff identified as requiring further support.	Yes	2	80.8	3.8	11.5	3.8	100.0
School staff should monitor high-risk students for at least 6 months and, less intensely, for 1 to 2 years thereafter.	Re-rated in round 2	1	77.5	2.5	20.0	0.0	100.0
The ER Team should continue to monitor any staff who were particularly distressed by the death for at least 6 months and, less intensely, for 1 to 2 years thereafter.	Re-rated in round 2	1	77.5	2.5	17.5	2.5	100.0
The ER Team should meet with any staff identified as requiring further support.	Re-rated in round 2	1	77.5	0.0	17.5	5.0	100.0
The ER Team should continue to monitor any staff who were particularly distressed by the death for at least 6 months and, less intensely, for 1 to 2 years thereafter.	No	2	76.9	0.0	15.4	7.7	100.0
The ER Team should continue to monitor any staff who were particularly distressed by the death by communicating regularly with the staff member's supervisor.	No	1	75.0	0.0	20.0	5.0	100.0

The ER Team should meet periodically until the cohort of students complete their schooling.	No	1	62.5	5.0	22.5	10.0	100.0
The ER Team should continue to monitor any staff who were particularly distressed by the death by communicating regularly with the staff member's supervisor/manager.	No	2	57.7	11.5	30.8	0.0	100.0
The ER Team should meet with any staff identified as requiring further support.	No	2	38.5	7.7	19.2	34.6	100.0
Documenting actions							
A nominated ER Team member should clearly document all postvention actions taken by the school.	Yes	1	100.0	0.0	0.0	0.0	100.0
If the death occurs on school grounds, a nominated ER Team member should document what happened and the school's response.	Yes	1	100.0	0.0	0.0	0.0	100.0
The ER Team should ensure that any written information provided to the school community is time-dated because it can change over time.	Yes	1	97.5	0.0	2.5	0.0	100.0
A nominated ER Team member should document decisions and actions of the ER Team.	Yes	1	97.5	0.0	2.5	0.0	100.0
A nominated ER Team member should document identification of high-risk students and referrals for support.	Yes	1	97.5	0.0	2.5	0.0	100.0
The ER Team should ensure that all events, decisions and actions are documented as soon as possible.	Yes	1	95.0	0.0	5.0	0.0	100.0
A nominated ER Team member should document dates and times of all steps taken.	Yes	1	95.0	0.0	2.5	2.5	100.0
A nominated ER Team member should document the content of ER Team meetings.	Yes	1	95.0	0.0	5.0	0.0	100.0
A nominated ER Team member should document the content of parent meetings.	Yes	1	95.0	5.0	0.0	0.0	100.0

A nominated ER Team member should document the content of staff meetings.	Yes	1	92.5	5.0	2.5	0.0	100.0
A nominated ER Team member should document all communication with external agencies (e.g. police, coroner, mental health services).	Yes	1	92.5	2.5	5.0	0.0	100.0
The ER Team should ensure that all documentation is securely stored.	Yes	1	90.0	2.5	7.5	0.0	100.0
The ER Team should ensure that all documentation is limited to factual information and does not contain judgements, views or opinions.	Yes	1	87.5	0.0	12.5	0.0	100.0
The ER Team should ensure that all documents are signed by the person who prepared them.	Yes	1	82.5	5.0	12.5	0.0	100.0
The ER Team should ensure that any written information regarding the death provided to the school community states that "This is the correct information as we know it at this time".	Re-rated in round 2	1	77.5	5.0	12.5	5.0	100.0
The ER Team should ensure that any written information regarding the death provided to the school community states that "This is the correct information as we know it at this time".	No	2	61.5	3.8	23.1	11.5	100.0
Critical incident review							
The ER Team should use the outcome of any critical incident review to update the ER Plan.	Yes	1	100.0	0.0	0.0	0.0	100.0
Any critical incident review meeting should include a recognition of what challenged the team.	Yes	1	97.5	0.0	2.5	0.0	100.0
Any critical incident review meeting should include identification of future training needs for staff.	Yes	1	97.5	0.0	2.5	0.0	100.0
A nominated ER Team member should take notes of any critical incident review meeting, including all actions taken and recommendations.	Yes	1	97.5	0.0	2.5	0.0	100.0

Any critical incident review meeting should include information regarding where crisis responders can go to discuss their own reactions to the crisis and share their own emotional experiences.	Yes	2	96.2	0.0	3.8	0.0	100.0
The ER Team should conduct a critical incident review several months after the death, once the school has returned to equilibrium.	Yes	1	95.0	0.0	5.0	0.0	100.0
The review should include gathering data on staff views.	Yes	1	95.0	2.5	2.5	0.0	100.0
The review should include a meeting of the ER Team.	Yes	1	95.0	0.0	5.0	0.0	100.0
Any critical incident review meeting should include a review and evaluation of crisis intervention activities.	Yes	1	95.0	0.0	5.0	0.0	100.0
Any critical incident review meeting should include a review of whether the ER Plan addressed all critical components of postvention.	Yes	1	95.0	0.0	2.5	2.5	100.0
Any critical incident review meeting should include an acknowledgment of the efforts of the school community and discussion of what has worked well.	Yes	1	95.0	0.0	5.0	0.0	100.0
Any critical incident review meeting should include a development of a plan for follow-up actions.	Yes	1	95.0	0.0	5.0	0.0	100.0
Any critical incident review meeting should include development of an implementation plan for agreed actions.	Yes	1	95.0	0.0	5.0	0.0	100.0
The review should include compiling the data and feeding it back to the ER Team.	Yes	1	92.5	2.5	2.5	2.5	100.0
Any critical incident review meeting should include verbal appreciation for those who assisted in the postvention.	Yes	1	92.5	2.5	5.0	0.0	100.0

Any critical incident review meeting should include development of a communication plan for the review outcomes (e.g. who will be told and how).	Yes	1	92.5	0.0	7.5	0.0	100.0
A nominated ER Team member should ask staff for their views on how the school has managed the response to the suicide.	Yes	1	90.0	0.0	10.0	0.0	100.0
Before any review meeting, a nominated ER Team member must provide all participants with a summary of the documented actions to date.	No	2	76.9	11.5	11.5	0.0	100.0
Any critical incident review meeting should include an opportunity for crisis responders to discuss their own reactions to the crisis and share their own emotional experiences.	Re-rated in round 2	1	75.0	5.0	10.0	10.0	100.0
Before any review meeting, a nominated ER Team member must provide to all participants a summary of the documented actions to date.	Re-rated in round 2	1	72.5	0.0	27.5	0.0	100.0
A nominated ER Team member should ask staff for their views on the school culture prior to the suicide.	No	1	65.0	10.0	20.0	5.0	100.0
The ER Team should ensure that any written information regarding the death provided to the school community should emphasise that the information is the "correct information (or best evidence) known at that time".	No	2	61.5	7.7	19.2	11.5	100.0
Before any review meeting, a nominated ER Team member must provide to all participants data from any staff survey.	No	1	60.0	2.5	32.5	5.0	100.0
Any critical incident review meeting should be conducted by a nominated ER Team member.	No	1	50.0	2.5	37.5	10.0	100.0
Any critical incident review meeting should include an opportunity for crisis responders to discuss their own	No	2	50.0	0.0	23.1	26.9	100.0

reactions to the crisis and share their own emotional experiences.							
A nominated ER Team member should seek staff views via an anonymous survey.	No	1	40.0	5.0	50.0	5.0	100.0
A nominated ER Team member should seek staff views via a survey.	No	1	30.0	7.5	47.5	15.0	100.0
Any critical incident review meeting should be conducted by an external facilitator, e.g. from the relevant education department.	No	1	30.0	15.0	47.5	7.5	100.0
A nominated ER Team member should seek staff views via face to face interviews.	No	1	22.5	7.5	60.0	10.0	100.0
Future prevention							
ER Team members should be provided with opportunities for ongoing relevant training (e.g. in mental health, suicide prevention, crisis management).	Yes	1	95.0	0.0	5.0	0.0	100.0
The ER Team should consult with staff (e.g. through focus groups and/or surveys) to generate ideas for improving the health and wellbeing of the school community and work with them to implement these.	Yes	1	95.0	0.0	2.5	2.5	100.0
The relevant education department should endorse suicide postvention guidelines for use in schools.	Yes	1	92.5	2.5	0.0	5.0	100.0
The relevant education department should promote the uptake of any endorsed guidelines in schools.	Yes	1	92.5	5.0	0.0	2.5	100.0
The school should organise a general mental health awareness program to educate staff on symptoms, risk factors, and sources of help for common mental health problems in young people.	Yes	1	90.0	0.0	10.0	0.0	100.0

The school should organise workshops on stress reduction, coping techniques and positive mental health for students and staff.	Yes	1	90.0	0.0	7.5	2.5	100.0
The school should organise a suicide awareness program to educate staff on how to identify and refer students who may be at risk of suicide.	Yes	1	87.5	0.0	12.5	0.0	100.0
The school should organise more comprehensive training for school wellbeing staff on how to identify, assess and support students who may be at risk of suicide.	Yes	1	87.5	0.0	10.0	2.5	100.0
The school should organise a general mental health awareness program to educate students on symptoms, risk factors and sources of help for common mental health problems.	Yes	1	87.5	2.5	10.0	0.0	100.0
To restore the long-term wellbeing of students, the ER Team should audit current curriculum for any social and emotional health and wellbeing content and augment this where necessary.	Yes	1	87.5	2.5	10.0	0.0	100.0
To restore the long-term wellbeing of students, the ER Team should consult with students (e.g. through focus groups and/or surveys) to generate ideas for improving the health and wellbeing of the school community and work with student leadership groups/student council to implement these.	Yes	1	87.5	2.5	7.5	2.5	100.0
The ER Team should consult with parents (e.g. through focus groups and/or surveys) to generate ideas for improving the health and wellbeing of the school community and work with them to implement these.	Yes	1	85.0	2.5	10.0	2.5	100.0
The ER Team should provide parent sessions on mental health and wellbeing issues regularly throughout the year.	Yes	1	85.0	0.0	12.5	2.5	100.0

The school should organise a mental health awareness program about how to support a peer.	Yes	2	84.6	0.0	7.7	7.7	100.0
The ER Team should host small social events and school activities that build social cohesion and connectedness for the school community (e.g. Saturday school gardening and barbeque).	Re-rated in round 2	1	77.5	7.5	12.5	2.5	100.0
To restore the long-term wellbeing of students, the ER Team should purchase books on mental health for the school library and make suggestions for purchases by the local library.	Re-rated in round 2	1	70.0	10.0	17.5	2.5	100.0
The school should organise a suicide awareness program for students about warning signs, sources of help and how to support a peer.	No	1	55.0	2.5	30.0	12.5	100.0
The school should not implement suicide prevention activities until the school is ready to move forward.	No	2	53.8	3.8	26.9	15.4	100.0
To restore the long-term wellbeing of students, the ER Team should encourage participation in local community groups (e.g. Scouts, Girl Guides).	No	1	52.5	17.5	25.0	5.0	100.0
The ER Team should host small social events and school activities that build social cohesion and connectedness for the school community (e.g. Saturday school gardening and barbeque).	No	2	46.2	3.8	38.5	11.5	100.0
To restore the long-term wellbeing of students, the ER Team should purchase books on mental health for the school library and make suggestions for purchases by the local library.	No	2	42.3	19.2	26.9	11.5	100.0
The school should not implement suicide prevention activities earlier than 6 to 12 months after the death, and should wait until the school community has reached a sense of equilibrium.	No	1	25.0	2.5	50.0	22.5	100.0
